# Supplementary material for: Healthy Patients, Workforce and Environment: Coupling Climate Adaptation and Mitigation to Wellbeing in Healthcare
Source: Int J Environ Res Public Health. 2023 Nov 13;20(22):7059. doi: 10.3390/ijerph20227059 (PMC10671525; doi:10.3390/ijerph20227059)
Supplement: Supplementary file 1 [file ijerph-20-07059-s001.zip › Benefits of Landcare.pdf]

# MULTIPLE BENEFITS OF LANDCARE AND NATURAL RESOURCE MANAGEMENT

## FINAL REPORT

## 7 JULY 2013

Water | Energy & Resources | Environment | Property & Buildings | Transportation

# Executive summary

Our understanding of Landcare in Australia is missing a vital component. Although the environmental and agricultural outcomes have been well-explored, the many other benefits of Landcare and natural resource management (NRM) beyond these domains have, for the most part, been only anecdotally acknowledged.

Recognising this, the Australian Landcare Council commissioned an investigation of the benefits of Landcare and NRM that exist beyond the biophysical domain. The research was intended to establish the extent of the evidence base and to build this into a stronger case for investment in Landcare and NRM, both to ensure ongoing levels of funding and to gain support from outside the primary industries and environment sectors.

The findings of this research reveal an impressive array of multiple benefits. The literature review, interviews and case studies that underpin the findings identified six main categories of benefits, incorporating 21 sub-categories of benefits. These main categories, over and above the environmental and agricultural sustainability outcomes, are:

## Lifelong learning – well established and understood

The report makes a compelling case for a range of positive educational outcomes for individuals (for example, continuous learning and skill development) through to the broader community (for example, spreading awareness and delivering innovation). Landcare and NRM were seen to offer both formal and informal educational mechanisms, and often extended to areas of society that are traditionally difficult to reach.

The Friends of Narrabeen Lagoon case study, where northern Sydney residents worked to ensure the local catchment was properly and sustainably protected, highlighted the potential reach of these educational benefits through its awareness raising among the community and policy makers (which resulted in changes to government policy).

## Social—community health and wellbeing – complex but considerable

Landcare and NRM not only provide an avenue for a very real connection with the natural environment, but also lead to increased social networking and participation—both of which can contribute to physical and mental well-being. The agricultural and environmental outcomes of Landcare and NRM—a healthier living environment—also contribute to healthy individuals and communities.

The Upper Goulburn Landcare Network and Goulburn Broken Catchment Management Authority case study—a fire recovery project—demonstrates the capacity for Landcare and NRM to contribute to community health and well-being. In addition to directly aiding in the disaster recovery, this project allowed individuals to have meaningful contact with the environment and increased social connectedness and participation in community activities (including from urban dwellers and those not previously involved in Landcare or NRM).

## Social—political and social capital – a vital part of the social fabric

The dynamic social relationships and cohesion developed through Landcare and NRM can form an intrinsic part of the social fabric, in many cases filling gaps in the community beyond the agricultural and environmental domain. The benefits—particularly for regional and rural communities—include enhanced social capacity and cohesion, stronger local governance, the increased recognition of women in rural communities, and self-empowerment and fulfilment.

For example, the Naturally Resourceful workshops case study (run by the Queensland Murray Darling Committee and Mitchell Landcare) often had a profound impact on the way women operated in their local communities and catalysed representational opportunities for workshop graduates on local boards, councils and a range of community organisations.

## Economic – a considerable set of numbers

The report draws out that Landcare and NRM can generate an economic return in the order of 2-5 times the original investment. This economic benefit arises through access to labour, equipment, expertise and training, financial assistance, and increased farming profitability. The scale of the economic return is also important, with Landcare contributing to individuals as well as regions (including Indigenous communities) and providing a framework for investment and support on a larger scale.

In addition to increasing the productivity of the land, the case study exploring the Web of Trees farm forestry project (developed by the Otway Agroforestry Network) demonstrated economic benefits in the form of an alternative and diversified source of income as well as an increase in land values.

## Cultural – increasing connections in new ways that are very old

The report highlights the significant benefits a connection with country has for spiritual, social, physical and mental health—particularly in Indigenous communities. In some cases Landcare has helped to maintain or increase existing connections, while in others it has created new connections or re-created connections that existed prior to white settlement.

Two case studies highlight the cultural benefits of Landcare and NRM: the Friends of Narrabeen Lagoon case study (representing an urban setting) and the Roper River case study (a remote setting). These projects not only contributed to the preservation of and access to traditional Indigenous knowledge, but also to the understanding of traditional Landcare and NRM activity among the broader community.

## Resilience – resilient people, resilient landscapes

The report puts the view that resilient individuals, communities and landscapes are the end state of the multiple benefits of Landcare and NRM. Resilience in this case arises through the multiple benefits being evident, heavily integrated, interdependent and mutually reinforcing. This is strongly demonstrated in the case studies and literature

reviewed. In particular, Landcare promotes the formation of complex networks that allow communities to support each other and to can provide services beyond the agricultural and environmental domain when faced with adversity.

## The beneficiaries of multiple benefits

In addition to the traditionally recognised beneficiaries of Landcare and NRM, this report identifies an additional set of stakeholders who benefit in ways that have not been previously recognised or well articulated. This group of beneficiaries crosses all scales—from individuals to national level bodies—more truly represents the diverse beneficiaries of Landcare and NRM and aligns with the contemporary direction of NRM in Australia with its focus on resilience and linked socio-economic systems.

## Future directions

Multiple benefits and resilience research is an emerging area of both theory and practice and this report should be considered as a starting point in driving thinking, research and action. The evidence base for multiple benefits needs to be further developed, and this report suggests several indicators for doing so.

## Australian Landcare Committee (ALC) response

The Australian Landcare Council sees this investigation and the preparation of this report as a starting point to further understand and promote the broader benefits of Landcare and NRM. The evidence base for the multiple benefits needs to be further developed, with the Landcare community in a perfect position to contribute to the data already collected. The council will communicate the findings of this report, consider possible methodologies for developing the evidence base and making it available to all, seek to further refine the indicators of multiple benefits, and provide advice to government on the findings of this report. As the evidence base develops and our understanding of the multiple benefits of Landcare and NRM grows, the council believes a strong case will emerge for increased and co-investment in Landcare and NRM, and for greater collaboration across government portfolios and the various sectors of the community.

# Table of contents

**1. Introduction ........ 1**
1.1 Purpose of this report ........ 1
1.2 Defining multiple benefits ........ 1
1.3 Project approach ........ 2

**2. Natural Resource Management and Landcare ........ 6**
2.1 Definitions ........ 6
2.2 A brief history ........ 7
2.3 Landcare in operation ........ 8
2.4 Key achievements of Landcare ........ 10
2.5 Moving towards multiple benefits ........ 12

**3. Multiple benefits of Landcare and NRM: The literature ........ 13**
3.1 Categories of multiple benefits ........ 13
3.2 Learning, awareness and practice change ........ 14
3.3 Social – community health and wellbeing ........ 17
3.4 Social – political and social capital ........ 20
3.5 Economic ........ 24
3.6 Cultural ........ 26
3.7 Resilience ........ 27

**4. Multiple benefits of Landcare and NRM: The practice ........ 29**
4.1 Summary of interviews ........ 29
4.2 Case studies ........ 32

**5. Multiple benefits of Landcare and NRM: A synthesis ........ 54**
5.1 Overview of multiple benefits and outcomes ........ 54
5.2 Key stakeholder beneficiaries ........ 58
5.3 Resilience – the end game of multiple benefits? ........ 60
5.4 Measuring multiple benefits – suggested key measurable indicators ........ 64

**6. Summary of findings ........ 69**
6.1 The multiple outcomes and benefits (social, economic, cultural, health, learning, awareness and practice change and community) that result from Landcare and NRM ........ 69
6.2 The contribution of the outcomes and benefits to building community resilience and capacity to handle major challenges ........ 70
6.3 How can multiple benefits and outcomes be monitored to demonstrate returns on NRM/Landcare investment ........ 71
6.4 How should the multiple benefits and outcomes be communicated to agencies and organisations outside the NRM sector? ........ 72
6.5 Recommendations ........ 74

# Table index

Table 1: Multi-criteria selection matrix for case study selection .................................. 4
Table 2: Categories of multiple benefits ................................................................ 14
Table 3: Summary of ideas to measure multiple benefits from interview process ......... 32
Table 4: Multiple benefits of the Fire Recovery Project ............................................ 36
Table 5: Multiple benefits of a Web of Trees ......................................................... 40
Table 6: Multiple Benefits of the Naturally Resourceful program ............................... 43
Table 7: Multiple benefits of creating a Sustainable Catchment program .................... 46
Table 8: Multiple benefits of the Mangarrayi Rangers .............................................. 49
Table 9: Stakeholders who derive value from the multiple benefits of Landcare and NRM ........................................................................................................... 59
Table 10: Interaction of selected key resilience principles with multiple benefits of Landcare and NRM ........................................................................................ 62
Table 11: Description of indicators ...................................................................... 67
Table 12: Categories of multiple benefits .............................................................. 69
Table 13: Description of indicators ...................................................................... 71

# Figure index

Figure 1: Summary of project approach ................................................................. 2
Figure 2: National Landcare Network .................................................................... 10
Figure 3: Logic demonstrating links between Landcare activities and improved health and wellbeing ........................................................................................................ 17
Figure 4: Naturally Resourceful Workshop locations ............................................... 42
Figure 5: A conceptual framework for regional level monitoring and reporting of social resilience ........................................................................................................ 65
Figure 6: Links between indicators and categories of multiple benefits ...................... 66
Figure 7: Contribution of multiple benefits to attributes of a resilient system .............. 70

# Appendices

**Appendix A References**

**Appendix B EEA: A synthesis of supporting evidence**

**Appendix C Interview questionnaire**

**Appendix D Consultation list**

**Appendix E Case study framework**

**Appendix F Role and membership of the ALC**

This report has been prepared by GHD for Department of Agriculture and may only be used and relied on by Department of Agriculture for the purpose agreed between GHD and the Department of Agriculture as set out in Section 1.1 of this report.

GHD otherwise disclaims responsibility to any person other than Department of Agriculture arising in connection with this report. GHD also excludes implied warranties and conditions, to the extent legally permissible.

The services undertaken by GHD in connection with preparing this report were limited to those specifically detailed in the report and are subject to the scope limitations set out in the report.

The opinions, conclusions and any recommendations in this report are based on conditions encountered and information reviewed at the date of preparation of the report. GHD has no responsibility or obligation to update this report to account for events or changes occurring subsequent to the date that the report was prepared.

The opinions, conclusions and any recommendations in this report are based on assumptions made by GHD described in this report (refer section 1.3 of this report). GHD disclaims liability arising from any of the assumptions being incorrect.

GHD has prepared this report on the basis of information provided by Department of Agriculture and others who provided information to GHD (including Government authorities)], which GHD has not independently verified or checked beyond the agreed scope of work. GHD does not accept liability in connection with such unverified information, including errors and omissions in the report which were caused by errors or omissions in that information.

# 1. Introduction

## 1.1 Purpose of this report

To date the outcomes achieved by Natural Resource Management (NRM) and Landcare[1] programs and projects have mostly been reported in biophysical areas, with much less information about their social and economic contributions. In response, the Department of Agriculture and the Australian Landcare Council (ALC) commissioned GHD to undertake a project to investigate the benefits of Landcare and NRM beyond the biophysical domain. This report is the key output from the project and aims to:

- identify the multiple stakeholders benefiting from Landcare and NRM
- contribute to an evidence base for the multiple and unrecognised benefits of Landcare and NRM
- assist in establishing a value proposition for Landcare and NRM that will build support from agencies and organisations outside the NRM sector and provide a case for investment
- suggest key measurable indicators which can be used to monitor and report on multiple outcomes and benefits, so that returns on future Landcare and NRM investment can be demonstrated.

The report is structured to address the above objectives. Section 1 provides a general introduction including definitions and an overview of the project approach. Owing to the potential broad readership of the report, the introduction is followed by some background material on Landcare and NRM (Section 2). Sections 3 and 4 detail initial project findings which are drawn together to deliver an outcome aligned with the project objectives in Section 5. Section 6 contains a summary and recommendations.

## 1.2 Defining multiple benefits

For the purpose of this report, GHD has adopted the multiple benefits definition provided by the Department of Agriculture and the ALC, which is as follows:

> "multiple benefits (sometimes called co-benefits) refer to positive impacts or benefits that are additional to the primary intended benefits for which an NRM or Landcare investment is made. These multiple benefits can affect multiple stakeholders, can be both intended and unintended, and may not have been previously recognised, valued, measured or reported. They include social and community, health and wellbeing, resilience and recovery, cultural, socio-political, economic, environmental and ecosystem benefits."

---

[1] There is sometimes discussion over the use of "small 'l' landcare" which generally covers integrated land and water management (i.e. the ethic part of the definition) and "big 'L' Landcare" being the community movement and its many institutions, programs and initiatives (the movement and the model part of the definition). We use Landcare in its broadest context and in keeping with the definition. Where the report uses "landcare" it is explicitly referring to land management practices.

## 1.3 Project approach

The project objectives were delivered in three main steps comprising a comprehensive literature review, a series of interviews and case studies followed by a synthesis of findings based on the literature, interviews and case studies (Figure 1). The main output of the project is a report addressing the project objectives, as noted in Section 1.1.

**Figure 1: Summary of project approach**

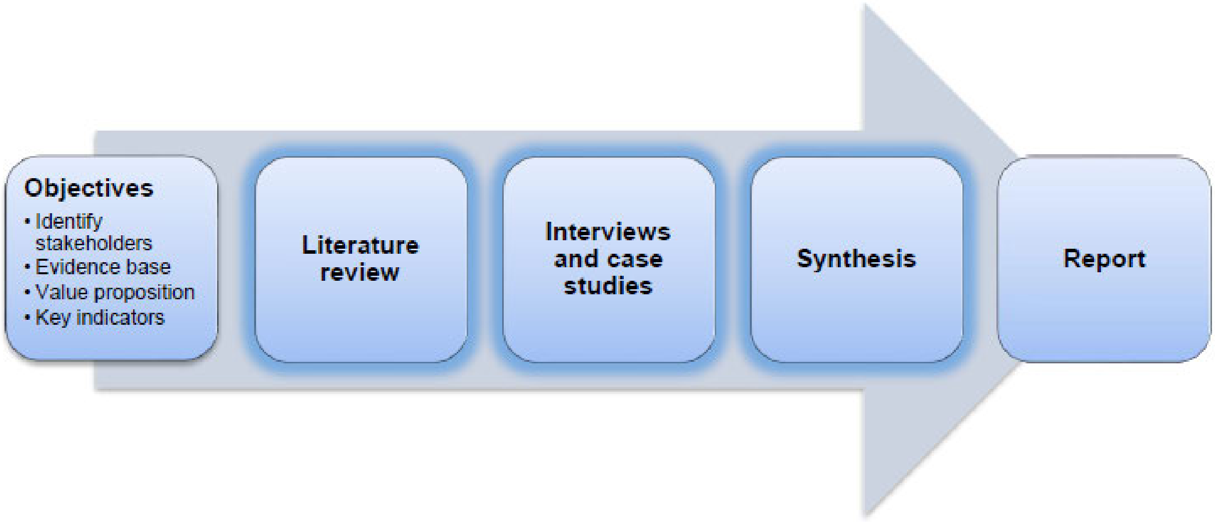


### 1.3.1 Literature review

The literature review was conducted by Environmental Evidence Australia (EEA) to establish a baseline of the published information about multiple benefits which result from Landcare and NRM. Relevant literature was searched, stored and broadly synthesised.

The evidence search used a range of methods across various sources (web based international databases, web search engines, electronic searches of key individuals and key organisations). All cited evidence was uploaded into an electronic Zotero evidence base to enable future access or further enquiry (Appendix A). Tabulated search results and the initial findings of the literature review are located at Appendix B.

### 1.3.2 Interviews

Twenty seven semi-structured interviews were completed to gain an understanding of the social, economic, cultural, health, learning, awareness and practice change and community outcomes and benefits and how these contribute to building community resilience and capacity to handle major challenges.

Interview participants were initially suggested by GHD and then refined in consultation with the Department of Agriculture and the ALC, with the final list of participants covering the following sectors:

- agencies
- regional NRM groups

- Landcare networks and associations (national, state and regional/local levels)
- educational institutions
- non-government organisations
- regional environmental and farming groups
- local government and community groups
- prominent individuals with long term knowledge of Landcare and NRM.

The interview questionnaire appears at Appendix C and the list of interview participants at Appendix D.

### 1.3.3 Case studies

An initial list of case studies was prepared based on suggestions from the ALC and GHD. A nationwide call for case studies was then made via an email to the Regional Landcare Facilitators Network. A consolidated "long list" of case studies was then prepared and reviewed by GHD, the Department of Agriculture and the ALC using a multi-criteria approach. This resulted in a short list of five case studies which were selected for detailed review.

The multi-criteria approach was utilised to select case studies to provide a transparent way to identify projects that were thought to best demonstrate measurable benefits and outcomes of Landcare and NRM. The following criteria were used:

- categories of multiple benefits
- program areas
- integration with other sectors (organisations/activities)
- geographical locations and social variables
- availability of information and other project considerations

Table 1 outlines the multi criteria selection matrix that was used to prioritise case studies. It is important to note that the initial findings and evidence base of the literature review led to a refinement of the categories of multiple benefits after the case studies were selected. This did not impact on the case study findings, but explains the slight difference in the criteria for selection of case studies and the way multiple benefits are detailed in the rest of this report.

**Table 1: Multi-criteria selection matrix for case study selection**

| Case study program area | Categories of multiple benefits* | Integration with other sectors |
|---|---|---|
| Coastcare<br>Rangelands<br>A significant Landcare Network<br>Urban / Bushcare<br>Cultural | Education<br>Social Capital<br>Resilience<br>Economic<br>Cultural | Agencies<br>Regional NRM Groups<br>Landcare networks and associations<br>Educational institutions<br>Non-government organisations<br>Regional environmental groups<br>Farming groups<br>Local government and community groups<br>Commercial organisations<br>International Landcare projects<br>Prominent individuals |

* These categories were accurate at the time the case studies were selected, but vary slightly from the final categories that are used in the report and which are summarised in Section 3.1.

The five case studies chosen for this project were:

1. Upper Goulburn Landcare Network, Victoria – Fire Recovery Project (site visit included)

2. Queensland Murray Darling Committee and Mitchell Landcare, Queensland – Naturally Resourceful Program (conducted by phone)

3. Otway Agroforestry Network, Victoria – A Web of Trees: Yan Yan Gurt Creek Catchment (site visit included)

4. Friends of Narrabeen Lagoon Catchment, NSW – Narrabeen Lagoon Activities program (site visit included)

5. Roper River Landcare Group – Building Capacity to Protect the Cultural and Production Values of Mangarrayi Traditional Lands (site visit included)

Further detail on the case studies is provided in Section 4.2.

### 1.3.4 Evidence synthesis

The synthesis of evidence was carried out throughout the project, but was particularly emphasised towards the later stages. This included a workshop between GHD and EEA which reviewed available evidence (literature review, case studies and interviews), developed potential indicators and considered the broader value proposition for Landcare and NRM.

The synthesis of evidence was also a particular focus during the development and review of this report. As part of the review of the draft report, the paucity of published literature in some of the multiple benefit categories emerged as an issue. This led to a change in emphasis of the report's findings with greater importance now being placed on the

evidence provided from case studies and interviews versus that present in the published literature.

# 2. Natural Resource Management and Landcare

Various definitions exist for Landcare and Natural Resource Management (NRM). This variation does not generally cause any significant issues and to some extent "goes with the territory." However, the broad target audience of this report means that some definitions and high level background in Landcare and NRM is necessary, as is some detail on their broad achievements to date.

## 2.1 Definitions

The most current and wide ranging definition of Landcare is defined in the Australian Framework for Landcare (Australian Framework for Landcare Reference Group, 2010) which states that Landcare is comprised of:

- **An ethic** – a philosophy, influencing the way people live and work in the landscape while caring for the land (soil, water and biota)
- **A movement** – local community action founded on stewardship and volunteerism, putting the philosophy into practice
- **A model** – a range of knowledge generation, sharing and support mechanisms including groups, networks (from district to national levels), facilitators and coordinators, government and non-government policies, structures, programs and partnerships influencing broad-scale community participation in sustainable resource management

The above definition puts Landcare in its broadest terms and is the approach used within this report. All natural resource care activities and projects are encompassed within this definition, including those carried out by Landcare groups, Landcare networks, Bushcare, Coastcare, Rivercare, Dunecare, friends of groups, non-government organisations, Indigenous groups, producer groups, environmental groups and educational institutions.

### 2.1.1 Natural Resource Management

We define NRM as the way in which people and natural landscapes interact, and how individuals, groups, institutions and governments deal with the complex and intimate interdependence of delivering economic, environmental and social outcomes. NRM operates from the micro scale to the global scale and is sometimes used as one way to help solve wicked problems.[2] In Australia, NRM is being increasingly viewed in a systems context using principles of resilience thinking and linked socio-ecological systems (systems of people and nature).

### 2.1.2 Resilience

Resilience thinking concepts work well with Landcare and NRM. A common definition of resilience is the ability of a system to tolerate disturbance and reorganise so as to have

[2] Wicked problems have a range of definitions. The Australian Public Service Commission (2007) definition is used here: those problems that go beyond the capacity of any one organisation to understand and respond, and where there is often disagreement about the causes of the problems and the best way to tackle them.

essentially the same function, structure and feedback, that is to have the same identity (Walker and Salt, 2012). With Landcare and NRM, resilience is particularly concerned with the interaction and management of self-organising systems as well as their thresholds, adaptation and transformation.

## 2.2 A brief history

Landcare and NRM share many common elements and in many situations in Australia it is reasonable to see them as interdependent. Certainly they have run hand in hand, and achieved significant outcomes, since the establishment of the Landcare movement in the mid-1980s.

The first legislation in Australia with a focus on NRM was arguably the NSW Western Lands Act (1901), which was put in place following widespread land degradation and soil erosion after the then record drought, and overgrazing by stock and feral animals. Soils and land degradation remained the focus of NRM efforts in Australia for a considerable period thereafter. As examples:

- in the 1930s, researchers developed the first guidelines for restoring degraded farmland and various agencies with a responsibility for soil conservation were established
- in 1946, a Premiers Conference established a standing committee on Soil Conservation
- after various research projects, the National Soil Conservation Program (NSCP) commenced in 1983

In the 1980s, the NRM agenda broadened considerably and became more formalised and increasingly focussed on community engagement. Landcare also commenced in the 1980s, kicking off in a formal sense in Victoria in 1986, and becoming a national initiative three years later via the historic National Farmers' Federation (NFF) and Australian Conservation Foundation (ACF) partnership. In 1989, the Hawke Government declared the 1990s the Decade of Landcare and announced significant funding via the National Landcare Program. Following the Decade of Landcare, the Australian Government continued to support Landcare via a range of programs and initiatives including the Natural Heritage Trust, Caring for our Country and the Biodiversity Fund.

At the national level, Landcare commenced as a unique partnership between two key national non-government organisations, various levels of government and the wider Australian community. The Australian Government initiatives have been supported over the years by the States and Territories and also via corporate sponsorship and philanthropy which has mostly been delivered by Landcare Australia Limited.

Whilst Landcare has had its share of difficulties and government support has waxed and waned, it has matured into a very broad movement that is likely to remain part of Australian society for the long term. Challenges and opportunities still remain and are neatly encapsulated in the recent Community Call for Action which urged all Australians to take responsibility for the way they live in the landscape.

The development of Landcare and NRM in Australia share some common elements; both started small and through various levels of community and government support have evolved into something that is a unique community and government partnership. The

partnership has moved through stages including attitude change and awareness building, community engagement, formal institutions and more recently into a broader agenda that seeks to involve more people in more diverse areas.

Much of the current agenda in Landcare and NRM is underpinned by a drive towards the use of resilience thinking, linked socio-ecological systems and the need for ongoing efficiency, effectiveness and impact. Assessing the benefits of Landcare and NRM from a multiple benefits perspective dovetails very well with this agenda; to date most of the achievements of Landcare and NRM have been considered somewhat narrowly, with the emphasis being on assessing the contributions made towards improving the condition of the natural resource base and community engagement in Landcare and NRM.

## 2.3 Landcare in operation

Landcare encompasses a diverse range of formal and informal institutions. These include Landcare groups, Landcare networks (district, regional and national levels), local government, regional NRM groups, state and territory governments, the Australian government, non-government organisations, companies and individuals. Landcare programs and activities range from large scale national and international programs through to small scale capacity building projects worth a few hundred dollars.

Landcare activities are undertaken by Landcare aligned groups, friends of and other independent groups as well as statutory and non-statutory NRM organisations across Australia. It is not relevant to detail all Landcare-related organisations in this report. Instead the following sections provide a sample of the depth and breadth of Landcare operations:

- across government
- in its role as a key advisor
- in a non-government setting
- as a coordinator of effort and on ground action

### Australian Government

The Australian Government recognises the important role Landcare and collective community action plays in the sustainable management of Australia's environment and natural resources. Support is provided to Landcare delivery via the ALC and its Secretariat, the Caring for Our Country initiative and other activities of the Department of Agriculture and the Department of Sustainability, Environment, Water, Population and Communities (SEWPAC).

One indication of the scale at which Landcare operates is the range of Australian Government funded staff that are responsible for directly delivering Landcare outcomes. These include:

- Australian Government NRM Officers who work at a state or territory level to help governments, regional bodies, Landcare and community groups, and other NRM organisations understand the opportunities through Caring for our Country, to support the delivery of other programs and projects and to inform policy makers on regional issues.

- The National Landcare Facilitator who works with the Landcare movement and other NRM stakeholders, advocating the Landcare ethos and supporting community Landcare through an advisory role. The National Landcare Facilitator has a special focus on sustainable production in the primary industry sector, together with the engagement and participation of community groups in NRM programs.

- Regional Landcare Facilitators who are funded through the Caring for our Country initiative. The Australian Government funds one full-time equivalent Regional Landcare Facilitator position in each of the 56 NRM regions (Department of Agriculture, 2012c). Regional Landcare Facilitators promote the uptake of sustainable farm and land management practices. They also establish, assist and develop community Landcare and production groups so that those groups can help share information and provide support to farmers and other land managers to meet challenges such as climate change (Department of Agriculture, 2012b).

## Australian Landcare Council

The ALC is a national advisory body which provides advice to the Australian Government on Landcare and matters concerning NRM.

The issues the ALC considers include insight into future opportunities and ensuring the Landcare movement and Australian community can meet the challenges of food security, climate variability, supporting volunteers and maintaining the environment (Department of Agriculture, 2012a). The ALC supports the implementation and promotion of principles in the Australian Framework for Landcare and the Community Call for Action and is responsible for overseeing the five-year (mid-term) review of the Australian Framework for Landcare and the Community Call for Action on behalf of the Landcare community.

## Landcare Australia Limited

Landcare Australia Limited (LAL) is a not-for-profit company that raises awareness and sponsorship support for the Landcare, Junior Landcare and Coastcare movements. LAL receives funding from various sources, including governments (Department of Agriculture, SEWPAC, and some state government support), corporate organisations (through tailored partnerships) and private donations.

LAL is supported by the Landcare Australia Limited Board of Directors, the Landcare Australia Limited Advisory Council and the Landcare Australia Limited Steering Committee which oversees key deliverables against LAL's contract with the Commonwealth.

## National Landcare Network

The National Landcare Network is a coalition of state and territory organisations representing Landcare, including Victorian Landcare Council, Tasmanian Landcare Association, Queensland Water and Land Carers, Landcare South Australia, Landcare ACT and Landcare NSW Inc. (Figure 2).

**Figure 2: National Landcare Network**

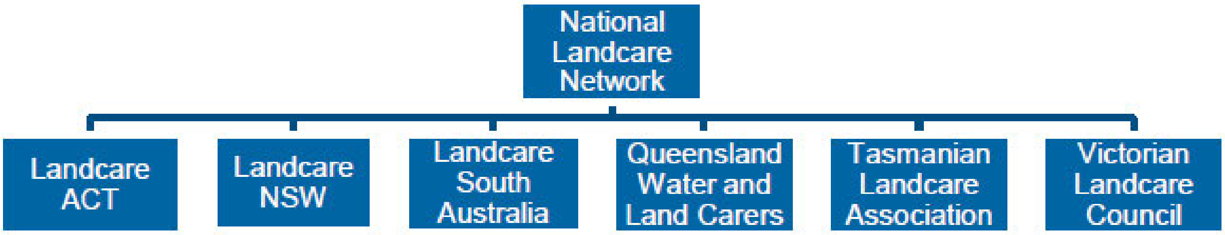


The National Landcare Network's website lists its primary charter to:

- foster a cohesive and cooperative forum to collaborate, support, advocate for and add value to Landcare and other community volunteer NRM groups (Landcare, Coastcare, etc) to address strategic and proactive NRM and environmental issues
- foster strategic partnerships between Landcare groups and the broader NRM and environmental stakeholders including: regional NRM organisations; governments; industry groups; Indigenous organisations and community groups; and other non-government community groups
- celebrate the achievements of Landcare across Australia and promote Landcare and community based NRM organisations and activities
- identify, communicate with and represent community based Landcare at the national level to develop and foster ideas, knowledge and resources
- speak as the national voice in the development of Landcare and broader NRM and environmental policy.

## 2.4 Key achievements of Landcare

While there has been some difficulty measuring long term change in the status of Australia's natural resources, a range of program evaluations leave little doubt that Landcare, NRM programs and the Landcare/NRM partnership has been an outstanding success. The last major reviews revealed around 6,000 Landcare groups across Australia (Department of Agriculture, 2009) and that over 140,000 or 94% (ABS, 2006-07) of farmers had delivered some type of NRM activity. By November 2010, over $1.7 billion had been committed to support new projects involving farmers, Indigenous groups, regional NRM organisations, Landcare and other volunteer environmental groups across Australia.

Landcare has been instrumental in achieving broad-scale community involvement and improved systems of sustainable resource use and management across Australia. The many recognised environmental benefits and achievements are outlined in the Australian Framework for Landcare (Department of Agriculture, 2010) including:

- planted millions of trees, shrubs and grasses
- repaired riparian zones and restored water quality by reducing erosion and fencing out stock from riverbanks

- protected remnants of native vegetation
- regenerated areas to provide habitat for native wildlife
- improved ground cover, grazing methods and soil management
- rehabilitated coastal dunes and recreational areas.

Along with the environmental focus, Landcare incorporates a strong social aspect. Communities have understood the benefits of joint action to analyse and solve local problems, including many that are beyond the capacity of individuals to solve. This has been vital in providing social cohesion and support structures in rural communities struggling to survive in the face of economic and environmental pressures. In this sense, Landcare has made a considerable contribution to the health and welfare of local communities (Department of Agriculture, 2010).

Achievements directly attributable to Landcare are summarised in Evolution of Landcare in Australia (Love, 2012) which recognises that Landcare has:

- provided an essential vehicle to assist a nation to change direction and work towards ecologically sustainable development
- involved more than 5000 community-based Landcare and related groups currently operating
- harnessed major community in-kind and financial investment through broad-scale community participation in sustainable resource management for the long term
- supported intergenerational learning through group corporate knowledge, family knowledge and school activities
- enabled thousands of people across communities since the 1980's to develop their capacities in skills, knowledge and application that has delivered outcomes including:
  - the repair of land degradation on private and public land across the country including soil erosion, water quality and ecological decline
  - the prevention of further degradation to the natural resource base
  - the uptake of resource management practices integrated into food and fibre production
  - a sense of responsibility outside landholder property boundaries
  - better linkages and integration between Aboriginal caring for country and European land management cultures and their people
  - opportunities for Aboriginal people to reconnect with country
  - an understanding of the changes required to reduce greenhouse gas emissions, manage climate change adaptability and water quality and availability while maintaining food and fibre security

– social cohesion and community resilience across regions through incorporating social, economic, environmental and cultural considerations into everyday activities that also assist disaster recovery in farming and pastoral communities

– positioning Australia as a world leader of a national community-based process that has successfully shifted attitudes and practices at the local level where the application of change actually needs to take place.

Results from the Health of the Landcare Movement Survey (De Hayr, 2012) concluded the vast majority of individual farmers and groups surveyed felt Landcare was still relevant to the future and that farmers considered Landcare to have a major role in responding to challenges such as food security, environment and climate change adaptation.

Evaluations of NRM investment also report very positive outcomes. Recent results from the review of the Australian Government's Caring for our Country Program as well as the Program's Annual Reports indicate the significant on ground results from NRM investment across Australia over the last five years.

## 2.5 Moving towards multiple benefits

The above sections have provided some indication of the history and scope of Landcare and NRM and their achievements to date. While these achievements have been significant, and have helped to improve the condition of the natural resource base, little has been done to measure successes and contributions in line with the broader goals that fit the emerging agenda of Landcare and NRM, that is, to assess their multiple benefits and outcomes.

Assessing the multiple benefits and outcomes of Landcare and NRM is the focus of the remaining sections of this report. Before doing this, it is necessary to briefly comment on the scales[3] of multiple benefits of Landcare and NRM which are the focus of the report.

As noted in Section 1.2, the definition of multiple benefits used is broad and functions at a range of scales. The spatial focus of the bulk of the literature and case studies reviewed as part of this project at individual, groups, local community and regional level. At institutional scale, multiple benefits have tended to be reported at group, regional and national scale and so these areas are also focussed on in this report. The report considers these scales in an integrated way and so focuses at groups, local community and regional scale, with come coverage of national matters.

[3] In this report, scale refers to spatial and institutional scale and not to the temporal dimension.

# 3. Multiple benefits of Landcare and NRM: The literature

This section presents a summary of the findings of the multiple benefits of Landcare and NRM, as reported in the literature evidence synthesis conducted by Environmental Evidence Australia. The full synthesis appears at Appendix B.

A plethora of what can be called Landcare’s success in achieving NRM change has been reported in various evaluations (Curtis and De Lacy, 1995; Curtis et al., 1993; Department of Agriculture, 2003; Edmonson, 2010; Horvath, 2001; Woodhill, 1992; Youl, 2006), in forums and workshops (Landcare Victoria and Department of Sustainability and Environment, 2008a, 2008b, 2008c, 2008d) and via various interviews and studies. Each case study or story is a small slice through the history of Landcare and NRM in Australia.

The successes of these small slices of Landcare and NRM are influenced by the local context, individuals, communities and broader Landcare arrangements. The literature reveals that these successes can be expressed in ways well beyond the number of volunteers involved and the amount of on ground NRM work completed. This has been demonstrated in the literature by a diverse range of linked socio-economic benefits that are often delivered in addition to the predicted NRM outcomes.

## 3.1 Categories of multiple benefits

The synthesis of evidence summarises the multiple benefits of Landcare and NRM into the following categories:

- learning, awareness and practice change
- social – community health and wellbeing
- social – political and social capital
- economic
- cultural
- resilience.

The categories for multiple benefits of Landcare and NRM are not discrete, but instead are heavily integrated and interdependent. For example, it is difficult to discuss mental health outcomes without considering these to be also factors of individual resilience. Similarly there is a strong interdependency between social capital and learning, awareness and practice change as there is between learning, awareness and practice change and economic benefits.

Table 2 outlines the key categories identified through the literature review and the sub-categories for which there was sufficient evidence of multiple outcomes and benefits. These categories were reviewed and refined as the project progressed and are used throughout this report. The remainder of Section 3 summarises the findings of the literature review against these multiple benefit categories.

**Table 2: Categories of multiple benefits**

| Categories | Sub-categories |
|---|---|
| Learning, awareness and practice change | Awareness raising<br>Practice change<br>Multigenerational reach<br>Improved knowledge<br>Scales of change<br>Continuous learning |
| Social – community health and wellbeing | Contact with natural environment<br>Social networks<br>Physical and mental health benefits |
| Social – political and social capital | Partnerships and networks<br>Leadership and public participation<br>Governance and self-regulation<br>Localism and empowerment<br>Increasing the recognition of women in rural communities<br>Personal growth<br>Filling the void<br>Increasing awareness, skills and knowledge |
| Economic | Increased financial return<br>Access to resources<br>Training and management techniques |
| Cultural | Connection with Country |
| Resilience | Resilient people and resilient landscapes |

## 3.2 Learning, awareness and practice change

In one of his earlier papers, Campbell stated that "many committed, far-sighted people are involved in Landcare. They are gaining intellectual stimulation, exciting new knowledge and the satisfaction of doing something constructive in their own district and of influencing others" (Campbell, 1992). While made in the very early years of Landcare, the sentiments expressed in this statement have continued to hold true in the twenty years since it was made.

Landcare is widely recognised as a movement that has fundamentally shifted the perceptions of land stewardship through increased awareness and knowledge of the landscape and the relationship of people to that landscape. Landcare has provided highly effective coordinated opportunities at a range of scales for experimentation, learning, increased awareness, observation, and skill development (Curtis and Sample, 2010; Curtis et al., 2008, 2000). ABARE surveys show that as many as 50% of all farmers have utilised Landcare groups for information regarding farm management, demonstrating

that Landcare has been a major catalyst for practice change and increased adaptive management (Department of Agriculture, 2003).

Of key contemporary relevance is the role that Landcare has played in enhancing state and territory-based agricultural education and extension services and at least in part filling the void left as a result of the decline in the funding of government extension, and the need for those services to seek alternative forms of funding and more efficient methods of technology transfer. The Landcare model can be an effective mechanism to facilitate the transfer of knowledge in partial response to declining extension funding (Cary and Webb, 2000; Vanclay and Lockie, 2000; Walker, 2000).

The following sections provide a summary of how the literature supports the learning, awareness and practice change benefits arising from Landcare and NRM.

## Awareness raising

- Many reviews have found that Landcare funding has been effective in raising awareness and that Landcare has been a valuable way to deliver information (Cary and Webb, 2001; Curtis, 1999; Walker, 2000) and change behaviour (Curtis and De Lacy, 1996a; Curtis, 1995a; Walker, 2000).
- There is very strong evidence that Landcare participation leads to significantly higher levels of awareness and concern about a range of land and water degradation issues. (Curtis and De Lacy, 1996a). Of note, non-Landcare participants acknowledge the wealth of knowledge about land and water degradation and sustainable farming practices that Landcare groups hold (Curtis et al., 2008).
- Sobels and Curtis (2001) report that increased awareness of Landcare is evidenced by growth in the Landcare movement and the widespread involvement in community environmental monitoring.

## Practice change

- Curtis (2003) reports that there is strong evidence that participation in NRM activities is a precursor to the accomplishment of program outcomes.
- When compared to other farmers, those involved in Landcare groups attend more field days and demonstration sites and undertake significantly higher amounts of on-ground work related to tree planting, fencing to manage stock access to waterways, and pest animal and weed control (Curtis and De Lacy, 1996a; Curtis, 1995b; Curtis et al, 2000; Curtis et al, 2008).
- Mues et al. (1998) and Curtis (2003) report that Landcare members were at least twice as likely as non-members to participate in Landcare group workshops and field days, industry grower groups and property management planning activities, establish annual priorities, develop catchment plans, implement best practice farming, interact with peers in innovative ways and they also accomplish significantly higher amounts of on-ground work.
- Landcare has encouraged farmers to appraise problems more holistically, which often leads to new methods for tackling these issues (Lockie, 1998; Youl et al., 2006).

## Multigenerational reach

- Love (2012) reports that Landcare has supported intergenerational learning through group corporate knowledge, family knowledge and school activities.

- Evidence suggests that when the Landcare ethic and practices are embedded in school curricula, not only do children take these on board and run with them, but they also influence their parents, other family members, and other children. Important in this concept is the realisation that both the children and their families may belong to sectors of the community that Landcare has traditionally found hard to reach (Landcare Victoria and Department of Sustainability and Environment, 2008).

- Landcare networks are increasingly taking a community leadership role and are well positioned to influence the community on a greater geographical scale as well as engage with the private sector, industry, schools and local government (Landcare Victoria and Department of Sustainability and Environment, 2008).

## Improved knowledge

- When compared to non-Landcare participants, Curtis (2003) reports that Landcare participants show significantly higher levels of knowledge of land and water degradation processes and sustainable farming practices recommended mitigating or preventing the degradation of natural resources.

- Community monitoring activities have resulted in the development of new technology and equipment, demonstrating that Landcare monitoring groups can be an important source for NRM innovation (Campbell, 1995).

- The Decade of Landcare increased the level of information and understanding of landscape processes, resources assessments, national scale data collections and standards, decision support programs and the interaction between agricultural systems, natural systems, land and water resources processes (Walker, 2000).

- Landcare promotes learning between rural landholders by engaging them in activities with each other, providing them with the opportunity to learn with their peers, to learn by doing, and to reflect on shared experiences (Curtis and Sample, 2010).

## Scales of change

- Landcare has helped natural resource managers recognise the need for management at greater spatial scales, for integrated NRM and has supported the establishment of institutional arrangements to enable integration to occur (Walker, 2000).

- Landcare has an ability to self-organise and develop more sophisticated networks, enhancing the opportunities of individual groups and enabling participation in planning and management at larger scales. (Curtis and Cooke, 2006).

- Many Landcare groups have progressed from focusing on single issues and on small area projects to bigger picture NRM issues and recognise the need to involve the

urban community, local government, rural industry bodies and public land managers as significant stakeholders in NRM ( Department of Agriculture,2003).

### Continuous learning

- The Landcare model provides a sound basis for effective continuous learning. It builds knowledge and understanding that increase participant competency and strengthens capacity for adaptive management processes, as well as providing appropriate institutional structures for ongoing community representation (Curtis and Lockwood, 2000).
- Landcare leaders encourage open-mindedness and an awareness of the diverse reactions to changes throughout the courses of actions when implementing new policies and practices (Catacutan et al., 2009).
- Landcare stimulates continuous learning as a guiding principle and uses champion individuals to deliver capacity building and NRM change through modest resources (Catacutan et al., 2009).

## 3.3 Social – community health and wellbeing

The literature indicates three main pathways in which contact with the natural environment, as one would experience when involved with Landcare and NRM, can improve the health and wellbeing of individuals and communities. These are mapped in Figure 3.

**Figure 3: Logic demonstrating links between Landcare activities and improved health and wellbeing**

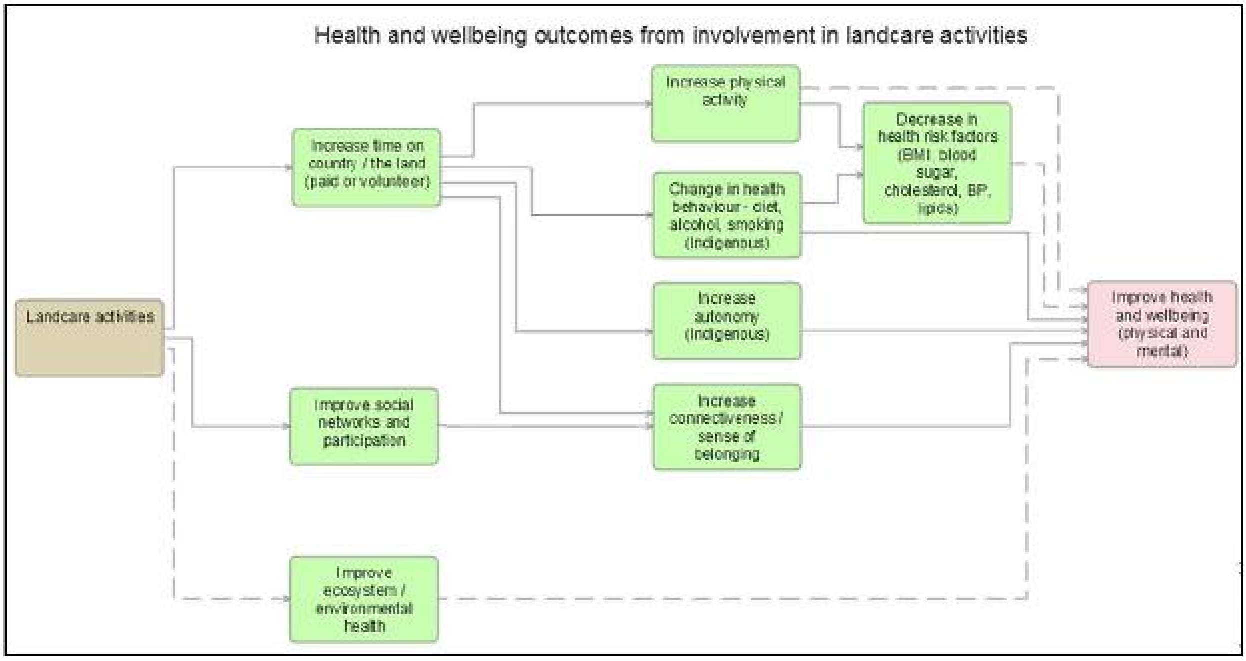


Involvement with NRM or Landcare activities can have positive human health and wellbeing impacts through the improvement of environmental quality or the provision of ecosystem services such as cleaner water, cleaner air quality, improved aesthetics of the

environment, better quality food production and enhancement of environmental services. It has also been argued that Landcare activities that reduce carbon outputs or increase carbon capture or sequestration such as vegetation enhancement activities have a potential global human health benefit.

Other pathways in which improved human health and wellbeing benefits can be derived from Landcare and NRM include improved social networks and participation leading to increased connectedness and sense of belonging, and through increased time on country/land leading to a number of human physiological and mental health benefits. Within this pathway there are a range of specific benefits that have been studied for Indigenous Australians.

The remainder of this section provides an overview of some of the evidence for the existence of these cause-effect pathways (the benefits to Indigenous Australians appear in Section 3.6.

## Contact with a natural environment

- Developed in 1980's, the hypothesis of "biophillia" describes the concept of values of nature whose expression is linked to aspects of physical, emotional, and intellectual growth and development. The hypothesis is based on the idea that people possess "an inherent inclination to affiliate with natural process and diversity, and this affinity continues today to be instrumental in human physical and mental development" (Kellert and Derr, 1998).

- There have been several seminal reviews of evidence relating to the human health benefits of contact with natural environments or green spaces undertaken in the last ten years (Maller et al., 2008, 2002; Townsend and Weerasuriya, 2010). A review was undertaken by Deakin University in 2002 (updated in 2008) which synthesised over 200 items of relevant evidence and concluded that human contact with green nature, such as parks, has a wide range of benefits including reducing crime, fostering psychological wellbeing, enhancing productivity, reducing stress, boosting immunity and promoting healing. The review concludes that the initial evidence for the positive effects of nature on blood pressure, cholesterol, outlook on life and stress reduction provides justification for its incorporation into strategies for the Australian National Health Priority Areas of mental health and cardiovascular disease (Maller et al., 2008).

- A 2003 review on the benefits of contact with nature for mental health and well-being distinguishes health benefits being derived from three different levels of contact with nature: viewing nature, being in the presence of nearby nature and active participation in nature. The latter category includes farming and can be reasonably extended to Landcare and NRM, depending on the specific activity. The review found that physical activity in natural settings greatly improves self-esteem and positive emotions and behaviour and that natural settings promote social exchanges and interactions resulting in positive emotional states and behaviours (Townsend and Weerasuriya, 2010).

## Social networks

- Landcare is based on the interaction of the social aspects of a community and the natural resources that are inherent in the local areas with the understanding that community action is required to meet the significant environmental challenges.

Through this thinking, both the physical environment and the sense of community of participants are improved (Pretty et al., 2007).

- Baum et al (1999) concluded that volunteers were more likely to have more informal social contacts, and to be involved in a range of social activities, than individuals who did not get involved in volunteering. They concluded that the social fabric of a place can be reinforced through the development of social ties created through voluntary work. This is supported by Koss and Kingsley (2010) who state that the notion of volunteer connection to the natural environment and positive mental and emotional health are important for any citizen science monitoring program, such as those delivered by Landcare and NRM.

- The notion of sense of place is important in creating social cohesion in involvement with Landcare activities. Sense of place is not just experienced by people becoming attached to their biophysical surroundings, but can also be seen as extending to emotional attachments to social communities, built through familiarity and spending time in one place. It is believed that spending time in one place and maintaining social contacts can help to build social capital comprising trust, reciprocity, norms, values and networks (Putnam, 1993). It has been suggested that local health centres and general practitioners should encourage senior citizens to become involved in conservation groups in order to increase senior citizens’ level of health and wellbeing and reduce social isolation (Koss and Kingsley, 2010).

## Physical and mental health benefits

- Social epidemiologists have demonstrated how community connections, networks, belonging, social cohesion, and social capital (all central concepts in Landcare and NRM) play a pivotal role in the health, well-being and mental health outcomes of populations (Pretty et al., 2007). Increased social interaction and participation by an individual within a community also enforces a sense of belonging and social connectedness and this has been well linked to positive physical and psychological wellbeing (Cannon, 2008).

- A sense of community provides a buffer against physical and psychological symptoms of illness, and facilitates adjustment (Pretty et al., 2007) and Cattell (2001) states that individuals with many informal networks are less likely to suffer ill health, as these networks provide support, clarify personal identity, enhance self-esteem and enable citizens to feel in control of their lives.

- Social capital is characteristic of “healthy, thriving communities and is strengthened through voluntary activities and organisations” (Gooch, 2003). Koss and Kingsley (2010) studied volunteers in a marine NRM program and found that their involvement in the program made volunteers feel good emotionally and mentally, with active learning, such as remembering names of marine biota, stimulating brain activity and memory and that volunteer monitoring efforts generated personal satisfaction through their contributions, feelings of enjoyment, and socialising with others.

- Burgess and Johnston (2007) report on the preliminary findings of a Healthy Country: Healthy People project where the health benefits of participants in Natural and Cultural Resource Management (NCRM) or Caring for Country versus non-participants was examined. The report found that Indigenous involvement in NCRM is seen as an important determinant of landscape and human health and that

higher levels of participation in Indigenous NCRM may be associated with significantly better health outcomes across a broad array of risk factors linked to diabetes and cardiovascular risk.

- A significant association has been found between greater participation in Caring for Country activities and lower body mass index (Burgess et al., 2008). Similar results have been shown by Garnett and Sithole (2007) who report that participation in Indigenous NCRM was associated with a range of health benefits covering a range of risk factors and disease endpoints. The project findings concluded that the health outcomes associated with Indigenous NCRM can help prevent or delay significant causes of premature disease and death, delivering significant economic savings in health care expenditure (Garnett et al., 2009).

## 3.4 Social – political and social capital

Social capital can be defined as the resources available within communities and networks of mutual support, reciprocity and trust. It refers to the social relationships, networks, norms, and trust within society that help individuals, groups and organisations cooperate for their mutual benefit. Social capital focuses on the capacities of groups of people and their interactions, and is thus distinguished from human capital, which focuses on the capacities of individuals. Social capital is attributable to individuals, groups and communities and is a contributor to community strength (ABS 2004).

Social capital has been an important element in the success of Landcare. The dynamic nature of Landcare has fostered social cohesion within communities, which has, in turn, further enhanced the benefits of Landcare and this social capital has in turn enhanced the Landcare Program (Curtis, 2003). Landcare groups help build social capital by acting through social networks to establish trust and social bonding, and to generate land management norms and standards as well as reciprocal relationships (Beilin and Reichelt, 2010; Cary and Webb, 2000; Youl et al., 2006).

Landcare has built or enhanced social capital that is drawn on in many ways in order to continually enhance the social fabric of rural communities. Landcare contributes to a community's social capital through building relationships, providing new and stronger governance, building resilience, enhancing the benefits of localism, increasing the recognition of women in rural communities and empowering individuals by building self-identity and self-recognition (Webb and Cary, 2005).

A defining feature of Landcare is that its members feel part of a community that provides mutual support, encouragement and reinforcement (Toyne and Farley, 2000) in order to work towards a common goal (Catacutan et al., 2009). This fabric has been instrumental in changing norms about good farming practices, sustainability and land conservation in rural areas (Campbell, 2009; Cary and Webb, 2001; Toyne and Farley, 2000).

A report by Deakin University, commissioned by Parks Victoria, assessed the relationship between humans and natural or green space environments and found that while the relationship between social capital and the biophysical environment is still being explored, it appears likely that human contact with nature through natural parks could have significant capacity for building social capital (Maller et al., 2008, 2002).

The broad social benefits from Landcare are characterised succinctly by Brown, 1997 who states "The results of landcare programs are demonstrable: farmers now walk over each other's farms, once socially unthinkable. City councils team with rural towns, and learn

from one another. Economists, bee-keepers and foresters each have a value for ghost gum (*Eucalyptus papuana*) flowers, a different value that each had not previously appreciated. Women farmers find that they have a voice in local agricultural meetings for the first time" (Brown, 1997).

The following Sections provide a synthesis of evidence of the social – political and social capital benefits resulting from Landcare in the key areas defined by the literature.

## Partnerships and networks

- Landcare has provided the impetus for groups to self-organise into higher level structures or networks. These networks more effectively deliver an extremely diverse range of outcomes, deal better with bureaucracy, have an increased ability to adapt to change, discuss more complex ideas, and are more professional and autonomous (Sobels et al., 2001; Youl et al., 2006; Sobels and Curtis, 2001a).

- Landcare groups and Indigenous communities have many common interests centred on their shared goals for conservation. The Landcare program has been able to provide opportunities for Indigenous and non-Indigenous groups to engage with one another, build relationships, and contribute to improved knowledge, understanding and participation in NRM (Landcare Victoria and Department of Sustainability and Environment, 2008; McTernan and Scully, 2010). Through participation in Landcare groups and these partnerships, Indigenous Australians are also able to learn new skills in environmental management, as well as make contact with decision-making NRM agencies (McTernan and Scully, 2010).

- Landcare has developed new partnerships, strengthened existing friendships and partnerships, and has assisted in breaking down barriers within the community (Curtis, 2003; Curtis et al., 1999; Landcare Victoria and Department of Sustainability and Environment, 2008a; Lockie, 1998).

- The emergence of Landcare networks, involving the organisation of groups, was a development that was largely unforseen but is one of the most substantial achievements of Landcare. Landcare networks have facilitated the emergence of more professional, strategic Landcare planning and action on NRM and other issues. The development of networks has provided new skills in governance, financial management, relationship building and negotiation. These skills have been carried into other areas outside of Landcare (Curtis, 2003, Lockie, 1998).

## Leadership and public participation

- A positive but unexpected outcome from the formation of Landcare groups was the creation of new or expanded leadership and public participation roles in the bush. Many landholders have been able to harness their new organisation to tackle many issues other than those related to NRM, such as declining services in regional Australia. Some Landcare groups have become powerful voices within a framework that sat quite outside the traditional farm organisations and were capable of acting independently from them (Toyne and Farley, 2000).

- These groups have the potential to play a critical role in changing the way that services and funding are delivered to the bush. Many Landcare groups are learning the political benefits of effective local and regional organisation, in pursuit of mutually beneficial goals (Toyne and Farley, 2000).

## Governance and self-regulation

- One of the recognised strengths of Landcare is its diversity, in its members, its geography, its governance and issues. Diversity arises due to localised community-driven voices and because of the inclusiveness of Landcare, which involves the whole community. Landcare has been shown to effectively engage with the young and old, farmers and urban dwellers, 'brown' and 'green'. For these reasons, in addition to the wealth of knowledge and skills held by its volunteers, Landcare is seen as respectable and credible amongst the community and throughout government (Landcare Victoria and Department of Sustainability and Environment, 2008a).
- Colliver (2011) reports that "The dynamic, on-going, self-organised process of learning-by-doing at work in community-based governance finds expression in governance practice that cultivate relationships of mutual responsibility." Griffin NRM and URS Australia Pty Ltd (2001) state that "The outcomes from this large Australian Government supported experiment in community NRM development has created a much better and more sophisticated community-industry-government dynamic in rural Australia that is maturing to the point where it can address the major challenges in NRM."
- Given the governance structures developed and the driving forces such as the motivation of social cohesion and peer support, it is believed that a Landcare movement of some type is likely to persist even without government support (Coree Consulting, 2003).

## Localism and empowerment

- Many members are attracted to Landcare because it embraces local community-based planning and action, because groups are quite independent in determining their priorities and activities and because there has been strong support by government through funding of coordinators, cost-sharing for on-ground work and the development of regional catchment management processes (Curtis, 2003).
- Landcare also creates networks for social support which helps to share the stress of land management issues and rural decline (Campbell, 1995a). Local level discussion and experimentation has been shown to be critical to the development and adoption of sustainable farming practices. Research in Victoria confirms the importance of local landholder confidence in recommended practices as a critical factor affecting adoption of sustainable farming practices (Curtis, 2003).
- Community Landcare groups are also empowered to define their desired outcomes and are actively involved in generating actions for implementation. With this comes responsibility, accountability and the necessary budget that must be held accountable at the community level. It is reported that without ownership, the enthusiasm and commitment of local communities may quickly dissipate. Under a Landcare model, governments provide direct or indirect support without necessarily taking the lead. This trust in community ability at the government level brings out stronger community empowerment (Catacutan et al., 2009).

## Increasing the recognition of women in rural communities

- Landcare is far more inclusive of women than any other farm-based organisation (Lockie, 1998) and has assisted in raising the profile of the role of women in agricultural family business (Hogan and Cumming, 1997).

- Women comprise approximately 30 per cent of all Landcare participants and have taken on roles of leadership that has been a positive experience for most women (Curtis, 2003).

## Personal growth

- Many people in Landcare have learnt a lot about their own properties, about NRM issues in their district and about issues they may have rarely considered in the past (Campbell, 1992). Campbell also states that group leaders in particular have gained much from seeing other people get involved, from influencing others through their interaction in the group and from group projects (Campbell, 1992). Similarly Landcare volunteers acquire self-confidence over time through learning and networking, and the ability and willingness to remain active within the group (Gooch, 2004).

- Landcare group members have remarked on Landcare's ability to promote cooperative discussions and activities surrounding land management practices and problems within the community, thus restoring the "sense of community" that seems to be getting lost in many rural communities (Lockie, 1998).

- It has also been found that a sense of place was a strong motivator for many volunteers, reflecting the need for people to feel connected to their communities. Involvement in Landcare volunteering could provide a counter to contemporary society where many people are increasingly disconnected from places and from nature (Gooch, 2004).

- Another important aspect of personal growth is the concept of identity within volunteer groups. Identity helps to build social communities, ecological identity, and a sense of place, all of which are elements of resilient, sustainable communities.

## Filling the void

- Landcare has also built social capital that has filled a void that has been left through the retraction of social networks due to rural decline and a decline in government services such as agricultural extension services (Webb and Cary, 2005).

- The social capital built by Landcare is a resource that will continue to be drawn on to contribute to achieving NRM outcomes but also other social objectives (Curtis, 2003).

## Increasing awareness, skills and knowledge

- Although this area has been discussed to some extent in Section 3.3, it is important to recognise that these attributes also contribute to social capital. Numerous authors recognise that Landcare has contributed to social capital through increasing

awareness, developing and extending skills and knowledge and developing networks to promote the acceptance of sustainable farming practices (Campbell, 1995b; Cary and Webb, 2001; Curtis and Cooke, 2006; Curtis and De Lacy, 1996; Curtis, 1995; Curtis et al., 1993; Department of Agriculture, 2003; Edmonson, 2010; Griffin NRM and URS Australia Pty Ltd, 2001; Lockie, 1998; Quealy, 1998; Sobels et al., 2001; Toyne and Farley, 2000).

- While Landcare helps to build social capital amongst communities, there is considerable value in the long term influence on behaviour that helps to reinforce more positive behaviour to improve the condition of natural resources (Cary and Webb, 2001, 2000).

## 3.5 Economic

Landcare and NRM have made a long-term, positive impact on the environmental condition, as well as the economic profitability of farming (Curtis, 2003; Sobels et al., 2001). While variations across programs and projects occur, Landcare Victoria and Department of Sustainability and Environment (2008a, 2008b, 2008c, 2008d) found that every dollar that is invested in Landcare leverages 2-5 times that amount through contributions towards labour, equipment, voluntary expertise, and often additional donations from landholders and businesses. The key economic benefits from Landcare and NRM, as identified in the literature are mostly through direct financial returns, increased access to financial resources, and training to improve farming and management techniques.

### Increased financial return

- Cullen et al (2003) prepared a comprehensive review on the economic returns from a set of Landcare practices defined broadly as landcare farming[4]. They found that the effect of landcare farming activities on farm business profit varied widely between farms, meaning it was difficult to estimate the economic return generated by industry-wide adoption of landcare farming.

- However, Cullen et al (2003) note some demonstrated economic returns, including a 3 per cent increase in return to capital and improved soil fertility by effectively integrating landcare farming into an already productive property. They also noted (with some important caveats) significant economic returns including a 640 per cent increase in profit by running merino wethers in a rotational grazing system on native pasture compared with set stocking; a 360 per cent increase in profit by running crossbred sheep under a high-input system compared with a low-input system, a 100 per cent increase in profit by running sheep on perennial pasture compared with annual pasture; and a 21 per cent increase in profit by running merino wethers on fertilised native pasture.

- Cullen et al (2003) also cited a number of anecdotal reports of significant economic returns for individual properties from various aspects of landcare farming. They

[4] Defined broadly to include adopting conservative stocking rates, maintaining or establishing perennial pastures, subdividing land into land classes, practising minimum or reduced tillage, direct drilling, undertaking spring or bare fallow cultivation, tree and shrub planting, regular soil testing, water quality monitoring, pasture monitoring, excluding livestock from degraded areas, placing watering points to minimise degradation, rotating crops to minimise land degradation and retaining stubble to minimise degradation.

report that following significant private sector investment in landcare farming activities, the gross income of farmers in the Woady Yaloak sub-catchment was estimated to be almost 10% higher than farmers in a similar sub-catchment who had not had the same level of investment.

- A case study conducted by Nicholson and Knight (2003) found an increase in gross income from $275/ha in 1990 to approximately $335/ha in 2001 attributed to an increase in commodity prices and property size, but also to improved productivity of the enterprise. The increase in productivity was accredited to a range of factors, including participation in Landcare.

- More recently, Ecker (2011) reported on results of a national survey of Australian farmer's motivations to undertake practice change related to cropping systems, grazing management, native vegetation and control of Weeds of National Significance and found that financial benefits and environmental factors rated highly in influencing land management practice decisions. Financial benefits included increased returns, reduced costs and increasing land value. Increased availability of grazing fodder was also an important financial benefit for both cropping and grazing management practices.

- Overseas results report that economic returns from Landcare in developing counties can be difficult and slow to realise (Metcalfe, 2003), although more recent research is clear about the positive returns to individual framers Newby and Cramb (2011).

## Access to resources

- Landcare networks have become important local organisations through their ability to establish partnerships that help to reduce financial risk (Curtis and Lockwood, 2000). The increased communication and partnerships that emerge from these networks, results in a greater awareness and appreciation for the different values placed on environmental assets by economists, the community and landholders (Brown, 1997). This mutual understanding helps to actively include private businesses in farming, creating more economically sustainable land management systems and improved market share, profits and economic resilience (Catacutan et al., 2009). Investing in Landcare groups is also economically beneficial to corporations through the subsequent association with conservation ethics, which often helps to improve relations with the community (Catacutan et al., 2009).

- Landcare networks have enhanced the ability of community groups to access funds from Government and other organisations (Curtis et al., 1999; Sobels and Curtis, 2001b; Compton et al., 2007). The Department of Agriculture's, Review of the National Landcare Program (2003) discovered that Landcare groups were able to draw down additional funding from non-government parties at a rate of at least $2.60 for every $1.00 spent by the Government on Landcare projects.

- Landcare groups also help reconnect Indigenous Australian's with country (Love, 2012). Altman and Whitehead (2003) and Garnett et al. (2009) found that when Indigenous Australians are connected with country, they are better able to participate in the market sector by utilising natural resources, and to generate income for themselves. Support generated by the Landcare program is therefore able to facilitate sustainable economic development in Indigenous communities while improving disadvantages. This has inherent socio-cultural benefits for

Indigenous Australians and, in addition, promotes spiritual well-being and physical health (Altman and Whitehead, 2003).

### Training and management techniques

- Landcare groups make it possible for a farmer to take greater financial risks with how they manage their farms and this allows them to receive improved outcomes in the long run, financially and environmentally (Campbell, 1992). This is supported by Mues et al. (1998) who found that greater involvement in training associated with Landcare and NRM resulted in larger farm debts initially, but greater physical changes in farm characteristics and higher farm cash incomes over a longer period of time.

- Without training and subsequent implementation of conservation activities and implementation of more up to date sustainable farming techniques, significant economic costs can be associated with land degradation in future generations through the loss of production, reduced biodiversity and loss of environmental resilience (Hamilton, 1995; Mullen, 2001).

## 3.6 Cultural

### Connection with country

There is a strong belief in Indigenous cultures that if an individual does not maintain spiritual, physical, social and mental health they cannot be truly connected with the natural world (Townsend and Weerasuriya, 2010). Further to this, many Aboriginal Australians derive their self-identity from the land (Burgess et al., 2005) and traditional lands offer an outlet to reduce stress from daily pressures, being described somewhat like a utopian sanctuary (Kingsley et al., 2009).

There is also a growing recognition that Indigenous community-based involvement in NRM can bring significant economic and socio-cultural benefits (Altman and Whitehead, 2003). The socio-cultural benefits for Indigenous Australians engagement in NRM activities on country include being remote from access to potential negative influences and more positively living a lifestyle that promotes spiritual and physical well-being (Altman and Whitehead, 2003). Other evidence reviews, have concluded that engagement of Indigenous Australians with land management can enable people to feel that their actions are consistent with their own sense of the right and proper way for them to behave towards land, family and community (Davies et al., 2011). Similarly Johnson (2007) remarks on Indigenous people who have described the relief and rejuvenation of returning to country even if for brief visits, to burn country, to hunt and gather food, to educate young people and to maintain cultural and spiritual obligations (Johnston et al., 2007).

For many Indigenous Australians, NRM is caring for country as it "embodies deep spiritual obligations and patterns of behaviour proscribed by enduring metaphysical associations with geography" (Burgess et al., 2005). This is defined as Indigenous participation in interrelated activities with the objective of promoting ecological and human health (Burgess et al., 2008). Burgess et al (2009) also adds that caring for country is a "community driven movement towards long-term social, cultural, physical, and sustainable economic development in rural and remote locations, simultaneously contributing to the conservation of globally valued environmental and cultural assets."

Ganesharajah (2009) extends these outcomes across into areas covering equity and empowerment noting that Indigenous Australians engaged in valued services, such as Indigenous Natural and Cultural Resource Management, can use their service delivery as a type of bargaining tool or a form of leverage and that this can at least go some way towards reducing Indigenous peoples' vulnerability to power inequalities and also to increasing autonomy which is an important determinant of health.

# 3.7 Resilience

As noted above, the multiple benefit categories from Landcare and NRM are linked and interdependent. This is evident across a number of categories and sub-categories, but is particularly the case in relation to resilience principles and their inherent relationship (at least in an NRM context) to linked socio-ecological systems. In general terms, the literature review found that engaging with Landcare and delivering NRM outcomes assists participating communities to build the resilience and adaptive capacity of their social-ecological systems (Buikstra et al., 2010). The interdependence of Landcare, NRM and socio-ecological systems is evident in much of the literature, as summarised below.

Landcare promotes the formation of networks that allow communities to support each other, thereby increasing social cohesion through incorporating social, economic, environmental and cultural considerations into Landcare activities (Colliver, 2011; Landcare Victoria and Department of Sustainability and Environment, 2008a; Love, 2012). This has been accomplished through the ability of a Landcare group to involve a range of landholders and community members in order to deliver project outcomes, market-based instruments, integrate actions with NRM priorities, and engage with corporate companies in to address system-wide issues that contribute to resilience such as "climate change, sustainable farming, biodiversity loss and urban growth" and this will ultimately create stronger, healthier communities that are better prepared to cope with change (Landcare Victoria and Department of Sustainability and Environment, 2008).

A participatory study found that environmental factors such as connection to the land, and a strong sense of community, influence the overall resilience of the individual, which subsequently contributes to the resilience of the community and the entire socio-ecological system (Hegney et al., 2008). The eleven major concepts that (Hegney et al., 2008) considered necessary to enhance resilience were: social networks and support; positive outlook; learning; early experience; environment and lifestyle; infrastructure and support services; sense of purpose; diverse and innovative economy; embracing differences and beliefs; and leadership.

In another study, 72 participants from six different sectors were interviewed to identify and explore what makes up community and individual resilience. All sectors identified the presence of social networks and support (such as that provided by Landcare) as a critical resilience factor and a key element of community resilience and an ideal resilient community (Buikstra et al., 2010).

The 2008 Victorian Landcare Forum reported that participation in and the philosophy of Landcare can lead to innovation and the adoption of new technologies in order to increase production and enhance the sustainability of our actions (Landcare Victoria and Department of Sustainability and Environment, 2008). Therefore, increasing the skills base of Landcare farmers assists with adaptability and resilience of the socio-ecological system.

Community based Landcare is a very successful NRM platform through its ability to help rural communities engage with their socio-ecological system and enhance community-wide learning, and resilience (Beilin and Reichelt, 2010). By enhancing socio-ecological resilience, Landcare has also been able to support adaptive management when stakeholder engagement is not sufficient (Curtis et al., 2000).

# 4. Multiple benefits of Landcare and NRM: The practice

A key component of the project was an assessment of the practice of Landcare and NRM in delivering multiple benefits. As outlined in Section 1.3.2, this was achieved through a series of interviews and case studies.

The results of the interviews and case studies are presented below in a manner similar to the findings of the literature review. (In keeping with the approach of the interviews and case studies, the results are presented a little less formally). This section also follows the approach of the literature review in that it is focussed on reporting results with discussion deferred to the synthesis component of the report (Section 5).

## 4.1 Summary of interviews

### Learning, awareness and practice change: Landcare and NRM deliver a range of outcomes that are built into formal or informal continual improvement processes

Interviewees tended to focus on broad community benefits which encompassed the interdependence of shared ideas, attitudes, ongoing beliefs and capacity building in what can be seen as an informal continual improvement process. A range of ongoing benefits and flow on effects were reported from initial Landcare activities, some of which linked closely to increasing social capital and community leadership and others were more clearly focussed on training, skills and capacity building (e.g. ongoing use of demonstration sites).

There were also a range of comments relating to the involvement of schools which were represented as improved attitudes and linkages to further NRM-based education. Getting the Landcare and NRM message into primary schools through Junior Landcare was seen as an important starting point in building environmental and social capital within communities.

### Social – community health and wellbeing: An under recognised part of the big picture of Landcare and NRM

Interviewees strongly highlighted the important role that Landcare has played in establishing and activating social networks and in some cases replacing networks that were poorly supported or no longer relevant. People come together for a common purpose which in turn delivers outcomes that exceed the original purpose. This includes meeting for various topics, such as mental health, drought management, Indigenous heritage and local community events.

Increasing contact with the natural environment was also mentioned, and this was also placed within the context of social integration locally and between the city and the country. This does not need to be a formal approach with one example cited where city people become connected with coastal communities when visiting holiday homes (through activities such as "Summer by the Sea", an educational program which runs during the summer holidays).

## Social – political and social capital: Shared ideas, attitudes and capacity building deliver a range of flow on effects

Participation in networks was seen as important, as was the interlocking network of relationships between groups and individuals. The Landcare Facilitator was seen as providing an important linkage to big picture policy level issues, being able to apply these to a local level and feeding issues back to government. Some interviewees noted this was very important for community engagement on difficult issues such as climate change.

A cohesive voice on behalf of the community Landcare movement was also seen as important as this increases the community involvement in government decision-making at a range of levels, from regional NRM groups (CMAs and their equivalent) to local, state and Australian Government and elected representatives.

The recent Health of the Landcare Movement survey by the National Landcare Facilitator was cited as measuring the attitudes of farmers and groups. The survey indicated that over 60% of respondents believe Landcare plays a role in increasing social capital.

Landcare and NRM co-ordinators as individuals contribute to social capital by bringing professional skills to a local community (such as the ability to obtain funding, knowledge of government structures, facilitation skills) and deliver upstream outcomes to their organisations (NRM groups and Landcare Networks).

## Economic: There is a multiplier effect of every dollar invested in Landcare and NRM through volunteering, land management practices and investment in natural and human capital

Interview respondents highlighted that in-kind contributions substantially enhanced the value of program outcomes. In general terms interviewees were supportive of the notion that investing in Landcare and NRM provided value for public money invested (e.g. multiplier effects of enhanced aesthetics providing flow on effects such as tourism).

It was also noted that broader economic benefits accrued from investment in Landcare and NRM. These extended beyond just physical infrastructure (such as fencing off creeks) to wide ranging economic outcomes benefitting the wider community such as enhanced natural capital, human capital and local identity. Benefits from land and water improvement activities can include quantifiable economic benefits. Similarly, the adoption of minimum-tillage and other practices to increase soil carbon were reported to have benefits to landholders' bottom line and the wider community (e.g. erosion mitigation and food security).

## Cultural: Landcare and NRM deliver engagement and integration of individuals into the community through connection to country

A wide range of cultural multiple benefits were reported through interviews. Aboriginal Australians were seen as a key focus in terms of preserving and accessing traditional customs, knowledge, language and medicines. Connection to country using language as an indicator was also mentioned with one interviewee noting this can be measured by the use of language, number of speakers, signs and documentation, names of children and places and providing a reference to Cullen-Unsworth (2011).

Using landcare to engage migrants into a new community was also mentioned as providing an immediate connection into the local landscape.

### Resilience: Landcare and NRM build the capacity of a community to respond to environmental and other disasters

Interviewees were clear about Landcare and NRM building community resilience, but less so on ecological resilience. Some literature was mentioned, for example attributes of social resilience encompassing knowledge, capacity and aspirations, governance, economic viability and community (Dale et al, 2011).

Others mentioned research (Ross et al, 2010) on the general conditions that communities require to be resilient, which include:

- people-place connections
- knowledge, skills and learning
- community networks
- engaged governance
- a diverse and innovative economy
- community infrastructure

In times of drought and other hardships, it was reported that Landcare groups often switch their focus to more social/health related topics and serve as a vehicle to meet these needs, often because no other providers exist.

### 4.1.1 Measuring Multiple Benefits – ideas from interviewees

Interviewees offered a range of perspectives about measuring multiple benefits, as well as sample indicators and methods. These are summarised at a high level and, consistent with the overall approach to this Section; appear more or less as they were suggested to GHD.

**Table 3: Summary of ideas to measure multiple benefits from interview process**

| Suggested approaches | Suggested indicators |
|---|---|
| Most significant change model.<br>The three P's of climate change and agriculture – the physical, the peripheral and the policy.<br>A think tank/discussion group asking heads of communities "what was lacking" (e.g. social capital, infrastructure).<br>Change in Knowledge, Attitude, Skills, Aspiration and Practice (KASAP).<br>Monitor the growth of Landcare groups and group activities.<br>Benchmarking (e.g. profitability)<br>Progressive farmer model.<br>Human development model.<br>Social research tools for measuring the level of social capital.<br>Resilience model (social capital at core – can be measured qualitatively or quantitatively). | How many people are engaged in Landcare and NRM?<br>How long Landcare groups exist (tests resilience and social capital).<br>Increase in the number of Landcare groups.<br>Increase in number of Landcare group members.<br>Degree of self-governance.<br>Schools involved in Landcare.<br>Survey individuals (e.g. "in the last 10 years, what has changed?") to gauge success.<br>Ascertain the extent to which groups share resources.<br>Assess if learning (co-learning or field work) is implemented.<br>Assess the quality of participation in Landcare and NRM. |

## 4.2 Case studies

Information on the process for selecting case studies and the case study approach is detailed in Section 1.3.2. The template used for case study investigation is included at Appendix E along with additional findings from each case study (case study photographs, background material etc).

### 4.2.1 Summary of case study findings

**Learning, awareness and practice change: Landcare and NRM are filling part of the gap from the drop in formal extension services and are highly responsive**

The case studies reported strong and consistent themes covering the learning, awareness and practice change benefits of Landcare and NRM for group members and the ability for these benefits to operate at a range of scales. Some evidence was also provided covering the reach of Landcare and NRM into areas beyond the membership of the typical Landcare group or network. There were frequent comments made in case study interviews that Landcare and NRM had picked up the extension effort that was no longer being delivered by state and territory departments and/or that the extension delivered by Landcare was more responsive and relevant.

Continuous learning cycles were frequently reported and these were seen to be responsive and readily adapted to fit the needs of each Landcare Group. Training courses were changed as groups moved into new and emerging NRM areas such as the Carbon

Farming Initiative/carbon farming and in response to shocks (e.g. customised courses dealing with various issues relating to bushfire recovery).

Outside of Landcare groups, the most frequently reported learning and practice change benefit was in community awareness of key NRM issues, multi-generational reach and practical ways in which the broader community are able to deliver NRM improvement.

**Social – community health and wellbeing: Significant contact with the natural environment is present, but benefits are not well recognised**

Few of the case study participants explicitly commented on multiple benefits relating to social – community health and wellbeing. However, multiple benefits in these areas do occur and data relating some of the sub-categories of multiple benefits is easily obtained. For example, all of the case studies reported some amount of increased contact with the natural environment, the most prominent example being the Fire Recovery Project which reported 6,267 community volunteer days contributed to a large integrated project. A large number of these volunteer days were for community members not previously involved in Landcare and involved those who had little contact with the natural environment (e.g. volunteers from urban areas).

Many of the case studies reported that their projects/groups developed significant social networks as a result of Landcare and NRM, but again those involved in the case studies did not tend to see a strong link with these networks contributing to broader community health and wellbeing.

**Social – political and social capital: Significant and well recognised multiple benefits exist**

All of the case studies demonstrated significant outcomes in relation to the social – political and social capital multiple benefits. Partnerships and networks were a common theme and these extended across a diverse range of areas including schools, religious organisations, other Landcare groups, regional NRM groups, local, state and Australian Government, industry groups, community-based clubs and societies, Indigenous groups and conservation organisations. These networks provided significant social capital and linkage across and between scales which would otherwise not have existed.

The leadership and public voice of Landcare and NRM was a common feature of the case studies. This functioned at two main levels: firstly where Landcare and NRM groups had advocated on a broad range of issues of concern and secondly where Landcare group members had progressed from membership of a local Landcare group to other local, state and national level roles including local councils, boards, community organisations and other Landcare and NRM organisations.

Some of the case studies had increased the recognition of women in rural communities. This was a significant component of the Naturally Resourceful program where participants reported a profound change in the way women were able to successfully operate in their local communities. The Roper River Mangarrayi Rangers Project case study was also working to increase the role of Indigenous women by expanding the program to include women Rangers who would include women's cultural sites, women's ceremonies and broader capacity building elements into the Project.

The case studies also reported strong outcomes in the other sub-categories of this multiple benefit. As examples:

- Landcare and NRM groups were filling the void by providing services in areas formerly provided by government. This includes in recreation, learning and practice change and extension and disaster recovery. The latter was seen as especially important in the Fire Recovery Project with the model subsequently adapted to function in other areas, including after the 2010/11 Queensland floods.
- Related to the sub-category of leadership and public participation, localism and empowerment was also important in some case studies where Landcare groups in particular had lobbied on issues of concern that would otherwise have had little traction.
- A number of case studies commented that the structure, funding and project delivery provided by Landcare and NRM had built capacity and delivery in a range of areas covering governance and self-regulation.

**Economic: Landcare and NRM generate significant economic returns, but it is seldom a motivating factor at group or organisational level**

While the literature reports some significant economic returns from Landcare and NRM at individual property level and some amount of regional aggregation, the case studies are not especially strong in this area. This seems to be mostly because the case study projects that were investigated have been created to generate an environmental rather than an economic return, or that they have been conceived to deliberately deliver triple bottom line outcomes.

Direct economic returns are present in all of the case studies, but seem to be more important to groups and communities that are struggling socially or that have suffered a significant shock:

- The Roper River Mangarrayi Rangers Project has provided jobs and attracted significant funding delivering economic activity in an area of high unemployment
- The fire recovery project generated considerable sums for investment in communities that were devastated by the Victorian bushfires, at a time when other community and government investment was limited

All of the case studies had accessed government or corporate support for their region and/or Landcare group, which they believe would otherwise not have been invested. This sometimes amounted to millions of dollars, but there is no information available as to the ultimate regional impact of this investment.

**Cultural: Landcare and NRM deliver genuine connection to country**

The main cultural benefits reported in the case studies related to improved connection to Country for Indigenous communities. This was demonstrated across urban (Narrabeen Lagoon in Sydney) and remote areas (Roper River in the Northern Territory) and contributed towards European understanding of traditional Landcare and NRM activity and to the preservation of and access to traditional knowledge.

**Resilience: Landcare and NRM make many small interventions that collectively build resilience**

The case studies demonstrated a series of areas where Landcare and NRM had built resilience. While it came after a significant shock, the fire recovery project was viewed as

a major morale boosting exercise when little was available and it contributed to the ability of the local community to bounce back from a major bushfire. The ability of the community to apply the same approach in future and apply it to other situations such as a flood will also improve resilience. The Roper River Mangarrayi Rangers Project has also improved community resilience through its contribution to individual and community pride, caring for country, an improved sense of connection to place and less social issues.

### 4.2.2 Case Study 1: Upper Goulburn Landcare Network and Goulburn Broken CMA – Fire Recovery Project

**Upper Goulburn Landcare Network**

The Upper Goulburn Landcare Network (UGLN) is a collective of 14 Landcare and land management groups in the Murrindindi Shire, North-East Victoria. The UGLN has been operating for over 10 years and has a strong and positive relationship with landholders, community organisations and government and agency stakeholders.

The UGLN's role is broad, including:

- Landcare group support
- communication of Landcare and land management information
- community capacity building
- sustainable, community-driven land management
- community education and project management
- liaison with government and agency stakeholders
- contribution to catchment NRM targets.

**Background**

Since the Victorian bushfires of 2009, which resulted in 173 deaths (mainly around the Kinglake and Marysville regions), the UGLN has been working with landholders and local communities in the Murrindindi and Mitchell Shires to rebuild and rehabilitate the natural environment and assist the local community recover from the tragedy through a series of programs.

Landcare responded in the aftermath of the bushfires by establishing an emergency relief fund. Raising over $1m in contributions from corporate Australia, Landcare and the Department of Sustainability and Environment in Victoria worked together to deliver funding to fire affected regions to achieve community and environmental recovery from bushfire (Landcare Online, 2012). Additionally, the Australian Government's Caring for our Country program funded fire recovery work in the Goulburn Broken Catchment.

The Fire Recovery Project was delivered through the Goulburn Broken Catchment Management Authority (GBCMA) and UGLN. The project encompassed the following programs:

- Fencers Without Boundaries

- Operation Coughing Parrot
- The Lorax Project
- Fauna Surveys in Remnant Vegetation
- Weed ID and ACUP Training.

The key aims of the project were erosion control, weed control, remnant vegetation protection, installing wildlife nest boxes and volunteer assistance coordination.

**Table 4: Multiple benefits of the Fire Recovery Project**

| Multiple benefit | Description |
|---|---|
| Learning, awareness and practice change | Multigenerational reach – The network engaged youth (such as colleges at Benalla and Ballarat and Rotary South Australia) in the process for various tasks (such as construction of the nest boxes) which created a knowledge transfer, increased awareness and the knowledge of bushfires.<br>Scale of change – Outcomes included a progression of programs, the reinstatement of assets of environmental value, fencing (the opportunity for fencing to be undertaken by land class to improve productivity) and an opportunity for improved run off (erosion) control.<br>Scale of change – The creation of new groups has occurred as a result of the fires.<br>Continuous learning – Improved knowledge of landscape rehabilitation through the Focus on Fauna Program, a program focussing on how wildlife fits into the environment. The program engaged members and non-members with over 70 participants involved.<br>Awareness raising – Landcare was on the ground, had the ability to react using its own resources. This included a Help is at Hand form; people were able to nominate problems (such as environmental issues).<br>Continuous learning – Training courses such as a weed control workshop, farm chemicals users' course, 1080 baiting course and habitat management course had high participation levels, which were critical for the recovery process but also provide ongoing production benefits. As of September 2012, attendance across 19 training courses totalled 327. |

| Multiple benefit | Description |
|---|---|
| Social – community health and wellbeing | Contact with natural environment – The network engaged youth (such as colleges at Benalla and Ballarat and Rotary South Australia) in various tasks and delivered a total of 6,267 volunteer days many of which included contact with the natural environment.<br>Social networks – Occurred both within the community and from outside the community, such as from the Toyota 4WD Club, a self-sufficient group who provided labour in the recovery process.<br>Social networks – Reciprocal visits occur between Landcare groups to view each other’s projects and take away learning’s from each other. |
| Social – political and social capital | Partnerships and networks – Occurred with school groups, corporate volunteers (e.g. NAB, Ford, and accounting firms), church volunteer units (e.g. Uniting), Rotary, individuals and other Landcare groups.<br>Partnerships and networks – Engaged different groups, including activities such as New Tree Days where people from all ends of the network come together (from the 11 groups in the network) and cricket matches (such as Yellow Creek/Dairy Creek vs. Strath Creek).<br>Leadership and public participation – There has been a progression of an individual from the Chair of Landcare, to Chair of the Network, to Chair of Victoria Landcare Council to the Australian Landcare Network.<br>Leadership and public participation – The delegate on the Victoria Landcare Council lobbied for the reintroduction of coordinators because of the changes which occurred with the establishment of Caring for our Country.<br>Government and self-regulation – The network is incorporated so it is able to receive government funding and holds its own AGM. The network is accountable, with a voluntary executive group that is governed and self-regulated to a high standard.<br>Localism and empowerment – There is the opportunity for people to join the group and move up the ladder through the organisation and on to greater heights. It has given the community confidence and other networks have been established.<br>Personal growth – There has been a community recovery committee in place which has provided an avenue for personal growth following a traumatic event.<br>Increasing the recognition of women in rural communities – The group is split evenly between males and females, with females well represented in the executive.<br>Filling the void – The network provided a model from which Queensland flood recovery organisers sought advice. The CMA enshrined a strategic model for rolling out future environmental recoveries. |

| Multiple benefit | Description |
|---|---|
| Economic | The fire recovery fencing program Fencers Without Boundaries contributed tens of thousands of dollars to the local community and small businesses by sourcing local accommodation, catering and meals, transport, machinery, safety wear suppliers, timber and hardware.<br>The steel recycling program, Recycling for Recovery, raised a significant sum of money through a Victorian and Federal Government-funded clean-up of properties destroyed in the 2009 bushfires, carried out by Grocon on behalf of the Government.<br>"Without the UGLN and GBCMA facilitators the project wouldn't have started. They were critical in initiating and driving the program" (UGLN President).<br>A total of 6,267 volunteer days were contributed to the fire recovery project, contributing a significant human capital resource. Investment from corporate partners was invaluable, with many, such as banks, contributing entire teams for days at a time to undertake well managed tasks. Examples include the access to equipment, machinery and personal, fire fighting vehicles and volunteers.<br>Full time employment also emerged from the volunteer sector. For example, a farmer who now suffers from ill health due to smoke inhalation following the bushfires now employs someone who was originally a volunteer – an opportunity that would not have arisen had the volunteer network not been in place.<br>Seed orchards and farm forestry plantings were encouraged as part of the revegetation process which has the potential to deliver future economic benefits. Emphasis was also placed on the aesthetic value of plantings. |
| Resilience and health | The program was viewed as a morale boosting exercise – "The program and its results impacted on peoples morale, sometimes simply by having other people turn up at their place to help" (UGLN President). Labour was available that would not otherwise have been if not for the volunteer presence. |

**Partners**

UGLN engage with the following partners during the Fire Recovery Project:

- Department of Primary Industries, Victoria
- Department Sustainability and Environment, Victoria
- Local Government (Murrindindi Shire Council)
- Goulburn Broken CMA
- Federal Government - Caring For Our Country
- Charted Practicing Accountants

- Uniting Church
- United Care
- Greening Australia
- Men's Shed (Shepparton)
- Rotary
- Scouts
- Kevin Hienze School of Horticulture, Euroa Native Plants Arboretum
- Numerous corporate organisations (e.g. Grocon, NAB, ANZ, JBWere, Ernst and Young).

**Further information**

For additional information see http://landcarefirerecovery.blogspot.com.au/ or http://goulburnbroken.landcarevic.net.au/ugln

### 4.2.3 Case Study 2: Otway Agroforestry Network – A Web of Trees: Yan Yan Gurt Catchment

**Otway Agroforestry Network (part of the Upper Barwon Landcare Network)**

The Otway Agroforestry Network (OAN) began in 1993 as a federally supported farm forestry project managed by a group of landholders from two large Landcare groups. OAN has become a leader in assisting farmers integrate trees into their farming landscapes for both conservation and profit. The group's approach encourages landholders to identify opportunities and design forests that slot neatly into their faming, family and community environment. The diversity of activities that are now being undertaken across the landscape reflects the physical, social and economic diversity inherent within the community (source: OAN).

**Background**

Between 1990 and 2002 forest cover in the Yan Yan Gurt Catchment increased from 6% to 21% in total area. More than 20 families have planted trees on cleared farmland with at least 10 managing their trees for sawlog production. Tree plantings are owned by farmers and dispersed through the farming landscape in a manner acceptable to the rural landscape and the community.

In addition to the scale of revegetation that has taken place, a key feature of the catchment is the diversity of aspirations and interests amongst the landholders, industry stakeholders and their supporters.

Family and farm forestry makes a significant contribution to sustainable wood supply and also ensures that commercial tree growing has local community support, underpins sustainable agricultural production and delivers real environmental benefits.

The project aims to achieve environmental outcomes (protection from wind erosion, habitat corridors, soil, water and air quality improvements, animal corridors and assistance with selenium deficiencies by bringing nutrients up from low depths) and economic outcomes (wood production, increased land values).

**Table 5: Multiple benefits of a Web of Trees**

| Multiple benefit | Description |
|---|---|
| Learning, awareness and practice change | Continuous learning (training and management techniques) – Multiple training activities have been undertaken to demonstrate the benefits of tree rows and agroforestry to other Landcare groups including foreign delegations.<br>Continuous learning (research and demonstration) – The project has been used in various studies to demonstrate new techniques such a presentation at the Australian National Vegetation Conference (VegFutures, 2008).<br>Practice change – Changed approach by landowners to land management, not just though tree row planting but the benefits of changing other aspects to reap productivity gains.<br>Multigenerational reach – The OAN has a peer group mentoring program which facilitates generational learning to assist other farmers identify, design and implement a multipurpose tree growing project on their farms.<br>Continuous learning (risk management) – The group gained an understanding of the biological benefits and integrated pest management benefits. Additionally, the risk of fire caused by wind (which occurred in the Ash Wednesday fires of 1983) enhanced risk management techniques. |
| Social capital | Partnerships and networks (guest speakers) – The profile of OAN has allowed the Network to attract guest speakers from across Australia and the world. This allows a sharing of ideas and the building of social capital. |
| Economic | Access to resources (funding) – The group has been able to obtain funding through various programs linked to NRM.<br>Access to resources (amenities) – The Web of Trees project provided increased aesthetics and improved the natural resource and productive capacity (i.e. agroforestry) of the land, increasing the land value of the properties which were involved in the project.<br>Investment multipliers (agriculture and timber production) – Improvements in agricultural productivity were achieved on the understanding that increased that tree cover enhances animal and pasture productivity. Timber production is through pulpwood plantations owned by private landholders.<br>Access to resources – Trial sites and field days demonstrate a commercially viable practice. |

**Partners**

- Master Tree Growers Australia
- Central Victorian Farm Plantations Inc.
- Corangamite CMA

### 4.2.4 Case Study 3: Queensland Murray Darling Committee and Mitchell Landcare – Naturally Resourceful program

**Queensland Murray Darling Committee and Mitchell and District Landcare**

Queensland Murray Darling Committee (QMDC) offers training programs to communities interested in NRM. Topics range from the technical aspects of NRM such as weed, water, salinity, vegetation and pasture management, to leadership and business skills. The QMDC flagship programs are the Naturally Resourceful and NRM Champions courses.

Mitchell and District Landcare has been operating since 1990 and is a network of six sub-catchment groups located across the Mitchell and District area. These groups effectively link the whole catchment directly adjacent to the Maranoa River. The Landcare group is well recognised and supported by the local community for maintaining a balance between better NRM practices and the financial and production needs of the mainly grazing businesses of the region. The group has been successful in managing a wide range of projects, bringing significant financial activity to a small rural town and supporting critical non NRM needs of the community.

In November 2012 the Natural Resourceful Summit was hosted by Mitchell and District Landcare, which contributed to providing a much needed economic injection into a community which suffered setbacks from the severe effects of flooding in 2012.

**Background**

The Naturally Resourceful program is a short NRM leadership course specifically designed for women, developed by QMDC in 2006. The program originated in the QMDC region and attendance has since expanded to over 340 rural women at various locations across Queensland (see Figure 4).

**Figure 4: Naturally Resourceful Workshop locations**

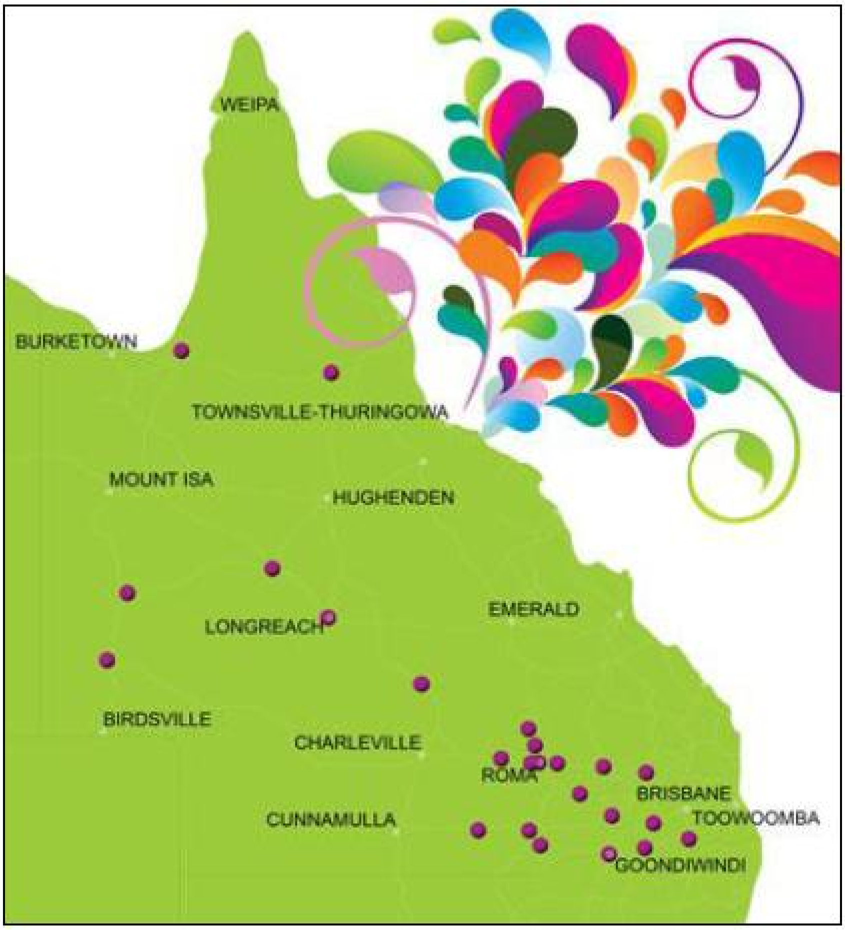


The program was developed in response to a desire by women in Landcare groups in southern Queensland to be more involved in decision making. The workshops recognise that rural and regional women are playing an increasing role in driving the future of their local communities, industries and natural resources – through volunteering, leadership and mentoring. Naturally Resourceful promotes long-term attitudinal change and increased involvement in the family business and the broader community.

The Naturally Resourceful workshops build on the resourceful nature of rural and regional women. The program:

- enhances the involvement of rural and regional women in natural resource and agricultural management
- helps women to build the skills, knowledge and understanding of natural resource management and property planning to give them confidence to fully participate in both business and community activities
- builds the capacity of rural communities and enterprises to respond to an increasingly variable climate and changing consumer demands while delivering sustainability for both the environment and business

- provides the opportunity to reach an ever-wider audience to encourage the consideration of the triple bottom line (people, planet and profit) in all aspects of business and community planning.

**Table 6: Multiple Benefits of the Naturally Resourceful program**

| Multiple benefit | Description |
|---|---|
| Learning, awareness and practice change | Multigenerational reach – The program allows for the sharing of ideas and knowledge across ages and demographics.<br>Scales of change – The program was originally developed for the southern Queensland region, but has since expanded to cover many areas of the state, extending as far north as Weipa.<br>Improved knowledge, continuous learning and practice change – Participation in the program allows for the development of techniques, ideas and learning's by participants and exposure to expertise in areas such as leadership, the Carbon Farming Initiative and the arts. |
| Social – political and social capital | Increasing the recognition of women in rural communities – The Naturally Resourceful workshops have often had a profound impact on the way that women operate in their local communities.<br>Leadership and public participation – The workshops have been catalysts to representational opportunities for graduates on local boards, councils and a range of community organisations. Groups such as the Injune group have been operating as an extended network for social, business and community activities since their workshops in 2007, while the Moonie group has maintained social involvement such as regular yoga classes since their workshops in 2008. |
| Economic | Access to resources (peer support) – Women from isolated communities, such as Eromanga, have been supported after recognition of stress related issues following Naturally Resourceful workshop attendance.<br>Access to resources (community benefits from workshops and forums) – Holding events such as the Natural Resourceful Summit in Mitchell after a significant disaster provided an economic boost for the local community through accommodation and localised spending.<br>Improved financial management – The program has components which focus on topical financial issues within rural communities, such as the Proactive succession planning and financing options session at the Natural Resourceful Summit. |

**Partners**

QMDC have worked with local Landcare groups to develop and organised the workshop program. Key groups have been the Waggamba, Condamine Headwaters, Murilla, Mitchell, Millmerran, Maranoa Regional, Tara, Warroo/Balonne, Inglewood and Brigalow/Jimbour Floodplain Landcare groups. Desert Channels Queensland and Northern Gulf NRM groups have sponsored Naturally Resourceful workshops in their regions, while

the Border Rivers and Maranoa/Balonne Catchment Management Associations, Powerlink and a range of commercial sponsors have supported workshops in southern Queensland.

**Further information:**

For additional information, see http://www.qmdc.org.au/

### 4.2.5 Case Study 4: Friends of Narrabeen Lagoon – Creating a Sustainable Catchment program

**Friends of Narrabeen Lagoon**

Friends of Narrabeen Lagoon are a group of concerned individuals who live either locally or within the northern Sydney region of NSW who wish to see the Narrabeen Lagoon catchment properly and sustainably protected for the benefit of current and future generations, as well as the rights and needs of the ecosystems within the catchment.

Members of Friends of Narrabeen Lagoon have been involved in community actions to protect areas with natural and cultural significance. They have significant links within the Warringah community and elsewhere and aim to inform and educate others about the importance of properly protecting and managing the Catchment.

**Background**

The Narrabeen Lagoon – Creating a Sustainable Catchment program implemented by Warringah and Pittwater Councils, as joint managers of the lagoon, along with activities undertaken by Friends of Narrabeen Lagoon have specific recreational and health benefits associated with their activities. The Friends of Narrabeen Lagoon Catchment aim, through advocacy and education:

- To preserve the Narrabeen Lagoon catchment, its bushland, creeks, flora, fauna and cultural sites and to seek to reduce pollution and degradation
- To help preserve, restore and improve bushland and wildlife corridors connecting the catchment to Garigal National Park and other nearby bushland
- To gather scientific and other information relating to the past and present condition of the Narrabeen Lagoon Catchment
- To support fund raising to achieve a sustainable natural environment in the region
- To support the identification of all Aboriginal, cultural, ecological, historical and recreational artefacts, places, rock carvings, trails and other items consistent with the natural and social values of the catchment
- To use this information to consult and co-operate with all relevant authorities in all matters affecting the welfare and beauty of the catchment system and environs
- To raise a greater public awareness of the need to maintain the lagoon and bushland in a healthy state
- To communicate with and seek affiliation with other environmental protection organisations

- To seek rehabilitation and regeneration work in the Narrabeen Lagoon Catchment.

The following section outlines the key program areas undertaken by Friends of Narrabeen Lagoon.

**Recreational activities**

Friends of Narrabeen Lagoon run various events and activities which are run by volunteers and open to members of the group and the general public. The following activities are held at various intervals and provide recreational and social benefits to the broader community:

- Ecopaddle
- Bushwalk
- Bicycle ride
- Tag Along Tour.

**Forums**

Friends of Narrabeen Lagoon have evolved to become an example of leadership and public participation. The group often hosts forums on various topics, such as the following (in order of occurrence):

- Urban Sustainability Grant program
- Balancing Development and Environment – Community Consultation in the Planning Process
- Meeting the Challenge – How do schools, business and community live sustainably in a catchment?
- Bushfire threat – Protecting your patch
- Threats from the Sea
- Transport – Past, present and future
- Protecting our catchment
- Aboriginal Heritage Forum
- Recreation on Narrabeen Lagoon and in the catchment
- Focus on Fish
- Meet the Candidates (members of Friends of Narrabeen Lagoon who are standing for election in Warringah or Pittwater Council areas).

**Protection of bushlands**

Friends of Narrabeen Lagoon have engaged with Members of Parliament, challenged and informed Crown Land assessments, documented historical events and obtained Heritage Listing (as an "indicative place") for Narrabeen Lagoon Catchment.

**Table 7: Multiple benefits of creating a Sustainable Catchment program**

| Multiple benefit | Description |
|---|---|
| Learning, awareness and practice change | Scales of change – The Friends of Narrabeen Lagoon, through the protection of bushlands and forums, engaged politicians and government agencies on various issues which arise, resulting in significant change (including legislative). Multiple group members have moved into leadership positions within the broader Sydney Northern Beaches community.<br>Awareness raising – Recreational activities raise the awareness of the general public to environmental issues and the need for protection of the lagoon.<br>Improved knowledge – Various activities, such as forums and recreational activities improve the knowledge of the members, wider community and policy makers. |
| Social – political and social capital | Partnerships and networks – Linkages with other care groups, local government (Warringah and Pittwater) and school and community organisations.<br>Filling the void – The group performs recreational activities and the protection of bushland that may not otherwise exist.<br>Leadership and public participation – Members of Friends of Narrabeen Lagoon stand for election in Warringah or Pittwater Council areas to represent the interests of the group. This is communicated through forums and provides an incentive for those who may not otherwise have entered the political arena to consider using their skills to benefit the community.<br>Personal growth – Forums allow for a diverse range of ideas to be aired and learning opportunities to take place. Likewise, recreational activities have mental and physical benefits which lead to personal growth. |
| Economic | Friends of Narrabeen Lagoon run cost recovery events such as recreational activities and other volunteer based activities.<br>Access to resources – Infrastructure, equipment and access to natural resources are provided in a suitable manner to the community through recreational activities. |
| Cultural | Connection to Country – In May 2012, an Aboriginal Heritage Forum was held by Friends of Narrabeen Lagoon.<br>The Narrabeen Lagoon walking map and natural features refers to the catchment being previously inhabited by the Guringai peoples who depended on abundant seafood, animals and bush foods. Additionally, the preservation of rock paintings acts as a significant linkage to cultural heritage in the area. |

**Partners**

Friends of Narrabeen Lagoon engage with Warringah and Pittwater Councils, schools (such as Narrabeen Lakes Public), the National Parks Association of NSW and various local organisations.

**Further information**

For additional information see:
http://www.narrabeenlagoon.org.au/Activities/activities.htm

### 4.2.6 Case Study 5: Roper River Landcare Group –Building Capacity to Protect the Cultural and Production Values of Mangarrayi Traditional Lands

**Roper River Landcare Group**

The Roper River Landcare Group (RRLG) was established 18 years ago, initially to tackle an infestation of Parkinsonia. It is one of the four major Pastoral Landcare Associations in the Northern Territory.

RRLG promotes the adoption of best practice NRM management in order to achieve biodiversity and sustainable land use outcomes across the Roper River catchment. In addition to weed control, the key issues facing the RRLG are:

- Feral animal control
- Tourism impacts and water quality
- Maintaining barramundi stocks
- Reducing litter and controlling erosion
- Getting local children involved in landcare
- Promoting the group and their activities.

The RRLG aims to:

- Maintain and expand networks and NRM partnerships with catchment stakeholders including Indigenous organisations, pastoralists, horticulturalists, townspeople, other NRM groups and all levels of government
- Use networks to build on stakeholder awareness of key NRM issues (weeds, threatened species, feral animals, fire) and deliver up skilling and capacity building activities.

**Background**

In 2009, The RRLG, in partnership with Mangarrayi Aboriginal Corporation, Savannah Solutions Pty Ltd and Banibi Pty Ltd, received $1.3 million in funding over 4 years from the Australian Government's Caring for our Country initiative to implement a project entitled 'Building Capacity to Protect the Cultural and Production Values of Mangarrayi

Traditional Lands'. This was one of the Landcare and Sustainable Farm Practices projects which received funding under the category of Other Funded Projects. The project addresses the national priority areas of:

- Sustainable farm practices
- Community skills, knowledge and engagement.

The funding has enabled the establishment of the Mangarrayi Rangers, a group of four Aboriginal Rangers who are using both traditional and contemporary land management practices to protect and conserve the biodiversity on the traditional Mangarrayi lands around the Roper River near Mataranka in the Northern Territory. These traditional lands include the historic Elsey Station, a 5,300 square kilometre former pastoral lease east of Mataranka, which is now Aboriginal land under the Northern Territory Land Rights Act. The Roper River, which is a permanent watercourse, flows for 100 km through Elsey Station.

In addition to establishing a Mangarrayi Ranger group, the project targets include:

- Undertaking weed control over 5,000 hectares per year
- Increasing the native habitat area
- Identifying and recording Traditional Ecological Knowledge.

Weed control is the key focus of the Ranger group. The Rangers are using chemical, fire and mechanical means to control a range of weeds including Parkinsonia, Mesquite, Prickly Acacia, Bellyache Bush and Chinee Apple. They are well equipped with a Toyota Troop Carrier, spray equipment, quad bikes and a boat, which means that they can get to areas on the river which would normally be inaccessible. The Rangers are working to a Weed Management Plan which is constantly being reviewed and updated. They provide data to the Weed Management Branch of the Northern Territory Department of Land Resource Management for inclusion in their data bank.

The Project Coordinator estimates that they have nearly eradicated Parkinsonia and that in another year or two they will be on top of Chinee Apple. Bellyache Bush, which is mainly along the banks of the Roper River, is now the biggest problem and the Rangers feel they are fighting a losing battle with it because it spreads easily and the seed bank lasts for many years. However, if they were not endeavouring to control it, the problem would be much worse than it currently is.

In addition to weed control, the Rangers are also undertaking feral animal control and have to date removed an estimated 12,000 donkeys as well as feral pigs, horses and buffalo.

The project is managed by the RRLG and has a Steering Committee comprising representatives from the Northern Land Council, Northern Territory Parks and Wildlife Service, Bushfires NT, Northern Territory Department of Land Resource Management Weed Management Branch, and Mangarrayi Elders.

**Table 8: Multiple benefits of the Mangarrayi Rangers**

| Multiple benefit | Description |
|---|---|
| Learning, awareness and practice change | Improved knowledge – In 2011 a book was published on Mangarrayi and Yangman Plants and Animals. The book was initiated by the Managarrayi community and funded through the Indigenous Literacy Foundation. It captures the knowledge of the local Elders about the plants, animals and landscapes that occur in Mangarrayi and Yangman country. The book also contains a creation period story and the outline of a ceremony.<br>Awareness raising– Posters and cards showing the names of animals and plants in local language have also been produced so that they can be used in the local school.<br>Improved knowledge – The Rangers have received training in a variety of areas including:<br>• Chem Cert<br>• quad bikes<br>• weed identification and data collection<br>• weed control<br>• small engine maintenance and safety<br>Improved knowledge – A total of 15 people have now done Cert 3 Conservation and Land Management so there is now a pool of people that can be called on for the Ranger program.<br>Scales of change – The preference within the community is that people should receive cross training so that they are able to work either in the meatworks or on country. This is because work crews are picked on a cultural basis, rather than for particular skills. All the current Rangers are from different family groups and it is the lore people who have a say on their country when they are working there.<br>Multi-generational reach (involvement of school children) – One day a week when school is in the Rangers take a couple of children who are keen to be Rangers out with them to show them what they do. The Rangers want to eventually run two camps a year for the children to take them out on country. |
| Social – community health and wellbeing | Contact with natural environment – The majority of Mangarrayi people live on the community at Jilkminggan. People are keen to get on country more often, but access to some places is difficult, they don't have suitable vehicles and they can't afford to go. When they do get the opportunity to stay on country they all enjoy the peace and quiet away from the community as well as catching good food and discovering new places. As part of the cultural mapping project, 12 people from the community went to Campbell Springs, but only one of those had been there before. |

| Multiple benefit | Description |
|---|---|
| Social – political and social capital | Increasing the recognition of women – The current Ranger group are all men, but there is interest amongst some of the more educated young women in the community in forming a women's ranger group. They are putting in for Working on Country funding and if successful, the intention is that the women rangers would look after the women's cultural sites and women's ceremonies, carry out plant and animal mapping, teach the children using the plant and animal books and manage a junior ranger program.<br>Scales of change – One of the Rangers attended a training course at Warwick in Queensland to learn how to be a supervisor at a meatworks. The Rangers are also learning how to trap crocodiles for the Northern Territory Parks and Wildlife Service and Roper River Shire Council. The Rangers are also using their weed control skills to manage the lantana on the lime mine at Elsey Station and there are potential opportunities for them to do further contract weed control work on neighbouring properties. Currently all the land is leased so fire management is the responsibility of the lessees, but next year they are planning to get all the lessees together and do some early season burning.<br>Governance/self-regulation – At the end of the project, it is intended that the Ngarranbardji Corporation will take over responsibility for the management of the Ranger group from the RRLG and that the Rangers will become self-funded through gaining contract work. The Ngarranbardji Corporation is made up of seven representatives, one from each of the seven Mangarrayi family groups. Three years ago the Board undertook governance training and they are now more confident about making decisions and they are actively putting in applications for funding from different sources to support their vision of creating employment within the community. The Board is very risk averse and utilises a lawyer, accountant and bookkeeper that are external to the community so that they cannot be accused of favouring a particular family group and also so that they have recourse if anything goes wrong. |

| Multiple benefit | Description |
|---|---|
| Economic | The Rangers are on a casual employment contract, which means that they get paid only when they work. This averages about 60 hours a fortnight, but when they are out bush they do a full week's work.<br>Access to resources – Through Caring for our Country funding, the Rangers now have access to a Toyota Troop Carrier, quad bikes, spray equipment and a boat.<br>Access to resources – The RRLG and the Mangarrayi Aboriginal Corporation has received funding from a range of different programs to undertake specific projects on the Mangarryi traditional lands. This includes:<br>• Landcare funding to use Indigenous knowledge to restore and protect the Roper River wetlands<br>• Funding to develop the Mangarryi Land and Cultural Strategic Plan 2009<br>• Funding of $1.7 million through the Get Communities Working Program to develop the Mangarrayi Meatworks. The meatworks will process cattle which don't meet the current export market specifications. The intention is that eventually 10 head a week will be processed for use by the community. |

| Multiple benefit | Description |
|---|---|
| Cultural | Connection with Country – Through their work in detecting and controlling Weeds of National Significance on their traditional lands, the Mangarrayi Rangers are helping to reduce the threats to cultural values.<br>Connection with Country – There is increasing recognition of the importance of Traditional Ecological Knowledge in maintaining the health of the Roper River Wetlands. Traditional Owners had been forbidden from damming the river at Red Lily Wetlands and when a fire went through the water level in the wetlands dropped by three metres. As part of the National Research Flagships Program Water for a Healthy Country CSIRO undertook research into the management of the Roper River and in April 2012 they produced a report "Indigenous Water Management and Water Planning in Upper Roper River". The report recognised the value of the traditional management techniques to the health of the wetlands and these have now re-commenced.<br>Connection with Country – A significant cultural mapping project has been undertaken with the Mangarrayi people. Simon Normand has involved five to eight Elders in recording their knowledge of the dreaming stories and he has represented this information as a series of painted cultural maps which will eventually cover the entire Elsey region. The maps include the Aboriginal names for every part of the land as well as showing the song lines and where they went. This work has given the Elders an opportunity to go out onto country and they have shown great interest and pride in being able to preserve their history for future generations. Some of the younger people know some songs and story lines but much of this knowledge is not now generally known within the community, so would otherwise be lost as the Elders die out.<br>Connection with Country – Simon Normand has also written three books recording the process and including interviews and photos of the sites and when the project is finished, these will be combined into a single document.<br>Connection with Country – As part of the cultural mapping project, Simon Normand has also been searching the country for books written about Elsey Station. The intention is to create a keeping place for all this information and potentially there may be an opportunity to display some of the material at the Katherine Cultural Centre.<br>Connection with Country – Increasing opportunities are being provided for Managarrayi people to go on their country. In addition to the cultural mapping project and the weekly inclusion of school children in the Ranger's trips, the RRLG and Mangarrayi Rangers organise annual cultural camps. These are held in the school holidays so that the Traditional Owners have an opportunity to visit their traditional lands. Over 100 people attended the first of these two week camps. They danced, did body painting using white ochre, and spoke language and involved the young people in traditional activities including fishing and collecting turtle eggs as well as learning Mangarrayi lore and recording the traditional names for Mangarrayi plants and animals. |

| Multiple benefit | Description |
|---|---|
| Resilience | Rangers are well respected in the community and this has increased their pride in their job. One of the Rangers has apparently moved away from Jilkminggan and has now stopped drinking alcohol.<br>When the Rangers are working on country for extended periods, they now take their wives and children too. The wives catch fish and cook for the men and the Rangers are happier to be out on the camp than they would be if they were there alone.<br>The spokesperson for the Board is now 70 but she is still very actively involved and is consulted about everything. The Board is learning to have a voice and the Board members are now confident that their views will be recognised. |

**Partners**

- Mangarrayi Aboriginal Corporation
- Roper River Landcare and Conservation Association
- Savanna Solutions Pty Ltd
- Banibi Pty Ltd.

**Further information**

For additional information, see: http://www.nrm.gov.au/projects/other-projects/landcare/mangarrayi/index.html

# 5. Multiple benefits of Landcare and NRM: A synthesis

## 5.1 Overview of multiple benefits and outcomes

In earlier sections of this report considerable comment has been made about the range of scales at which Landcare and NRM operate, the outcomes delivered by Landcare and NRM and that numerous project and program evaluations, reviews and audits have reported on the considerable success of Landcare and NRM in engaging the community and in delivering improvements in the condition of natural resources across Australia. Based on these assessments, it is clear that Landcare and NRM have been outstanding successes.

What has mostly been missing from the assessments of the success of Landcare and NRM has been the possible broader suite of benefits and outcomes, what these broader (or multiple) benefits and outcomes might deliver, and how can they be measured. This project is the first known comprehensive investigation on these broader benefits. The project has found that a comprehensive suite on multiple benefits of Landcare and NRM exist and that these are well supported by peer reviewed literature and grounded in the day to day Landcare practice shown in the case studies and interviews.

The multiple benefits and outcomes identified across six categories and operate in ways that can be simple or complex, intended or unintended, large or small. These benefits and outcomes relate well to the contemporary NRM agenda in Australia with its increasing focus on linked socio-ecological systems and resilience. The following sections provide a synthesis of the literature and practice of the multiple benefits of Landcare and NRM across these six categories with a particular focus on resilience.

### 5.1.1 Learning, awareness and practice change – well established and understood

The literature review finds widespread recognition of the capacity building role of Landcare. Capacity building has been a central tenet of the program since the middle of the Decade of Landcare in the 1990s, with a particular focus on benefits of awareness raising, practice change, improved knowledge and continuous learning. The literature review, case studies and interviews undertaken as part of this project strongly support these more well-known learning awareness and practice change benefits of Landcare and NRM and extend them into broader areas covering multigenerational reach and scales of change. Combined, these six areas make a compelling case about a range of learning, awareness and practice change-based multiple benefits of Landcare and NRM.

The ability of Landcare to deliver innovation and regionally and locally-specific outcomes across the six sub-categories was particularly evident, as was the holistic view of Landcarers, their ability to approach situations with an open mind and the finding that modest resources can and do deliver significant gains, some of which were initially unintended.

Examples from the case studies that particularly align with the literature and interviews include:

- Significant engagement of old and young people

- Formal and informal cycles of continuous learning and adaptive management
- Extensive practice change including the ability to engage with sections of the community that are otherwise difficult to reach
- Innovative training across a range of areas that much better meets the needs of Indigenous people.

The ability of Landcare and NRM to reach people who may otherwise not be engaged in Landcare and NRM activities was important in some of the case studies. For example, the fire recovery project recorded 6, 267 volunteer days, many of which were from those formerly not previously concerned with NRM and environmental issues. Other case studies included projects that focussed specifically on engaging women and younger and older people, who may otherwise not be particularly interested in Landcare and NRM. Some of these individuals have reported significant individual benefits from this involvement and translated this involvement into broader local or regional outcomes.

The learning, awareness and practice change-related multiple benefits of Landcare and NRM provide evidence of NRM practice change outcomes, heightened NRM awareness, and capacity building of individuals and groups. These also provide community infrastructure (networks and resources) that would otherwise not exist and that are often the only structured educational experience many participants have had for many years. This will contribute to the ability of individuals, communities and societies to adapt, transform, and manage when faced with unexpected changes or challenges.

### 5.1.2 Social – community health and wellbeing – complex but considerable

The literature detailing the pathways in which Landcare and NRM can improve health and wellbeing is the most complex. This does not mean it is tenuous, just that it operates in ways that are multi-faceted and interdependent. The sub categories that comprise this multiple benefit are a good indication of this complexity, covering contact with natural environment, social networks and physical and mental health benefits.

The way in which Landcare operates means that the vast majority of its activities, including the case studies in this report, provide a very real connection with the natural environment. This connection is mostly as part of a Landcare group or network. The literature reviewed is clear about the health benefits of contact with the environment and the positive physical and mental changes that can accrue as a result of this contact. Some of this literature covers Landcare-type activities, or can be reasonably extended so it does so.

The case studies and interviews were positive about the general benefits that arise from contact with the natural environment and especially positive about the very broad value of networks. They were less definitive about the physical and mental benefits that Landcare and NRM can deliver, perhaps because of the complex manner in which the literature says these benefits accrue. That said there are some very good examples identified in the case studies and interviews, as follows:

- Interviewees noted that people often come together for the common purpose of delivering a particular NRM outcome, but find social benefits that run hand-in-hand or might even exceed the original NRM target.

- The case studies which involved rehabilitation of areas opened up new natural environments for Landcarers, property owners and the surrounding community to utilise for recreational and tourism purposes.

- A truly diverse range of networks were utilised in some projects. The Upper Goulburn Landcare Network Fire Recovery Project is a particularly strong example.

- Individual mental health benefits were clearly demonstrated in several case studies and an increase in general community wellbeing was also a feature.

### 5.1.3 Social-political and social capital – a vital part of the social fabric

The literature reveals that the contribution of Landcare and NRM to social capital has been recognised in general terms since the Decade of Landcare (1990s). More recent studies provide a more comprehensive and specific set of information about the social capital benefits of Landcare. In this report, these benefits have been categorised into: partnerships and networks; leadership and public participation; governance and self-regulation; localism and empowerment; increasing the recognition of women in rural communities; personal growth; filling the void; and increasing awareness, skills and knowledge.

The categories of multiple benefit identified in the literature and supported through the interview process undertaken as part of this project give an indication about the importance of Landcare across Australia, especially in rural areas; it is an intrinsic part of the social fabric and in many cases fills gaps left by the withdrawal of services or as a result of some institutions closing down. Running hand in hand with the role Landcare plays in service provision is its focus on building networks and partnerships, sometimes in highly innovative ways. Given this it is not surprising that Landcare has in many instances become self-regulating and self-governing and has offered new leadership and public participation roles built in part by localism and empowerment.

The case studies were particularly strong with their links to the creation and maintenance of social capital. These include the following:

- extensive partnerships and networks were reported in each case study. Combined, these cover an immense diversity of organisations including educational institutions, other Landcare and NRM groups, local, state and Australian Governments, non-government organisations, churches, producer groups, professional associations and the private sector

- leadership and public participation is probably best represented by the Friends of Narrabeen Lagoon who conduct a diverse range of more typical Landcare activities. Members have stood for local government elections on a platform of representing the interests of Landcare. Other case studies reported members operating in a wide variety of leadership positions

- there is increasing recognition of women playing community leadership roles across the case studies, especially in the Naturally Resourceful Program and the Roper River Landcare Group

- the Roper River Project is a good example of the ability of Landcare to self-govern and self-organise for current conditions whilst being mindful of longer term challenges and opportunities.

### 5.1.4 Economic – a considerable set of numbers

Investing in Landcare and NRM has been demonstrated in the literature to generate significant return on investment to individual properties and leverage 2-5 times the amount invested through a variety of means. The literature also indicates three key categories of economic benefit: increased financial return to individuals, sub-catchments and regions; access to additional resources; and training and management techniques.

Landcare has often been able to attract additional resources partly owing to the scale at which some of the larger Landcare networks and groups operate which has in turn led to greater capability, more partnerships and less risk to investors. An interesting feedback loop appears to run in these cases from networks to partnerships to funding and back to networks (or "success breeds success" sometimes called the "haves" and "have-nots" of Landcare).

The Landcare ethos has helped deliver a much broader set of economic outcomes over the long term through its encouragement of farmers to push a little harder on financial risk versus reward, because of its ability to foster innovation and best practice and because the capacity building activities that are associated with Landcare tend to improve long term productivity and financial returns. The ability of Landcare to assist Indigenous people to reconnect with country leads to better participation in the market through the utilisation of natural resources to generate income.

The interviews and case studies also reported economic benefits from Landcare, both in direct and indirect terms. As examples:

- the Fire Recovery Project used external funds to generate considerable local economic activity after the bushfire, assisting the recovery of local businesses. Extensive volunteer days and financial contributions from external sources were also very valuable.

- training and management techniques in the Web of Trees Project have increased productivity and financial returns.

- the Roper River Landcare Mangarrayi Rangers Project has delivered direct economic benefit to the community including employment, capital items and via increased participation of Indigenous Australians in the market economy.

A confounding factor with economic returns of Landcare and NRM investment demonstrated in the case studies seems to be that few group projects invest solely for direct economic return. The case studies do report major economic benefits to sub-catchments and regions and (to a limited extent) to individuals, but this is mostly reported as a side benefit or as part of a triple bottom line approach to Landcare and NRM.

### 5.1.5 Cultural – increasing connections in new ways that are very old

**Connection with Country**

The project has identified significant benefits to Indigenous Australians via connection with country and that this connection is essential for their spiritual, social, physical and mental health. In some cases Landcare has helped to maintain or increase existing connections, while in others it is has recreated connections that existed for centuries prior to white settlement. In such cases, those who had a greater connection to country had significantly better outcomes across a range of indicators, including diabetes, cardiovascular risk and body mass index.

Interviewees noted the increasing importance of connection to country in Landcare and NRM activities also in relation to the use of traditional knowledge in on ground Landcare and NRM projects. Some of the case studies also reported about their engagement with Indigenous Australians in a formal partnership role or in an effort to utilise traditional knowledge. However, it is the Roper River Landcare Mangarrayi Rangers Project that best demonstrates the multiple benefits of Landcare as it relates to Indigenous Australians with outcomes including the following:

- reduced threat to cultural values
- increasing importance of traditional knowledge in the management of weeds and pests
- cultural mapping to assist with the maintenance of traditional knowledge
- social capital, including increased engagement of women and young people
- health and wellbeing outcomes.

## 5.2 Key stakeholder beneficiaries

The literature, case studies and interviews have identified the existence of a large number of multiple benefits. There is an extensive group of stakeholders from diverse areas who gain directly and indirectly from these multiple benefits. They have been separated into two groups as follows:

1. what we term the more traditional list of stakeholders that would be identified via a stakeholder analysis undertaken by a typical Landcare or NRM program or project
2. an additional set of multi-beneficiary stakeholders who benefit in ways that have not been well articulated to date. This group more truly represents the diverse beneficiaries of Landcare and NRM and aligns with the contemporary direction of NRM in Australia with its focus on resilience and linked socio-economic systems.

**Table 9: Stakeholders who derive value from the multiple benefits of Landcare and NRM**

**Typical**

| Stakeholder | Benefit provided | Multiple benefit category | Supported by |
|---|---|---|---|
| Local Council | On ground work | Social – political and social capital<br>Partnerships and networks | Interviews<br>Case studies<br>Literature |
| Regional NRM group/Catchment Management Authority | Facilitation of field days | Social – political and social capital<br>Partnerships and networks | Interviews<br>Case studies<br>Literature |
| Federal Government | Implementation of the Caring for our Country Initiative | Social – political and social capital<br>Partnerships and networks | Interviews<br>Case studies<br>Literature |
| State Government (Department of Primary Industries) | Field trials and research | Learning, awareness and practice change<br>Continuous learning | Interviews |
| Schools and colleges (e.g. TAFE) | Mentoring (e.g. Demonstration farm teaching agriculture, NRM and sustainable futures at high-school level) | Learning, awareness and practice change<br>Multigenerational reach | Interviews<br>Literature |
| Landholders | Sustainable farming techniques, carbon farming techniques, social connections. | Learning, awareness and practice change<br>Scales of change and continuous learning | Interviews<br>Case studies<br>Literature |

**Multi-beneficiary**

| Stakeholder | Benefit provided | Multiple benefit category | Supported by |
|---|---|---|---|
| Local community | Increased rural representation | Learning, awareness and practice change<br>Scales of change | Case studies<br>Literature |
| Local government | Recreational areas (e.g. the rehabilitation of the natural environment and creation recreational access and activities) | Economic<br>Access to resources | Case studies<br>Literature |

| Stakeholder | Benefit provided | Multiple benefit category | Supported by |
|---|---|---|---|
| Local business | Tourism (e.g. enhanced environmental amenities along coastlines) | Economic<br>Investment multipliers | Interviews<br>Literature |
| Local Indigenous groups | Preservation of Indigenous culture and artefacts | Cultural<br>Connection to Country | Case studies<br>Literature |
| Community organisations | The organisational capacity to continue or revitalise community events | Social – political and social capital<br>Filling the void | Interviews<br>Literature |
| Regional development organisations and local government | Economic returns to sub-catchments and regions | Economic<br>Increased financial returns<br>Investment multipliers | Literature<br>Case studies |
| Health providers | Reduced incidence of diabetes and heart disease | Cultural – Connection to Country<br>Social capital – wellbeing | Literature<br>Case studies |

## 5.3 Resilience – the end game of multiple benefits?

Sections 5.1 and 5.2 provide a summary of the multiple benefits of Landcare and NRM along with a list of stakeholders who derive value from these multiple benefits. This results in an extensive list of benefits, supported by both literature and in practice, as well as a considerable list of stakeholder beneficiaries. A challenge then arises in bringing all of these together to ascertain the overall contribution of the multiple benefits. One way to do this is to consider the ways in which multiple benefits contribute to building resilience.

As noted in the definitions and literature review (Sections 2.1 and 3 respectively), resilience approaches are gaining increased traction in the contemporary delivery of Landcare and NRM in Australia. At national level, resilience is now a key focus of the Australian Government's investment in NRM, biodiversity and climate change programs through Caring for Our Country, the Clean Energy Future and other initiatives.[5] For this and other reasons, resilience will be a key component of all regional NRM plans as they are updated in coming years and in the on ground delivery of Landcare and NRM.

While resilience thinking is not new, it is only recently that it has started to be used in a practical sense in Landcare and NRM, where its application is based on three general steps: describe a system; assess its resilience; and then manage its resilience (Walker

[5] Department of Sustainability, Environment, Water, Population and Communities (2012). One Land – Many Stories: Prospectus of Investment. Accessed online 8/1/13 at: www.environment.gov.au/prospectus.

and Salt 2012). In applying these steps, Walker and Salt (2012) note ten key points that should be considered. A multiple benefits approach to Landcare and NRM interacts across all ten key points but is particularly emphasised in five of them, which are in **bold** below:

1. Systems are self-organising.
2. There are limits to a systems self-organising capacity.
3. **Systems have linked social, economic and biophysical domains.**
4. **Self-organising systems move through adaptive cycles.**
5. **Linked adaptive cycles function across multiple scales.**
6. There are three related dimensions of resilience: specified resilience, general resilience and transformability.
7. **Working with resilience involves both adapting and transforming.**
8. **Maintaining or building resilience comes at a cost.**
9. Resilience is not about knowing everything.
10. Resilience is not about not changing.

When managing for resilient landscapes, the above ten points are considered and interventions made where necessary so that the systems do not cross thresholds into undesirable states. Multiple benefits can assist significantly with the desire to create or maintain resilient systems, especially in the social and economic domains, which have to date been the subject of much less consideration in Landcare and NRM.

### 5.3.1 The contribution of multiple benefits to resilient landscapes

The multiple benefits approach to Landcare and NRM fits well with the contemporary NRM agenda and will fill some gaps in the practical implementation of resilience thinking in Australia. Table 10 provides summary information on the application of the multiple benefits in a resilience context. This table should be read on the understanding that it is indicative only; some of the resilience principles are tightly linked and highly interdependent, as is their application in a multiple benefits framework.

**Table 10: Interaction of selected key resilience principles with multiple benefits of Landcare and NRM**

| Resilience principal | Multiple benefit application and example |
|---|---|
| Systems have linked social, economic and biophysical domains | The multiple benefits in this report mostly function across the social and economic domains but are also clearly linked to the biophysical domain.<br>Managing for resilience requires management across multiple domains. Assessing and managing the social and economic domains has been somewhat problematic in the practical delivery of resilience principles to date.<br>The social capital and cultural categories of multiple benefits are useful in looking at linked social and biophysical domains, with the Roper River Mangarrayi Rangers and Fire Recovery Projects providing the strongest case study examples of these multiple benefits in action. |
| Self-organising systems move through adaptive cycles | Adaptive cycles are heavily emphasised in the multiple benefits categories covering learning, awareness and practice change and social capital. In these two categories, resilience was built and/or maintained in ways that included formal and informal adaptive management cycles.<br>The Fire Recovery Project case study is a good example of this principle in action, as is the Web of the Trees. |
| Linked adaptive cycles function across multiple scales | The multiple benefit category that best applies here is social capital, which requires partnerships, networks and delivery across a range of institutional and spatial scales. However, adaptive cycles were present in other multiple benefit categories and there were also a range of links across and between categories.<br>As noted in Section 5.2, the case studies have an array of individual and institutional beneficiaries of multiple benefits. These operate at scales from local to nationwide. The systems in all case studies also work across multiple scales, but this is especially emphasised in the Naturally Resourceful project which operates across various spatial and institutional scales in Queensland. |

| **Resilience principal** | **Multiple benefit application and example** |
|---|---|
| Working with resilience involves both adapting and transforming | Most of the literature and all of the case studies were strong on the need for adaptation, and provided examples of where this had occurred.<br>A smaller segment of the literature specifically covered transformation, even though in some cases transformations had occurred or were sought. The case studies and interviews are similar in this regard. This may be a factor of the terminology used in resilience thinking with "transformation" not yet being commonly used in Landcare.<br>All case studies had an aspect of adaptation to a changing natural environment. The Fire Recovery Project was the best example of a transformation in action and also of the adaptation needed after a transformation. The best example of an integrated adaptation addressing multiple outcomes is the Roper River Mangarrayi Rangers project. |
| Maintaining or building resilience comes at a cost | This principle includes economic cost, so at first glance the multiple benefits dealing with economics and the direct economic returns and costs of all projects appear to be most relevant. The principle is also concerned with enhancing resilience which can reduce economic efficiency (e.g. by building reserves of various types). With this in mind, the benefits that are most relevant are economic as well as those covering social capital and community health and wellbeing.<br>The case study that best represents the approach of balancing economic cost with economic benefit and enhanced resilience is the Web of Trees project. |

### 5.3.2 Resilience and capacity to handle major challenges

The examples in Table 10 (above) provide an overview of practical ways in which the multiple benefits of Landcare and NRM function against key resilience principles, as revealed in the case studies undertaken as part of this project. Table 10 also contains examples of the ways in which the multiple benefits assist communities to build resilience, which helps them better handle major challenges such as bushfires, social change and drought.

The collective evidence gathered through the literature review, case studies and interviews undertaken for this project shows that Landcare groups have a proven track record of providing community leadership and coordination of disaster recovery efforts and working alongside other service providers and volunteer groups. This evidence indicates that the ability of Landcare groups to handle challenges such as natural disasters is due to the influence, interaction and delivery of a large number of multiple benefits, especially those covering social capital and wellbeing. Landcare groups are typically made of individuals who live locally, are familiar with the area (geographically and socially), are well placed to respond quickly to community needs in a coordinated

manner, are members of other groups and networks locally and regionally and have had the opportunity to develop leadership skills.

Many Landcare groups have demonstrated the ability to coordinate rapid response activities and longer term recovery projects, as well as providing ongoing support and a communication system within the community. Some of the Landcare groups that were a part of this study were able to mobilise people quickly after a disaster and had the structures and frameworks in place to offer immense potential to respond to crisis. The groups also provide a conduit for the receipt of funding and the rapid delivery of projects.

The case studies and literature also clearly show that the multiple benefits of Landcare and NRM help to develop communities that are better prepared to cope with a range of slower moving changes including the transition to genuinely sustainable farming, biodiversity loss, urban growth and climate change. The formation of networks that deliver individual and community support and increased social cohesion (through social, economic, environmental and cultural considerations) are particularly important here.

## 5.4 Measuring multiple benefits – suggested key measurable indicators

The key successes of Landcare and NRM to date have largely been reported in the biophysical domain (Section 2.4). The evidence gathered in this project indicates the development of indicators for multiple benefits relating to Landcare, NRM and resilience is an emerging area of both theory and practice (e.g. Walter and Salt, 2012, Dale et al, 2011, Ross et al, 2010). However, some related work has existed for some time (King and MacGregor, 2000) and can be applied in this context.

A good graphical explanation of a possible approach to measure multiple benefits appears in Ross et al (2010), who provide a draft conceptual framework for regional level monitoring and reporting of social resilience that was intended to guide ongoing research (Figure 5). This identifies the key social resilience domains of human, social, natural, physical, financial, cultural and governance which are similar to the present project.[6]

The suggested approach of Ross et al (2010) has been enhanced and extended by Dale et al (2011). King and MacGregor (2000) also provide a graphical representation of social indicators and monitoring criteria which they refer to as social indicators, constructs and models and they apply them the context of vulnerability, not resilience.

We have adapted aspects of Ross et al (2010), Dale et al (2011) and King and MacGregor (2000) to develop a graphical representation of indicators, the categories of multiple benefits they relate to and their interface with multiple benefits and resilience (Figure 6). We have also suggested a set of multiple benefits indicators (Table 11).

---

[6] Note that this method is at conceptual level, and a slightly different approach has been used for the present project.

**Figure 5: A conceptual framework for regional level monitoring and reporting of social resilience**

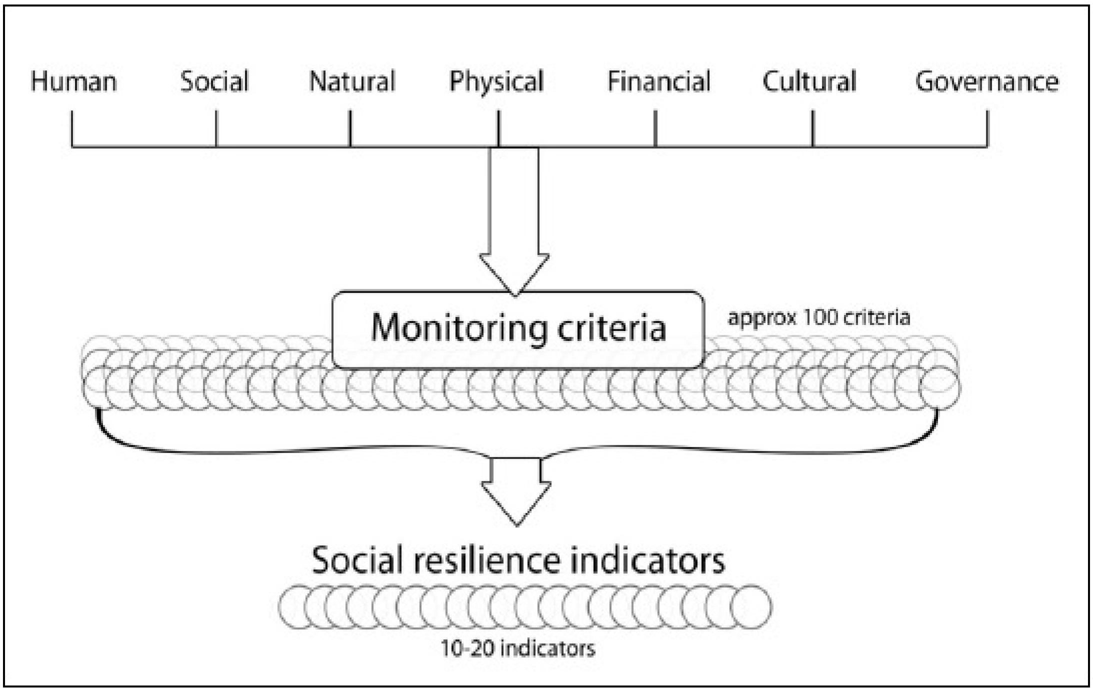


Source: Ross et al (2010) p16. NB: that this is at conceptual level only and different approaches have been used for the present project.

**Figure 6: Links between indicators and categories of multiple benefits**

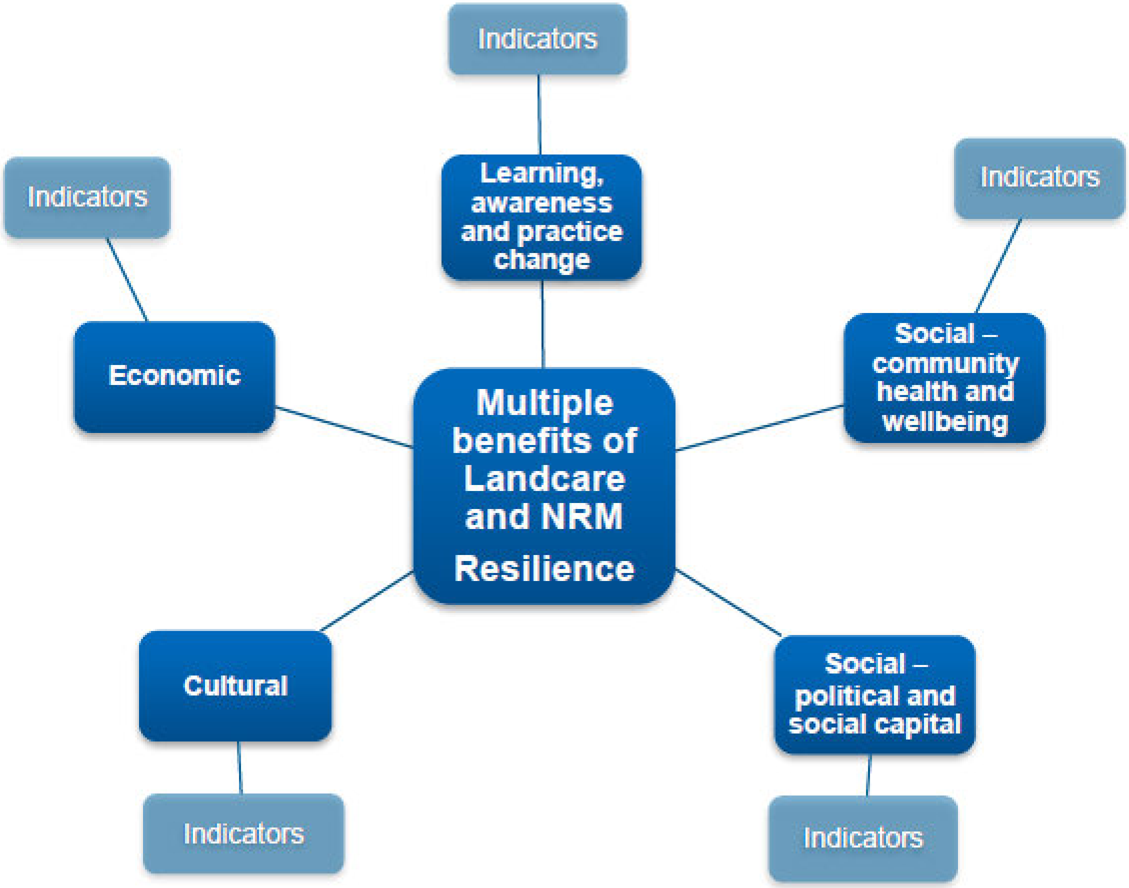


Fourteen multiple benefit indicators have been developed (Table 11). In developing these indicators, the following factors were considered:

- The results of the interviews (Section 4.1), case studies (Section 4.2) and comments from the Department of Agriculture and the ALC about possible key indicators for multiple benefits of Landcare and NRM and their perspectives about how these indicators could be used to monitor multiple benefits.
- Key findings from literature, particularly Walter and Salt (2012), Dale et al (2011), Ross et al (2010) and King and MacGregor (2000).
- The Landcare and NRM community of the GHD and EEA team, especially in relation to what indicators would make sense at a project and regional level.
- The need for the indicators to provide a cross section of multiple benefits and be reasonably simply measured or possibly utilise existing data.
- The idea that the indicators should easily work at a range of scales so they can be used by various groups and organisations both inside and outside of current Landcare and NRM arrangements (e.g. a Landcare group, a Landcare network, a regional NRM organisation, the Australian Government, a regional or local health organisation).

- The opportunity to use existing data to generate information and knowledge about the multiple benefits of Landcare and NRM, or, as a second best, to create new data as cost effectively as possible.

A range of detailed monitoring criteria are suggested. These may be measured through a variety of approaches including a specific survey, the use of existing local, state or regional data, or an enhancement of Australian Government or regional level monitoring, evaluation, reporting and improvement (MERI) programs. Some of these approaches would need to be developed and delivered by specialists while in other cases simple surveys that work at local level could be developed for smaller local organisations to deliver, with results potentially aggregated at regional or state level.

Any expansion of the MERI effort for Australian Government programs would need to be carefully evaluated against the existing standard outputs and protocols. This may be achieved through a small extension of current project reporting to generate data that can again be aggregated at local, regional or national level. This extension does not need to be complex. A modification of some ABARES data collection, such as that reported by Ecker et al (2011), would also prove useful. Specific and detailed local health surveys may be required for one indicator; this would be expensive to undertake.

**Table 11: Description of indicators**

| Category | Indicator | Monitoring criteria |
|---|---|---|
| Learning, awareness and practice change | **Multigenerational reach**: diversity of ages | 1. Proportion of young people within groups relative to regional demographics (e.g. under 25).<br>2. Level of program activity (e.g. succession planning, other peer group programs). |
| Learning, awareness and practice change | **Scales of change**: instances of change (groups or individuals) | 3. The instances of groups which have performed functions outside of traditional NRM areas.<br>4. Individuals that have taken on greater responsibility at a broader scale and directly as a result of Landcare (community leadership roles). |
| Learning, awareness and practice change | **Continuous learning**: additional courses or training | 5. The number of people within a group who have received external funding and attended further training (e.g. additional courses) as a result of their participation in Landcare (exposure to group learning). |
| Social – community health and wellbeing | **Physical and mental health benefits** | 6. Impact of involvement in Landcare and NRM via specific local research. |

| Category | Indicator | Monitoring criteria |
|---|---|---|
| Social – political and social capital | **Partnerships and networks** | 7. The number and quality of horizontal and vertical linkages with other groups. Source: Jennings et al 2010 (Participation in networks). |
| Social – political and social capital | **Recognition of women in rural communities** | 8. Participation of women within the group.<br>9. Participation of women which has led to further community involvement and recognition of women. |
| Social – political and social capital | **Filling the void** | 10. Defunct functions now performed by a Landcare group. |
| Economic | **Increased financial return** | 11. Return on investment from Landcare and NRM. |
| Economic | **Access to resources** | 12. Resources that are additional to those serviced by the group which also build resilience (e.g. corporate sponsorship, outside volunteer hours, donations of goods and services). |
| Economic | **Investment multipliers** | 13. Level of contributions in cash or in kind to projects or programs. Investment multipliers for each dollar of group's investment. Source: Jennings et al 2010 (Local resource mobilisers). |
| Cultural | **Connection to Country**: number of opportunities to be on country | 14. The number of opportunities to be on country as a result of Landcare. |

# 6. Summary of findings

## 6.1 The multiple outcomes and benefits (social, economic, cultural, health, learning, awareness and practice change and community) that result from Landcare and NRM

The literature, case studies and interviews carried out in this project have revealed that Landcare and NRM contribute to a diverse array of multiple outcomes and benefits in the social and economic domain. These cross six key categories and numerous sub-categories (Table 12).

**Table 12: Categories of multiple benefits**

| Categories | Sub-categories |
|---|---|
| Learning, awareness and practice change | Awareness raising<br>Practice change<br>Multigenerational reach<br>Improved knowledge<br>Scales of change<br>Continuous learning |
| Social – community health and wellbeing | Contact with natural environment<br>Social networks<br>Physical and mental health benefits |
| Social – political and social capital | Partnerships and networks<br>Leadership and public participation<br>Governance and self-regulation<br>Localism and empowerment<br>Increasing the recognition of women in rural communities<br>Personal growth<br>Filling the void<br>Increasing awareness, skills and knowledge |
| Economic | Increased financial return<br>Access to resources<br>Training and management techniques |
| Cultural | Connection with Country |
| Resilience | Resilient people and resilient landscapes |

## 6.2 The contribution of the outcomes and benefits to building community resilience and capacity to handle major challenges

The multiple benefits of Landcare and NRM contribute to resilience and in turn capacity to handle major challenges in many ways. In keeping with resilience principles, these contributions operate across linked socio-ecological systems which can sometimes be complex and difficult to represent.

A simpler way to understand the contribution of multiple benefits to resilience is to consider the categories and sub-categories of multiple benefits (Table 12), their link to multiple benefits and resilience (Figure 6) and how these relate to what Walker and Salt (2012) call the attributes of a resilient world. This appears in visual form in Figure 7.

**Figure 7: Contribution of multiple benefits to attributes of a resilient system**

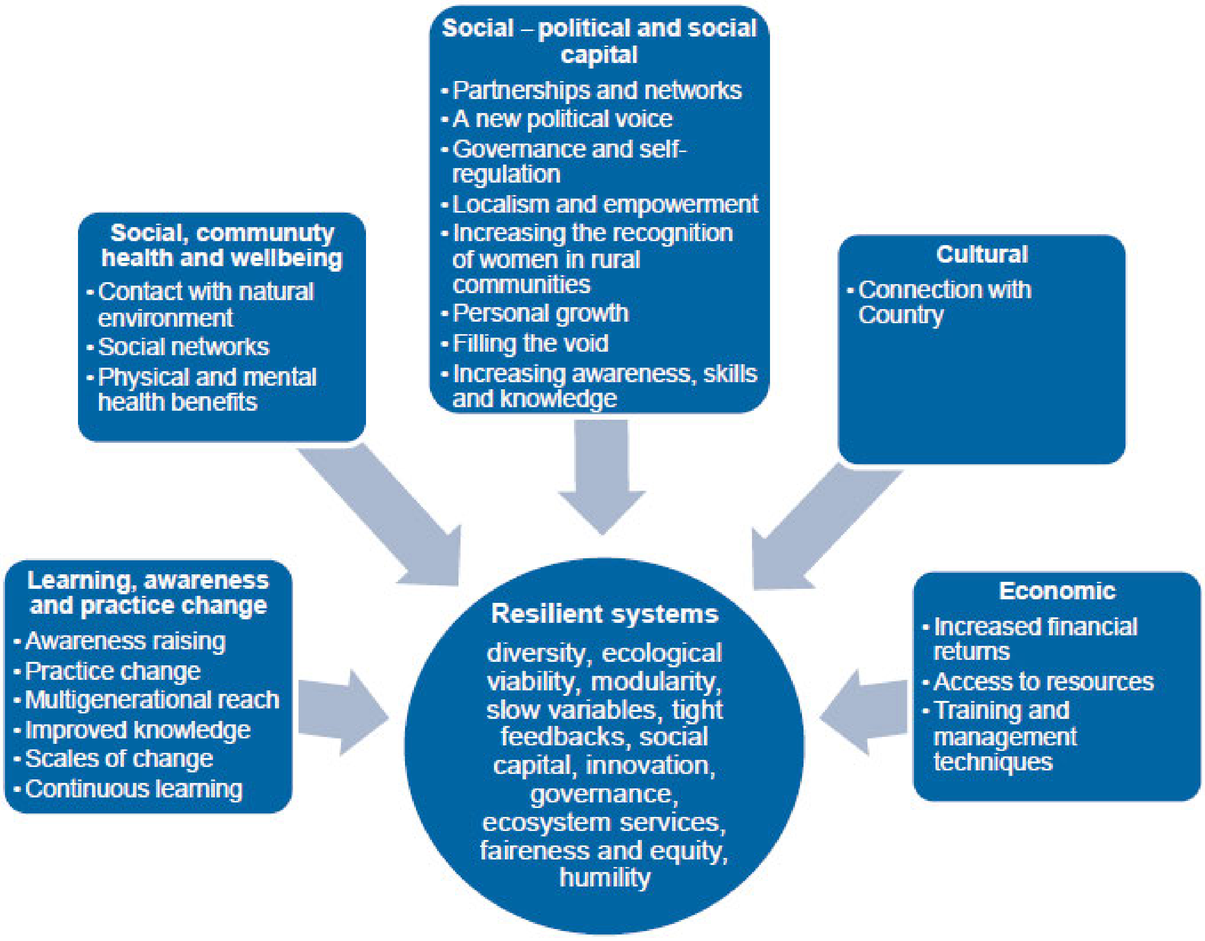

## 6.3 How can multiple benefits and outcomes be monitored to demonstrate returns on NRM/Landcare investment

The project has identified a range of indicators that can be used to monitor returns on Landcare and NRM investment. These indicators are based on sound theory and also work well in the practical delivery of Landcare and NRM. The indicators are also deliberately simple (not simplistic), work at multiple scales and can be can be tracked on an ongoing basis (Table 13).

The suggested indicators provide the best way to monitor multiple benefits, but it is important to note that the indicators are just that, and will need to be implemented and assessed in a comprehensive manner.

**Table 13: Description of indicators**

| Category | Indicator | Monitoring criteria |
|---|---|---|
| Learning, awareness and practice change | **Multigenerational reach**: diversity of ages | 1. Proportion of young people within Groups related to regional demographics (e.g. under 25).<br>2. Level of program activity (e.g. succession planning, other peer group programs). |
| Learning, awareness and practice change | **Scales of change**: instances of change (groups or individuals) | 3. The instances of groups which have performed functions outside of traditional NRM areas.<br>4. Individuals that have taken on greater responsibility at a broader scale and directly as a result of Landcare (community leadership roles). |
| Learning, awareness and practice change | **Continuous learning**: additional courses or training | 5. The number of people within a group who have received external funding and the attended further training (e.g. additional courses) as a result of their participation in Landcare (exposure to group learning). |
| Social – community health and wellbeing | **Physical and mental health benefits** | 6. Impact of involvement in Landcare and NRM via specific local research. |
| Social – political and social capital | **Partnerships and networks** | 7. The number and quality of horizontal and vertical linkages with other groups. Source: Jennings et al 2010 (Participation in networks). |

| Category | Indicator | Monitoring criteria |
|---|---|---|
| Social – political and social capital | **Recognition of women in rural communities** | 8. Participation of women within the group.<br>9. Participation of women which has led to further community involvement and recognition of women. |
| Social – political and social capital | **Filling the void** | 10. The number of people that indicate a defunct function which is now performed by the Landcare group. |
| Economic | **Increased financial return** | 11. Return on investment from Landcare and NRM. |
| Economic | **Access to resources** | 12. Resources that are additional to those serviced by the group which also build resilience (e.g. corporate sponsorship, outside volunteer hours, donations of goods and services). |
| Economic | **Investment multipliers** | 13. Level of contributions in cash or in kind to projects or programs. Investment multipliers for each dollar of group's investment. Source: Jennings et al 2010 (Local resource mobilisers). |
| Cultural | **Connection to Country**: number of opportunities to be on country | 14. The number of opportunities to be on country as a result of Landcare. |

## 6.4 How should the multiple benefits and outcomes be communicated to agencies and organisations outside the NRM sector?

Landcare and NRM clearly align with and support a number of national, state and local priorities and initiatives. At present the alignment is most visible in the biophysical domain although resilience approaches, especially those being pursued by the NSW and some Victorian Catchment Management Authorities, are starting to extend NRM into the social and economic domain. This extension is one of the significant challenges and opportunities presented by resilience thinking.

It is suggested that communicating with a broad range of stakeholders is a little premature at this point. Instead further refining and testing of the indicators is recommended and this will build a stronger evidence base which will more clearly detail the multiple benefits in the social and economic domain. Once this is done, it is suggested that a comprehensive engagement strategy be developed to begin the process of communicating about multiple benefits to a wide range of stakeholders.

### 6.4.1 As an initial set of ideas for stakeholder engagement

During the project, GHD asked key stakeholders what communication methods best demonstrate NRM program/activity benefits to outside agencies and organisations and

what mechanisms of communication have you used/been presented in the past which have been successful in demonstrating program/activity benefits to secure funding?

Responses were varied, with suggested communication methods including:

- Case studies
- Literature reviews
- Landcare facilitators, successful project administrators or landholders who could champion on-ground activities and be used a guest speakers where relevant
- Presentations to small, targeted audiences
- Press coverage (local to national scale) – radio and media releases
- Websites and web portals (e.g. http://learningforsustainability.net)
- Social media (e.g. YouTube, Facebook, Twitter)
- Cost benefit analyses of programs and activities
- Diagrammatical and graphic representation (e.g. contextualising stories with images)
- Linking the story to something visual (e.g. web-link to a video or photos taken over time which demonstrate progress)
- Use best cases in which the average person can understand the benefits
- Journal articles (e.g. credible science based information)
- Position Landcare coordinators as facilitators (change agents) rather than project managers
- Implement a problem solving framework (e.g. community adopting to climate change)
- Journal articles (e.g. Australasian Journal of Environmental Management)

Given the multiple benefits approach is new and that it brings in some innovative and difficult concepts, all of the above approaches should be actively considered in an engagement strategy. Social media offers an emerging opportunity for measuring some of the multiple benefits of Landcare and NRM in an innovative and cost effective way. This is an emerging area of evaluation that is beyond the scope of this report.

Some feedback noted that there is a saturation of case studies, consultation and literature. Additionally, the strategies used in communicating need to be adaptive to different audiences. Some suggestions were made by interviewees and those involved in case studies in relation to target audience. In order of importance, these were seen to be Australian Government bodies (specifically Treasury), local government, state government and corporates.

Tailoring a specific message to a target audience requires the use of multiple pathways. The key theme was to make the communication material short, sharp and accessible (through graphic representation) and include direct linkages to the on-ground resource (such as the facilitator or champion of flagship programs).

## 6.5 Recommendations

In an effort to progress the findings of this report, four recommendations are made:

### Recommendation 1 – Increase general awareness of multiple benefits

Develop the summary section of the report into a document and/or fact sheet that is widely publicised and distributed through existing Landcare and NRM Networks.

### Recommendation 2 – Refine indicators

Develop and implement a project to test and refine the suggested indicators and undertake additional data discovery to better understand what data is available for measuring multiple benefits and what needs to be generated. An expert panel approach involving key stakeholders at a range of scales would work well and help establish links to other relevant initiatives (eg Landcare group, Landcare network, local government, regional NRM organisation, regional development organisation, state government and Australian government).

### Recommendation 3 – Pilot indicators

Further test and refine the indicators via a pilot approach in a range of situations and scales (Landcare group, Landcare network, local, regional, state and national) and establish links to other relevant initiatives.

### Recommendation 4 – Encourage adoption

Publicise the results of the "testing and refining" project and pilot approach to the wide range of stakeholders who benefit from the multiple benefits of Landcare and NRM via a comprehensive engagement strategy.

This project has revealed a diverse set of multiple benefits and outcomes of Landcare and NRM that are well supported by literature and practice. While this project is the first step in the multiple benefits approach, and some gaps remain, there is scope to implement the project's findings as part of the evolution of Landcare and NRM to the delivery of genuinely triple bottom line outcomes. Such outcomes are likely to cover an increasingly diverse range of multiple benefits that will contribute to the increased resilience of individuals, communities, landscapes and regions.

# Appendices

## Appendix A References

The following reference list includes all references referred to in the main body of the report, as well as those referred to in the evidence synthesis.

Altman, J.C., Whitehead, P.J., 2003. Caring for country and sustainable Indigenous development: opportunities, constraints and innovation (CAEPR Working Paper No. 20). Centre for Aboriginal Economic Policy Research.

Ashby, J., 2003. Introduction: Uniting Science and Participation in the Process of Innovation–Research for Development.

Australian Bureau of Statistics (ABS), 2004. Measuring Social Capital, An Australian Framework and Indicators, Cat 1378.0. Belconnen, ACT.

Australian Public Service Commission, 2007. Tackling Wicked Problems: A Public Policy Perspective. Australian of Australia, Canberra.

Baker, R., 1997. Landcare: policy, practice and partnerships. Australian Geographical Studies 35, 61–73.

Bammer, G., O'Connell, D., Roughley, A., Syme, G., 2005. Integration research for natural resource management in Australia: An introduction to new challenges for research practice. Journal of Research Practice 1, Article–E1.

Barr, N., Cary, J., 2000. Influencing improved natural resource management on farms. Bureau of Rural Sciences, Canberra.

Beeton, S., 2002. Entrepreneurship in rural tourism? Australian Landcare programs as a destination marketing tool. Journal of Travel Research 41, 206–209.

Beilin, R., 1995. The construction of woman in Landcare: Does it make a difference? Rural Society 5, 20–29.

Beilin, R., Reichelt, N., 2010. Community Landcare: a key player in building social-ecological resilience networks? The State of Victoria Department of Sustainability and Environment, Melbourne.

Bradshaw, B., 2003. Questioning the credibility and capacity of community-based resource management. The Canadian Geographer/Le Géographe canadien 47, 137–150.

Broderick, K., 2005. Communities in catchments: implications for natural resource management. Geographical Research 43, 286–296.

Brown, V.A. (Ed.), 1996. Landcare Languages: Talking to each other about living with the land. Department of Primary Industries and Energy, Canberra, ACT.

Brown, V., 1997. Landcare in Australia: talking local sustainability in policy, practice and place (RDFN Paper No. 20e). Rural Development Forestry Network, London, UK.

Brumby, S., Willder, S., Martin, J., 2010. Milking their health for all its worth? Improving the health of farming families through facilitated learning. Extension Farming Systems Journal 6, 1–11.

Buchy, M., Race, D., 2001. The twists and turns of community participation in natural resource management in Australia: what is missing? Journal of Environmental Planning and Management 44, 293–308.

Buikstra, E., Ross, H., King, C.A., Baker, P.G., Hegney, D., McLachlan, K., Rogers-Clark, C., 2010. The components of resilience - perceptions of an Australian rural community. Journal of Community Psychology 38, 975–991.

Bunch, M.J., Morrison, K.E., Parkes, M.W., Venema, H.D., 2011. Promoting health and well-being by managing for social-ecological resilience: The potential of integrating ecohealth and water resources. Ecology and Society 16, 6.

Burgess, C., Berry, H., Gunthorpe, W., Bailie, R., 2008. Development and preliminary validation of the "Caring for Country" questionnaire: measurement of an Indigenous Australian health determinant. International Journal for Equity in Health 7, 26.

Burgess, C.., Johnston, F.H., 2007. Healthy Country Healthy People. Indigenous natural and Cultural Resource Management and Health.

Burgess, C.P., Johnston, F.H., Berry, H.L., McDonnell, J., Yibarbuk, D., Gunabarra, C., Mileran, A., Bailie, R.S., 2009. Healthy country, healthy people: the relationship between Indigenous health status and "caring for country". Med. J. Aust. 190.

Burgess, C.P., Johnston, F.H., Bowman, D.M.J.S., Whitehead, P.J., 2005. Healthy country: healthy people? Exploring the health benefits of indigenous natural resource management. Aust N Z J Public Health 29, 117–122.

Campbell, A., 1990. Landcare: Progress across the nation (First Annual Report). National Soil Conservation Program.

Campbell, A., 1992. Taking the long view in tough times: Landcare in Australia (Third Annual Report), National Landcare Facilitator. National Soil Conservation Program.

Campbell, A., 1994. Community First: Landcare in Australia, Gatekeeper Series. Sustainable Agriculture Programme of the International Institute for Environmental Development, London, UK.

Campbell, A., 1995a. Land literacy in Australia. Journal of Soil and Water Conservation 126–131.

Campbell, C.A., 1995b. Landcare: Participative Australian approaches to inquiry and learning for sustainability. Journal of Soil and Water Conservation 50, 125–131.

Campbell, A., 1995c. Facilitating Landcare: Conceptual and practical dilemmas. Rural Society 5, 13–19.

Campbell, C.A., 1995d. Landcare: Participative Australian approaches to inquiry and learning for sustainability. Journal of Soil and Water Conservation 50, 125–131.

Campbell, A., 1998. Issues in Landcare, in: Vanclay, F., Lockie, S. (Eds.), Critical Landcare. University of Central Queensland Press, p. 8.

Campbell, C.A., 2009. It's time to renew Landcare. Agricultural Science 30–33.

Campbell, B., Sayer, J.A., Frost, P., Vermeulen, S., Pérez, M.R., Cunningham, A., Prabhu, R., 2001. Assessing the performance of natural resource systems. Conservation Ecology 5, 22.

Campbell, A., Siepen, G., 1994. Landcare: Communities Shaping the Land and the Future. Allen & Unwin.

Cannon, R., 2008. The social determinants of health (SACOSS Information Paper). South Australian Council of Social Service, Unley, SA.

Carr, A., 1995. Innovation of diffusion: Landcare and information exchange. Rural Society 5, 56–66.

Cary, J., Webb, T., 2000. Community landcare, the National Landcare Program and the landcare movement: the social dimensions of landcare. Bureau of Rural Science, Canberra, ACT.

Cary, J., Webb, T., 2001. Landcare in Australia: Community participation and land management. Journal of Soil and Water Conservation 56, 274–278.

Catacutan, D., Neely, C., Johnson, M., Youl, R. (Eds.), 2009. Landcare: Local Action - Global Progress. World Agroforestry Centre, Nairobi, Kenya.

Cheng, A.S., Kruger, L.E., Daniels, S.E., 2003. "Place" as an integrating concept in natural resource politics: Propositions for a social science research agenda. Society & Natural Resources 16, 87–104.

Colliver, R., 2011. Community-based governance in social-ecological systems: An inquiry into the marginalisation of Landcare in Victoria, Australia (PhD of Philosophy Thesis).

Colliver, R., Lucas, D., Moore, S., 2008. Agent, partner or activist: the role of community Landcare in creating sustainable rural landscapes, in: Community Development and Ecology: Engaging Ecological Sustainability Through Community Development. Presented at the International Eco Community Conference, Murdoch University, Melbourne, Victoria, p. 12.

Comerford, E., 2004. Choosing between incentive mechanisms for natural resource management. A practical guide for regional NRM bodies in Queensland. Queensland Department of Natural Resources and Mines, Brisbane, Queensland.

Australian of Australia, 2012a. Caring for our Country, An outline for the Future, 2013-2018.

Australian of Australia, 2012b. Australian Government Land and Coasts, Caring for our Country, Business Plan 2012-13.

Australian of Australia, 2012c. Caring for our Country 2012, <http://nrm.gov.au/contact/officers.html>.

Compton, E., Beeton, R.J.S., 2012. An accidental outcome: Social capital and its implications for Landcare and the “status quo”. Journal of Rural Studies 28, 149–160.

Compton, E., Hayne, W., Beeton, R.J.S., 2007. Six years in a cement mixer: A landcare journey through changing times. Presented at the International Landcare Conference, Melbourne, Victoria.

Compton, E., Prager, K., Beeton, B., 2009. Landcare bowling alone: Finding a future in the “fourth” phase, in: Lane, M., Robinson, C., Taylor, B. (Eds.), Contested Country: Local and Regional Natural Resource Management in Australia. CSIRO, Collingwood, Victoria, pp. 147–160.

Cooke, P.R., 2010. Education in Landcare groups: Social learning aspects of adaptive management. Extension Farming Systems Journal 6, 139–144.

Coree Consulting Pty Ltd, 2003. Landcare Literature Review. Coree Consulting Pty Ltd.

Cullen, P, Williams, J, Campbell, A (2003). Landcare Farming: Securing the future for Australian Agriculture. Landcare Australia Limited.

Cullen-Unsworth, L., Hill, R., Butler, J. and Wallace, M., 2011. Development of Linked Cultural and Biophysical Indicators for the Wet Tropics World Heritage Area. International Journal of Science in Society Volume 2, Issue 4, Champaign Illinois.

Curtis, A., 1995a. Landcare in Australia: A critical review (report No. 33). Charles Sturt University, Albury, NSW.

Curtis, A., 1995b. Landcare in Victoria: The state of play (Report for Landcare Victoria No. 24). Johnstone Centre, Charles Sturt University, Albury, NSW.

Curtis, A., 1998. Agency-community partnership in Landcare: Lessons for state-sponsored citizen resource management. Environmental Management 22, 563–574.

Curtis, A., 1999. Landcare in Victoria: Beyond onground work (Report No. 125). Johnstone Centre, Charles Sturt University, Albury, NSW.

Curtis, A., 2003. Reflecting on the Landcare experience: A report based on information held within ABARE and BRS. Bureau of Rural Sciences.

Curtis, A., Birckhead, J., De Lacy, T., 1995. Community participation in Landcare policy in Australia: The Victorian experience with regional Landcare plans. Society and Natural Resources 8, 415–430.

Curtis, A., Britton, A., Sobels, J., 1999. Landcare networks in Australia: State-sponsored participation through local organizations. Journal of Environmental Planning and Management 42, 5–21.

Curtis, A., Cooke, P., 2006. Landcare groups in Victoria: After twenty years (Report to the Australian Government Deparment of Agriculture, Fisheries and Forestry, National Landcare Program, Monitorin and Evaluation Project). Instutute for Land, Water and Society, and the National Landcare Program, Albury, NSW.

Curtis, A., Davidson, P., De Lacy, T., 1997. Women’s participation and experience of Landcare in Australia. Journal of Sustainable Agriculture 10, 37–56.

Curtis, A., De Lacy, T., 1995a. Evaluating landcare groups in Australia: How they facilitate partnerships between agencies, community groups, and researchers. Journal of Soil and Water Conservation January-February, 15–20.

Curtis, A., De Lacy, T., 1995b. Examining the assumptions underlying Landcare. Rural Society 44–55.

Curtis, A., De Lacy, T., 1996a. Landcare in Australia: Does it make a difference? Journal of Environmental Management 46, 119–137.

Curtis, A., De Lacy, T., 1996b. Landcare in Australia: Beyond the expert farmer. Agriculture and Human Values 13, 20–32.

Curtis, A., De Lacy, T., 1996c. Landcare in Australia: Does it make a difference? Johnstone Centre, Charles Sturt University, Albury, NSW.

Curtis, A., De Lacy, T., 1998. Landcare, stewardship and sustainable agriculture in Australia. Environmental Values 7, 59–78.

Curtis, A., De Lacy, T., Klomp, N., 1993. Asessing the effectiveness of landcare groups: The Victorian experience. Regional Journal of Social Issues 27, 78–91.

Curtis, A., Lockwood, M., 2000. Landcare and catchment management in Australia: Lessons for state-sponsored community participation. Society & Natural Resources 13, 61–73.

Curtis, A., Lucas, D., Nurse, M., Skeen, M., 2008. Achieving NRM outcomes through voluntary action: Lessons from Landcare (Discussion Paper). Charles Sturt University & Victorian Department of Sustainability and Environment, Melbourne, Victoria.

Curtis, A., Sample, R., 2010. CBNRM in Victoria: Contributing to dialogue, learning and action (ILWS Report No. 55). Institute for Land, Water and Society, Charles Sturt University, Albury, NSW.

Curtis, A., Van Nouhuys, M., 1999. Landcare participation in Australia: The volunteer perspective. Sustainable Development 7, 98–111.

Curtis, A., Van Nouhuys, M., Robinson, W., MacKay, J., 2000. Exploring Landcare effectiveness using organisational theory. Australian Geographer 31, 349–366.

Department of Agriculture, 2003. Review of the National Landcare Program (Report). Department of Agriculture, Canberra, ACT.

Dale, A., Vella, K., Cottrell, A., Pert, P., Stephenson, B., King, D., Boon, H., Whitehouse, H., Hill, R., Babacan, H., Thomas, M. and Gooch, M., 2011. Conceptualising, evaluating and reporting social resilience in vulnerable regional and remote communities facing climate change in tropical Queensland. Marine and Tropical Sciences Research Facility (MTSRF) Transition Project Final Report. Reef and Rainforest Research Centre Limited, Cairns, pp. 42-48.

Davies, J., Campbell, D., Campbell, M., Douglas, J., Hueneke, H., LaFlamme, M., Pearson, D., Preuss, K., Walker, J., Walsh, F., 2011. Attention to four key principles can promote health outcomes from desert Aboriginal land management. The Rangeland Journal 33, 417–431.

Davies, J., Campbell, D., Campbell, M., Douglas, J., Hueneke, H., LaFlamme, M., Preuss, K., Walker, J., Walsh, F., 2010. Livelihoods inLand: Promoting health and wellbeing outcomes from desert Aboriginal land management (DKCRC Report No. 78). Ninti One Limited, Alice Springs.

Department of Sustainability and Environment, 2008. Landcare in Victoria: Community participation and perceptions research (Final Report). Department of Sustainability and Environment, East Melbourne, Victoria.

De Hayr B, 2012. Health of the Landcare Movement survey results, National Landcare Facilitator, Australian Government.

Department of Agriculture, 2007. Partners in sustainable production - working together for the benefit of future generations. National Landcare Program, Barton ACT.

Department of Agriculture, 2010. Australian Framework for Landcare, published by the Australian Landcare Council Secretariat.

Department of Agriculture, 2012a. Australian Landcare Council. <http://www.daff.gov.au/natural-resources/landcare/council>.

Department of Agriculture, 2012b. The Landcare approach in Australia. <http://www.daff.gov.au/natural-resources/landcare>.

De Vries, S., Verheij, R.A., Groenewegen, P.P., Spreeuwenberg, P., 2003. Natural environments-healthy environments? An exploratory analysis of the relationship between greenspace and health. Environment and planning A 35, 1717–1732.

Dietz, T., Stern, P.C. (Eds.), 2002. New Tools for Environmental Protection: Education, Information, and Voluntary Measures. The National Academies Press, Washington, D.C.

Dyack, B., Greiner, R., 2006. Natural resource management and Indigenous well being, in: 50th Annual Conference of the Australian Agricultural and Resource Economics Society. Sydney, NSW, p. 31.

Ecker, S., Kancans, R. and Thompson, L. (2011). Drivers of practice change in land management in Australian agriculture: preliminary national survey results. Science and Economic Insights. Australian Bureau of Agricultural and Resource Economics and Sciences Land Use Issue 2.1

Edmonson, R., 2010. Reviewing Landcare.

Emtage, N., Herbohn, J., Harrison, S., 2006. Landholder typologies used in the development of Natural Resource Management programs in Australia–a review. Australasian Journal of Environmental Management 13, 79–94.

Everingham, J., Cheshire, L., Lawrence, G., 2006. Regional renaissance? New forms of governance in nonmetropolitan Australia. Environment and Planning C 24, 139.

Falk, I., Harrison, L., 1998. Community learning and social capital: "just having a little chat". Journal of Vocational Education and Training 50, 609–627.

FAO 2009a. Anchoring Agriculture within a Copenhagen Agreement. A Policy brief for UNFCCC parties by FAO.

FAO 2009b. Enabling Agriculture to contribute to climate change mitigation. A submission by the Food and Agriculture Organization of the United Nations.

FAO 2009c. Food security and agricultural mitigation in developing countries: Options for Capturing Synergies. Rome, Italy.

FAO 2009d. Harvesting agriculture's multiple benefits: Mitigation, Adaptation, Development and Food Security. FAO Policy Brief. Rome, Italy.

Ganesharajah, C., 2009. Indigenous health and wellbeing: The importance of country (Native title Research Report No. 1/2009). Australian Institute of Aboriginal and Torres Strait Islander Studies, Canberra, ACT.

Garnett, S.T., Sithole, B., 2007. Sustainable northern landscapes and the nexus with Indigenous health: Healthy country, healthy people ( No. NTU7). Land & Water Australia, Canberra, ACT.

Garnett, S.T., Sithole, B., Whitehead, P.J., Burgess, C.P., Johnston, F.H., Lea, T., 2009. Healthy country, healthy people: Policy implications of links between Indigenous human health and environmental condition in tropical australia. Australian Journal of Public Administration 68, 53–66.

Gill, N., 2004. Politics within and without — the origins and development of a rangelands Landcare group. Australian Geographical Studies 42, 135–151.

Gleeson, T., Heilbron, S., Hudson, B., Douglas, J., 2006. Capturing market and other benefits from improved land management (Report for National Heritage Trust). Australian Landcare Management System, South Brisbane, Queensland.

Gooch, M., 2003. A sense of place: Ecological identity as a driver for catchment volunteering. Australian Journal on Volunteering 8, 23–32.

Gooch, M.J., 2004. Voices of the volunteers: an exploration of the influences that volunteer experiences have on the resilience and sustainability of catchment groups in coastal queensland (PhD Thesis).

Gordon, L., Folke, C., 2000. Ecohydrological landscape management for human well-being. Water international 25, 178–184.

Gray, I., Lawrence, G., 2001. Neoliberalism, individualism and prospects for regional renewal. Rural Society 11, 283–298.

Griffin NRM, URS Australia Pty Ltd, 2001. An evaluation of investment in Landcare support projects (Final report for Agriculture, Fisheries and Forestry Australia). URS Australia Pty Ltd and Griffin NRM.

Gunningham, N., 2002. Reconfiguring Environmental Regulation: The Future Public Policy Agenda. N. Gunningham & D. Sinclair ds., Leaders and Laggards: Ne t Generation nvironmental Regulation pp.

Gunningham, N., Kagan, R.A., Thornton, D., 2004. Social license and environmental protection: why businesses go beyond compliance. Law & Social Inquiry 29, 307–341.

Gunningham, N., Sinclair, D., 1998. Designing smart regulation, in: Economic Aspects of Environmental Compliance Assurance. OECD Global Forum on Sustainable Development.

Hamilton, C., 1995. The economic language of Landcare (Background Paper No. 3). The Australia Institute, Canberra, ACT.

Hawthorne, M., Alabaster, T., 1999. Citizen 2000: development of a model of environmental citizenship. Global Environmental Change 9, 25–43.

Hegney, D., Ross, H., Baker, P., Buikstra, E., King, C., McLachlan, K., Rogers-Clark, C., Stallard, L., Watson-Luke, A., 2008. Identification of personal and community resilience that enhance psychological wellness: A Stanthorpe study. Australian Research Council, Canberra, ACT.

Hegney, D., Ross, H., Baker, P., Rogers-Clark, C., King, C., Buikstra, E., Watson-Luke, A., McLachlan, K., Stallard, L., 2008b. Building resilience in rural communities (Toolkit). The University of Queensland and University of Southern Queensland, Toowoomba, Queensland.

Hill, R., Williams, L., Lane, M., Robinson, C., Taylor, B., 2009. Indigenous natural resource management: overcoming marginalisation produced in Australia's current NRM model. Contested country: local and regional environmental management in Australia 161–178.

Hogan, L., Cumming, B., 1997. More than a question of numbers: Working with women and people from a non-English speaking background toward total catchment participation (Report of the Landcare Participation Project). Department of Primary Industries and Energy, and the Department of Natural Resources and Environment, Canberra, ACT.

Horvath, D. (Ed.), 2001. NSW Decade of Landcare Evaluation. NSW Department of Land and Water Conservation, Sydney, NSW.

Horwitz, P., Lindsay, M., O'Connor, M., 2001. Biodiversity, endemism, sense of place, and public health: Inter-relationships for Australian inland aquatic systems. Ecosystem Health 7, 253–265.

Jennings J., Packham R., Woodside and D. Eds, 2010. 'Shaping Change: Natural Resource Management, Agriculture and the Role of Extension.' Australasia-Pacific Extension Network (APEN), Australia.

Johnston, F., Burgess, P., Bowman, D.M.J.S., 2007. Chapter 15 - A case for Indigenous natural resource management and health, in: Luckert, M.K., Campbell, B.M., Gorman, J.T., Garnett, S.T. (Eds.), Investing in Indigenous Natural Resource Management. Charles Darwin University Press, Darwin, NT.

Kellert, S.R., Derr, V., 1998. A national study of outdoor wilderness experience. Unpublished report. New Haven, CT: Yale University.

Kingsley, J., Townsend, M., Phillips, R., Aldous, D., 2009. "If the land is healthy … it makes the people healthy": The relationship between caring for Country and health for the Yorta Yorta Nation, Boonwurrung and Bangerang Tribes. Health & Place 15, 291–299.

Koss, R.S., Kingsley, J.Y., 2010. Volunteer health and emotional wellbeing in marine protected areas. Ocean & Coastal Management 53, 447–453.

Land & Water Australia, 2003. National Landcare Program Review. Land & Water Australia.

Landcare Network Victoria, Landcare Victoria, 2004. Making community Landcare even stronger: Victorian Landcare Forum report. Presented at the 2004 Victorian Landcare Forum, Victorian Government Department of Sustainability and Environment, Bendigo, Victoria, p. 68.

Landcare Network Victoria, Landcare Victoria, 2005. Making community Landcare even stronger: Stories of success. Presented at the 2004 Victorian Landcare Forum, Victorian Government Department of Sustainability and Environment, Bendigo, Victoria, p. 68.

Landcare Online, 2012. <http://www.landcareonline.com.au>.

Landcare Victoria, Department of Sustainability and Environment, 2008a. Shaping our future: Proceedings. Presented at the 2008 Victorian Landcare Forum, Landcare Victoria, Creswick, Victoria, p. 21.

Landcare Victoria, Department of Sustainability and Environment, 2008b. Shaping our future: Proceedings. Presented at the 2008 Victorian Landcare Forum, Landcare Victoria, Creswick, Victoria, p. 28.

Landcare Victoria, Department of Sustainability and Environment, 2008c. Shaping our future: Stories. Presented at the 2008 Victorian Landcare Forum, Landcare Victoria, Creswick, Victoria, p. 42.

Landcare Victoria, Department of Sustainability and Environment, 2008d. Shaping our future: Stories. Presented at the 2008 Victorian Landcare Forum, Landcare Victoria, Creswick, Victoria, p. 34.

Lane, M.B., 2002. Buying back and caring for country: institutional arrangements and possibilities for indigenous lands management in Australia. Society &Natural Resources 15, 827–846.

Lauber, T.B., Decker, D.J., Knuth, B.A., 2008. Social networks and community-based natural resource management. Environmental management 42, 677–687.

Lloyd, D.L., Johnson, B., O'Brien, S.M., Lawrence, D.N., 2009. Action learning in partnership with Landcare and catchment management groups to support increased pasture sowings in southern inland Queensland. Animal Production Science 49, 907.

Lockie, S.D., 1996. Sociocultural dynamics and the development of the landcare movement in Australia (PhD Thesis).

Lockie, S., 1998. Landcare in Australia: Cultural transformation in the management of rural environments. Culture & Agriculture 20, 21–29.

Lockie, S., 2001. Chapter 20 - Community development management? Landcare in Australia, in: Lockie, S., Bourke, L. (Eds.), Rurality Bites. Pluto Press, Annandale, NSW.

Lockie, S., 2003. Conditions for building social capital and community well-being through plantation forestry. Australian Forestry 66, 24–29.

Love, C., 2012. Evolution of Landcare in Australia: In the context of Australian Government natural resource management policy and programs (Perspectives from the former National Landcare Facilitator). Australian Landcare Council Secretariat.

Lovell, C., Mandondo, A., Moriarty, P., 2003. The question of scale in integrated natural resource management. Integrated natural resource management. Linking productivity, the environment and development. CABI, Wallingford 109–136.

MacGregor, S., Pardoe, S., Dobson, A., Bell, D., 2005a. Environmental citizenship: the Goodenough primer (summary report of an interdisciplinary seminar series) (Summary Report). The Open University, Milton Keynes, UK.

MacGregor, S., Pardoe, S., Dobson, A., Bell, D., 2005b. Environmental citizenship: the Goodenough primer (summary report of an interdisciplinary seminar series) (Summary Report). The Open University, Milton Keynes, UK.

MacGregor, S., Pardoe, S., Dobson, A., Bell, D., 2005c. Environmental Citizenship: the Goodenough primer (Final Seminar). Goodenough College, London, UK.

Maller, C., Townsend, M., Brown, P., St Leger, L., 2002. Healthy parks healthy people: The health benefits of contact with nature in a park context. Burwood (Australia): Faculty of Health and Behavioural Sciences, Deakin University.

Maller, C., Townsend, M., St Leger, L., Henderson-Wilson, C., Pryor, A., Prosser, L., Moore, M., 2008. Healthy parks, healthy people. The health benefits of contact with nature in a park context. A review of relevant literature, 2nd edition. Melbourne, Victoria.

McDermott, R., O'Dea, K., Rowley, K., Knight, S., Burgess, P., 2008. Beneficial impact of the Homelands Movement on health outcomes in central Australian Aborigines. Australian and New Zealand Journal of Public Health 22, 653–658.

McTernan, R., Scully, M.-A., 2010. Finding common ground: A review of Indigenous engagement in Landcare in Victoria. Victorian Government Department of Sustainability and Environment, Melbourne, Victoria.

Metcalfe, J (2003). Landcare in the Philippines. ACIAR Monograph 112.

Mobbs, C., Dovers, S., 1999. Social, economic, legal, policy and institutional R&D for natural resource management: Issues and directions for LWRRDC. Occasional paper 1, 99.

Moore, S.A., 2005. Regional delivery of natural resource management in Australia: Is it democratic and does it matter. Participation and governance in regional development: global trends in an Australian context 121–136.

Moore, S.A., Jennings, S.F., Tacey, W.H., 2001. Achieving sustainable natural resource management outcomes on the ground: the key elements of stakeholder involvement. Australasian Journal of Environmental Management 8, 91–98.

Moore, S.A., Rockloff, S.F., 2006. Organizing regionally for natural resource management in Australia: reflections on agency and government. Journal of Environmental Policy & Planning 8, 259–277.

Mues, C., Chapman, L., Van Hilst, R., 1998. Promoting improved land management practices on Australian farms: A survey of landcare and land management related programs (Research report No. 98.4), Landcare. Australian Bureau of Agricultural and Resource Economics, Canberra, ACT.

Mullen, J.D., 2001. An economic perspective on land degradation issues (Economic Research Report No. 9). NSW Agriculture, Orange, NSW.

National Landcare Facilitator Project, 2007. Community Engagement & Involvement in Australian Government Progammes, Key report in the National Landcare Facilitator 2006-07 Annual report.

National Landcare Network, 2012. <http://www.nln.org.au/charter>.

Newby, J.C., Cramb, R.A., 2009. Living on the margin: Assessing the economic impacts of Landcare in the Philippine uplands, in: Proceedings of the 53rd Annual Conference. Presented at the Australian Agricultural and Resource Economics Society, Australian Agricultural and Resource Economics Society, Cairns, Queensland, p. 29.

Newby, J.C. and Cramb, R.A. (2011). Economic impacts of conservation farming in a marginal environment: the case of Landcare in the Philippines. International Journal of Agricultural Sustainability, 9 3: 456-470.

Nicholson, C., Knight, A., 2003. Auditing 10 years of Landcare in the Woady Yaloak catchment: How are we progressing along the triple bottom line? Presented at the National Landcare Conference, Darwin, NT, p. 7.

Pannell, D.J., 2001. Dryland salinity: economic, scientific, social and policy dimensions. Australian Journal of Agricultural and Resource Economics 45, 517–546.

Parkes, M., Panelli, R., 2001. Integrating catchment ecosystems and community health: the value of participatory action research. Ecosystem Health 7, 85–106.

Paton, S., Curtis, A., McDonald, G., Woods, M., 2004. Regional natural resource management: is it sustainable. Australasian Journal of Environmental Management 11, 259–267.

Pepperdine, S., Ewing, S., 2001. Integrating social sustainability considerations into natural resource management. Lockie, S. Higgins, V. and Lawrence, G.(eds). Environment, Society and Natural Resource Management: Theoretical Perspectives from Australasia and the Americas 70–83.

Petheram, R.J., Clark, R.A., 1998. Farming systems research: relevance to Australia. Animal Production Science 38, 101–115.

PIRSA Sustainable Resources Group, 2000. School and community cooperative landcare projects: Best practice manual. Primary Industries and Resources South Australia, Adelaide, SA.

Pretty, G., Bishop, B., Fisher, A., Sonn, C., 2007. Psychological sense of community and its relevance to well-being and everyday life in Australia. The Australian Community Psychologist 19, 6–25.

Pretty, J., 2003. Social capital and the collective management of resources. Science 302, 1912–1914.

Pretty, J., Smith, D., 2004. Social capital in biodiversity conservation and management. Conservation Biology 18, 631–638.

Pretty, J., Ward, H., 2001. Social capital and the environment. World development 29, 209–227.

Price-Robertson, R. and Knight, K., 2012. Natural disasters and community resilience: a framework for support, Child family Community Australia, Australian Government, Canberra, ACT.

Prior, J., 1996. `From technology transfer to community development: the policy implications of the Australian Landcare movement', in S. Sombatpanit, M.A. Zobisch, D.W. Sanders and M.G. Cook (eds), Soil Conservation Extension: from Concepts to Adoption, International Board for Soil Research and Management,Thailand, pp. 77-86.

Prior, J., 2002. Assessment of Past Experience and Possible Future Directions for LandCare in South Africa – Second Report (South Africa – Australia Institutional Strengthening of the Department of Agriculture Program). Report for AusAID and the Government of South Africa.

Putnam, R.D., 1993. The prosperous community. The American prospect 4, 35–42.

Quealy, J., 1998. Landcare: Rural Landcare. Landcare New South Wales, Sydney, NSW.

Raymond, C.M., Bryan, B.A., MacDonald, D.H., Cast, A., Strathearn, S., Grandgirard, A., Kalivas, T., 2009. Mapping community values for natural capital and ecosystem services. Ecological Economics 68, 1301–1315.

Ritchie, H., 1995. Landcare in the deregulated rural economy of New Zealand. Rural Society 5, 67–75.

Ross, H., Cuthill, M., Maclean, K. Jansen, D. and Witt, B., 2010. Understanding, Enhancing and Managing for Social Resilience at the Regional Scale: Opportunities in North Queensland. Report to the Marine and Tropical Sciences Research Facility. Reef and Rainforest Research Centre Limited, Cairns, p. 186.

Roughley, R., Williams, S., 2009. The engagement of Indigenous Australians in natural resource management: Key findings and outcomes from Land & Water Australia and the broader literature (Final report to Land & Water Australia). Land & Water Australia, Canberra, ACT.

Sobels, J., Curtis, A., 2001a. Landcare networks: Establishing viable local organisations in rural Australia. Natural Resource Management 4, 22–30.

Sobels, J., Curtis, A., 2001b. Second generation Landcare support in West Gippsland (report for Landcare Victoria No. 156). Johnstone Centre, Charles Sturt University, Albury, NSW.

Sobels, J., Curtis, A., Lockie, S., 2001. The role of Landcare group networks in rural Australia: Exploring the contribution of social capital. Journal of Rural Studies 17, 265–276.

Speldewinde, P.C., Cook, A., Davies, P., Weinstein, P., 2009. A relationship between environmental degradation and mental health in rural Western Australia. Health & Place 15, 880–887.

Speldewinde, P.C., Cook, A., Davies, P., Weinstein, P., 2011. The hidden health burden of environmental degradation: disease comorbidities and dryland salinity. Ecohealth 8, 82–92.

Stern, P.C., 2000a. Toward a coherent theory of environmentally significant behaviour. Journal of Social Issues 56, 407–424.

Stern, P.C., 2000b. New environmental theories: Toward a coherent theory of environmentally significant behaviour. Journal of social issues 56, 407–424.

Townsend, M., Weerasuriya, R., 2010. Beyond blue to green: The benefits of contact with nature for mental health and well-being. Beyond Blue Limited, Melbourne, Victoria.

Toyne, P., Farley, R., 2000. The decade of Landcare: Looking backward - looking forward (Discussion Paper No. 30). The Australia Institute.

Vanclay, F., 2004. Social principles for agricultural extension to assist in the promotion of natural resource management. Animal Production Science 44, 213–222.

Vanclay, F.M., Lockie, S. (Eds.), 2000. Critical Landcare, Key papers series. Centre for Rural Social Research, Charles Sturt University, Wagga Wagga, NSW.

Walker, B. and Salt, D., 2012. Resilience Practice: Building Capacity to Absorb Disturbance and Maintain Function. Island Press, Washington DC.

Walker, R.H., 2000. The National Landcare Program: What has it changed? (A compilation of evaluations of the National Landcare Program 1992-1998). Department of Agriculture, Fisheries and Forestry, Canberra, ACT.

Webb, T., Cary, J., 2005. Social capital and natural resource management: An application to Landcare. Rural Society 15, 119–131.

Webb, T.J., Cary, J., 2009. Australia: Social capital and natural resource management - the Australian Landcare movement, in: Rivera, W., Alex, G. (Eds.), Revitalization Within Public Sector Services: Case Studies of International Initiatives, Agriculture and Rural Development Discussion Paper 11: Extension Reform for Rural Development. The World Bank, Washington, D.C., pp. 5–11.

Weir, J.K., Stacey, C., Youngetob, K., 2011. The benefits of caring for country (Report for the Department of Sustainability, Environment, Water, Population and Communities). Australian Institute of Aboriginal and Torres Strait Islander Studies, Canberra, ACT.

Westermann, O., Ashby, J., Pretty, J., 2005. Gender and social capital: The importance of gender differences for the maturity and effectiveness of natural resource management groups. World Development 33, 1783–1799.

Wilson, G.A., 2004. The Australian Landcare movement: Towards "post-productivist" rural governance? Journal of Rural Studies 20, 461–484.

Woodhill, J., 1992. Landcare in NSW, taking the next step! Final report and recommendations for the development of Landcare in NSW from the 1990/91 (Final report for the National Soil Conservation Program). Department of Conservation and Land Management, Chatswood, NSW.

Woodhill, J., 2010. Sustainability, Social Learning and the Democratic Imperative: Lessons from the Australian Landcare Movement, in: Blackmore, C. (Ed.), Social Learning Systems and Communities of Practice. Springer London, pp. 57–72.

Youl, R. (Ed.), 2006. Landcare in Victoria: How Landcare Helped People, Government and Business Work Together in Victoria - a Collection. R. Youl, South Melbourne, Victoria.

Youl, R., Marriott, S., Nabben, T., 2006. Landcare in Australia: Founded on Local Action, 5th ed. SILC, Melbourne, Victoria.

# Appendix B EEA: A synthesis of supporting evidence

ENVIRONMENTAL EVIDENCE AUSTRALIA

## Multiple Benefits of NRM and Landcare: A synthesis of supporting evidence

**November 2012**

**Document Status**

| **Version** | **Primary Author** | **Reviewed and Approved for Issues - Name** | **Reviewed and Approved for Issues - Position** | **Reviewed and Approved for Issues - Date** |
|---|---|---|---|---|
| 1.0 | Cristina Venables | Rob Richards | Director | 12 August 2012 |
| 2.0 | Rob Richards | Mat Silver | Director | 13 December 2012 |

COPYRIGHT: The concepts and information contained in this document are the property of Environmental Evidence Australia Pty Ltd. Use or copying of this document in whole or in part without the written permission of Environmental Evidence Australia constitutes an infringement of copyright.

LIMITATION: This report has been prepared on behalf of and for the exclusive use of Environmental Evidence Australia Ltd's client, and is subject to and issued in connection with the provisions of the agreement between Environmental Evidence Australia and its client. Environmental Evidence Australia accepts no liability or responsibility whatsoever for or in respect of any use of or reliance upon this report by any third party

## Introduction

There is a plethora of Landcare stories of success that have been captured through numerous evaluations (Curtis and De Lacy, 1995; Curtis et al., 1993; Department of Agriculture, 2003; Edmonson, 2010; Horvath, 2001; Woodhill, 1992; Youl, 2006), forums and workshops (Landcare Victoria and Department of Sustainability and Environment, 2008a, 2008b, 2008c, 2008d), interviews and studies. Each case study or story is a small slice through the history of Landcare. Each success and failure is influenced by the local context, individuals, communities and broader Landcare framework.

Landcare's success can be expressed in ways beyond the number of volunteers and the amount of on-ground work. This has been proven through a diverse range of socio-economical benefits in addition to the foreseen natural resource management outcomes. The evidence presented in this report aims to shed light on a few of the more common key benefits beyond the direct natural resource management outcomes that are reported in the literature. The categories used are not discrete but instead are heavily integrated and interdependent. It is, for example difficult to discuss mental health outcomes without considering these to be also factors of individual resilience. Similarly there is a strong interdependency between social capital and education and awareness as there is between education and awareness with economic benefits.

# Table of Contents

**Introduction ........................................................................................ 2**

**7. Evidence synthesis ................................................................................ 4**
7.1 Education, awareness and practice change ................................................ 4
7.2 Social capital ...........................................................................14
7.3 Economic ...............................................................................23
7.4 Health and wellbeing ....................................................................30
7.5 Resilience...............................................................................37

**Appendices ........................................................................................ 43**
8. Appendix 1: Literature Cited or References ................................................43

## 7. Evidence synthesis

The synthesis of evidence is presented according to Landcare benefit categories agreed on with the GHD project team. These are:

- Education, awareness and practice change
- Social Capital
- Economic
- Health and well being
- Resilience

A non-systematic search was conducted in order to synthesize evidence on the health and wellbeing benefits of Landcare and natural resource management (NRM) activities.

All cited evidence is provided in an electronic Zotero evidence base to enable future access or further enquiry.

### 7.1 Education, awareness and practice change

In one of his earlier papers, Campbell stated that "many committed, far-sighted people are involved in Landcare. They are gaining intellectual stimulation, exciting new knowledge and the satisfaction of doing something constructive in their own district and of influencing others" (Campbell, 1992 p.1). This predictive statement of confidence has proved to have had good foresight, rather than over optimism in the twenty years since it was made.

Landcare is widely recognised as a movement that has fundamentally shifted the perceptions of land stewardship through increased awareness and knowledge of the landscape and the relationship of people to that landscape. Landcare has provided highly effective coordinated opportunities at a range of scales for experimentation, learning, increased awareness, observation, and skill development (Curtis and Sample, 2010; Curtis et al., 2008, 2000). ABARE surveys show that as many as 50% of all farmers have utilised Landcare groups for information regarding farm management, demonstrating that Landcare has been a major catalyst for practice change and increased adaptive management (Department of Agriculture, 2003).

As the first Landcare facilitator in Australia and a passionate visionary for Landcare, Andrew Campbell states: "The personal and direct involvement of people in gathering and interpreting information about the health of the land around them as an everyday activity seems to be inextricably linked with an accompanying ethic—of land stewardship, and respect for and humility towards nature. Such an ethic both underpins and is invigorated by contact with, and understanding of, the natural world. Such an understanding comes with direct involvement in gathering and recording information about vital signs such as water quality, the extent and status of indicator species, problems such as soil salinity and erosion and so on" (Campbell, 1995 p.5).

Of key contemporary relevance is the role that Landcare has played in enhancing state agricultural extension services and filling the void left from economic rationalism in the funding of government extension activities, and the need for those services to seek

alternative forms of funding and more efficient methods of technology transfer. The Landcare model can be an effective mechanism to facilitate the transfer of knowledge in partial response to declining extension funding (Cary and Webb, 2000; Vanclay and Lockie, 2000; Walker, 2000).

The following passages provide a synthesis of evidence of some key areas within the education, awareness and practice change benefits resulting from Landcare in key areas defined by the literature.

**Awareness raising**

A range of reviews have found that Landcare funding has been effective in raising awareness and as valuable infrastructure for delivering information (Cary and Webb, 2001; Curtis, 1999; Walker, 2000) and changing behaviour (Curtis and De Lacy, 1996a; Curtis, 1995a; Walker, 2000).

There is strong evidence that Landcare participation leads to significantly higher levels of awareness and concern about a range of land and water degradation issues (Curtis and De Lacy, 1996a). They found that Landcare participants in North East Victoria were significantly more likely to be aware of dryland salinity, soil acidity, tree decline, and soil compaction. Landcare participants were also significantly more concerned about the economic, social and environmental impact of land and water degradation issues (Curtis and De Lacy, 1996a; Curtis et al., 2008). The authors also report in a later paper that "at the same time, all landholders in Landcare areas were significantly more likely to report awareness of the less obvious issues such as dryland salinity, soil acidity and soil compaction than those respondents from non-Landcare areas that had similar land and water degradation issues" (Curtis and De Lacy, 1998 p. 621). Additionally, non-Landcare participants acknowledge the wealth of knowledge about land and water degradation and sustainable farming practices that Landcare groups hold (Curtis et al., 2008).

It is reported that an increased awareness of Landcare is evidenced by growth in the Landcare movement and the widespread involvement in community environmental monitoring (Sobels and Curtis, 2001).

**Practice change – where the rubber hits the road**

Curtis, 2003 reports that there is strong evidence that participation in landcare activities is a precursor to the accomplishment of program outcomes. An example of this is that groups involved in field days and demonstration sites undertake significantly higher amounts of on-ground work related to tree planting, fencing to manage stock access to waterways, and pest animal and weed control (Curtis and De Lacy, 1996a; Curtis, 1995b; Curtis et al, 2000; Curtis et al, 2008). Additionally, through educational activities, Landcare has encouraged farmers to appraise problems more holistically, which often leads to new methods for tackling these issues (Lockie, 1998; Youl et al., 2006).

Similarly Mues et al., 1998 reports that Landcare members were at least twice as likely as non-members to have participated in some Landcare group workshops and field days, industry grower groups, property management planning activities.

Curtis, 2003 draws on other papers to provide more examples of this including:

- A higher proportion of group members engaged in property planning can be shown to be linked to groups undertaking significantly higher amounts of on-ground work (Curtis and De Lacy, 1995)

- Groups involved in establishing annual priorities and developing catchment plans accomplish significantly higher amounts of on-ground work.

- Landcare members involved in field days and demonstration sites undertake significantly higher amounts of perennial pasture establishment than members who are not involved in these activities (Curtis and De Lacy 1996).

In a recent study in the Wimmera region of Victoria, Curtis, 2003 reports that individuals involved in short courses relevant to property management, including those run by Landcare groups, were significantly more likely to adopt seven of ten recommended sustainable farming practices. In addition, the Department of Agriculture review of the National Landcare Program postulates that the development of trust through learning with peers, such as in Landcare groups, facilitates acceptance and adoption of new farming practices and management techniques (Department of Agriculture, 2003).

**Multigenerational reach**

In a recent paper, Love, 2012 suggests that Landcare has supported intergenerational learning through group corporate knowledge, family knowledge and school activities. Landcare has been an effective tool for reaching school children. Evidence suggests that when the Landcare ethic and practices are embedded in school curricula, not only do children take these onboard and run with them, but they also influence their parents, other family members and other children. Important in this concept is the realisation that both the children and their families may belong to sectors of the community that Landcare has traditionally found hard to reach (Landcare Victoria and Department of Sustainability and Environment, 2008). As reported in other sections, Landcare networks are increasingly taking a leadership role, gaining a wider reach and collective resources greater than any single group. They are also well positioned to influence the community on a greater geographical scale as well as engage with the private sector, industry, schools and local government to an unprecedented extent (Landcare Victoria and Department of Sustainability and Environment, 2008).

**Improved knowledge**

Curtis, 2003 draws on Curtis and De Lacy, 1996b to report that surveys have consistently identified that Landcare participants as showing significantly higher levels of knowledge of land and water degradation processes and sustainable farming practices recommended to mitigate or prevent these issues. Research by Curtis shows that Landcare participants in North East Victoria were significantly more likely to report high to very high knowledge of processes leading to soil erosion; processes leading to soil acidification; the impact of tree removal on water tables; how to collect samples for soil tests; how to develop property management plans using land classes; and how to establish perennial pastures (Curtis, 2003). Community monitoring activities have also been shown to result in the development of new technology and equipment, demonstrating that Landcare monitoring groups can be an important source for innovation (Campbell, 1995).

National Landcare funding during the Decade of Landcare produced new information on the level of understanding of landscape processes, resources assessments, national scale data collections and standards, decision support programs and the interaction between agricultural systems, natural systems, land and water resources processes (Walker, 2000). It has been shown that Landcare promotes learning between rural landholders by engaging them in activities with each other, providing them with the opportunity to learn with their peers, to learn by doing, and to reflect on shared experiences (Curtis and Sample, 2010).

Research undertaken by Sobels et al in 2006 of 242 South Australian Landcare groups revealed that Landcare had:

- increased understanding of what biodiversity conservation involves (86% said "some/much/high level of success")
- contributed to changed land management practices (60%)
- increased understanding of what sustainable farming involves (59%).

In other studies conducted Coree Consulting, 2003 reported that over 60 per cent of Landcare members reported learning more about the causes of degradation, how to recognise it and techniques to monitor the condition of land and water (which are the main elements of the term "Land Literacy") (Campbell, 1995). In the same study 74 per cent reported learning more about farm practices to treat or avoid degradation (Coree Consulting Pty Ltd, 2003). Furthermore, Mues found that 56 per cent of dairy and broadacre farmers used Landcare groups as a source of information for farm management and techniques (Mues et al., 1998). This is higher than Landcare membership, which means that community members outside of Landcare groups are also benefitting from their knowledge.

**Scales of change**

Landcare has helped natural resource managers recognise the need for management at greater spatial scales and for integrated natural resource management. Landcare has encouraged and supported the establishment of institutional arrangements to enable integration to occur (Walker, 2000).

The ability of the Landcare framework to self organise and develop more sophisticated networks has enhanced the opportunities of individual groups and also enabled Landcare to participate in planning and management at larger scales. Landcare groups have acted as a forum for discussions contributing to the development of regionally relevant management practices (Curtis and Cooke, 2006).

Landcare groups in many instances have progressed from focusing on single issues and on small area projects to developing a bigger picture with regional or catchment-wide plans. Landcare groups are also recognising the need to involve more fully the urban community, local government, rural industry bodies and public land managers as significant stakeholders in natural resource management (Department of Agriculture, 2003).

**Continuous learning**

The Landcare model provides a sound basis for effective continuous learning. As reported in previous sections, Landcare builds knowledge and understanding that increase participant competency and strengthens capacity for adaptive management processes, as well as providing appropriate institutional structures for ongoing community representation. This ensures that information and understanding from adaptive management processes are communicated and retained within local communities and provide participants with a base so that they engage in planning processes as effective partners (Curtis and Lockwood, 2000).

It is reported that Landcare leaders encourage open-mindedness and an awareness of the diverse reactions to changes throughout the courses of actions when implementing

new policies and practices. Catacutan et al., 2009 states that these policies and practices themselves must also be adaptive to emerging changes in the socioeconomic and environmental dynamics, constituting the triple bottom line in environmental decision-making. The author also states that nationwide rules are avoided and flexibility is encouraged for programmes that recognize that most Landcare actors are volunteers and cannot always meet tight deadlines.

Moving away from development bureaucracies in favour of organizations concentrated on process and capacity building, Landcare stimulates continuous learning as a guiding principle. Using champion individuals, Landcare demonstrates that capacity building is achieved through even modest resources in a natural process of assimilation and commitment building (Catacutan et al., 2009). Emphasis on livelihood improvement in Landcare projects is combined with continuous education and value transformation. This orientation on learning combined with the willingness to experiment, potentially fail and draw lessons is the key to achieving Landcare goals (Catacutan et al., 2009).

Another area that integrates continuous learning and new governance models (see section on Social Capital) is the role of Landcare in the growing emphasis on environmental citizenship and other notions of more self-regulating approaches to tackling environmental issues. MacGregor et al., 2005 suggests that environmental citizenship is an important part of the shift towards governance, rather than just being governed, in environmental policy and politics. In the past, governments have overwhelmingly used fiscal sticks and carrots or economic instruments as a mechanism for moving people towards more sustainable behaviour (MacGregor et al., 2005). Land stewardship and environmental ethics seem to be linked with the action of gathering and interpreting information regarding environmental health, as promoted by Landcare, and can lead to changes in social norms and awareness of their ecological footprint (Campbell, 1995). This shift towards more self regulating governance is a fundamental plank of environmental citizenship and Landcare.

The literature reports on a change in the nature of environmental policy over the last thirty years from predominantly "command and control" and market based approaches to more focus on education, provision and information and voluntary approaches (Dietz and Stern, 2002). This fundamental shift in thinking is the basis of frameworks proposed by several authors regarding new governance, regulatory reform and understanding of environmental behaviour (Gunningham and Sinclair, 1998; Hawthorne and Alabaster, 1999; P. C. Stern, 2000; Gunningham, 2002; Gunningham et al., 2004). These frameworks are all important in understanding and in operationally defining the broad church of environmental citizenship and the suitability of the Landcare model in contributing towards new governance structures in environmental management.

#### 7.1.1 Citations

Altman, J.C., Whitehead, P.J., 2003. Caring for country and sustainable Indigenous development: opportunities, constraints and innovation (CAEPR Working Paper No. 20). Centre for Aboriginal Economic Policy Research.

Beilin, R., Reichelt, N., 2010. Community Landcare: a key player in building social-ecological resilience networks? The State of Victoria Department of Sustainability and Environment, Melbourne.

Brown, V., 1997. Landcare in Australia: talking local sustainability in policy, practice and place (RDFN Paper No. 20e). Rural Development Forestry Network, London, UK.

Buikstra, E., Ross, H., King, C.A., Baker, P.G., Hegney, D., McLachlan, K., Rogers-Clark, C., 2010. The components of resilience - perceptions of an Australian rural community. Journal of Community Psychology 38, 975–991.

Campbell, A., 1992. Taking the long view in tough times: Landcare in Australia (Third Annual Report), National Landcare Facilitator. National Soil Conservation Program.

Campbell, A., 1995a. Land literacy in Australia. Journal of Soil and Water Conservation 126–131.

Campbell, C.A., 1995b. Landcare: Participative Australian approaches to inquiry and learning for sustainability. Journal of Soil and Water Conservation 50, 125–131.

Campbell, C.A., 2009. It's time to renew Landcare. Agricultural Science 30–33.

Cary, J., Webb, T., 2000. Community landcare, the National Landcare Program and the landcare movement: the social dimensions of landcare. Bureau of Rural Science, Canberra, ACT.

Cary, J., Webb, T., 2001. Landcare in Australia: Community participation and land management. Journal of Soil and Water Conservation 56, 274–278.

Catacutan, D., Neely, C., Johnson, M., Youl, R. (Eds.), 2009. Landcare: Local Action - Global Progress. World Agroforestry Centre, Nairobi, Kenya.

Colliver, R., 2011. Community-based governance in social-ecological systems: An inquiry into the marginalisation of Landcare in Victoria, Australia (PhD of Philosophy Thesis).

Compton, E., Hayne, W., Beeton, R.J.S., 2007. Six years in a cement mixer: A landcare journey through changing times. Presented at the International Landcare Conference, Melbourne, Victoria.

Coree Consulting Pty Ltd, 2003. Landcare Literature Review. Coree Consulting Pty Ltd.

Curtis, A., 1995a. Landcare in Australia: A critical review (report No. 33). Charles Sturt University, Albury, NSW.

Curtis, A., 1995b. Landcare in Victoria: The state of play (Report for Landcare Victoria No. 24). Johnstone Centre, Charles Sturt University, Albury, NSW.

Curtis, A., 1999. Landcare in Victoria: Beyond onground work (Report No. 125). Johnstone Centre, Charles Sturt University, Albury, NSW.

Curtis, A., 2003. Reflecting on the Landcare experience: A report based on information held within ABARE and BRS. Bureau of Rural Sciences.

Curtis, A., Britton, A., Sobels, J., 1999. Landcare networks in Australia: State-sponsored participation through local organizations. Journal of Environmental Planning and Management 42, 5–21.

Curtis, A., Cooke, P., 2006. Landcare groups in Victoria: After twenty years (Report to the Australian Government Deparment of Agriculture, Fisheries and Forestry, National Landcare Program, Monitorin and Evaluation Project). Instutute for Land, Water and Society, and the National Landcare Program, Albury, NSW.

Curtis, A., De Lacy, T., 1995. Evaluating landcare groups in Australia: How they facilitate partnerships between agencies, community groups, and researchers. Journal of Soil and Water Conservation January-February, 15–20.

Curtis, A., De Lacy, T., 1996a. Landcare in Australia: Does it make a difference? Journal of Environmental Management 46, 119–137.

Curtis, A., De Lacy, T., 1996b. Landcare in Australia: Beyond the expert farmer. Agriculture and Human Values 13, 20–32.

Curtis, A., De Lacy, T., 1996c. Landcare in Australia: Does it make a difference? Johnstone Centre, Charles Sturt University, Albury, NSW.

Curtis, A., De Lacy, T., 1998. Landcare, stewardship and sustainable agriculture in Australia. Environmental Values 7, 59–78.

Curtis, A., De Lacy, T., Klomp, N., 1993. Asessing the effectiveness of landcare groups: The Victorian experience. Regional Journal of Social Issues 27, 78–91.

Curtis, A., Lockwood, M., 2000. Landcare and catchment management in Australia: Lessons for state-sponsored community participation. Society & Natural Resources 13, 61–73.

Curtis, A., Lucas, D., Nurse, M., Skeen, M., 2008. Achieving NRM outcomes through voluntary action: Lessons from Landcare (Discussion Paper). Charles Sturt University & Victorian Department of Sustainability and Environment, Melbourne, Victoria.

Curtis, A., Sample, R., 2010. CBNRM in Victoria: Contributing to dialogue, learning and action (ILWS Report No. 55). Institute for Land, Water and Society, Charles Sturt University, Albury, NSW.

Curtis, A., Van Nouhuys, M., Robinson, W., MacKay, J., 2000. Exploring Landcare effectiveness using organisational theory. Australian Geographer 31, 349–366.

Department of Agriculture, 2003. Review of the National Landcare Program (Report). Department of Agriculture, Canberra, ACT.

Dietz, T., Stern, P.C. (Eds.), 2002. New Tools for Environmental Protection: Education, Information, and Voluntary Measures. The National Academies Press, Washington, D.C.

Edmonson, R., 2010. Reviewing Landcare.

Garnett, S.T., Sithole, B., Whitehead, P.J., Burgess, C.P., Johnston, F.H., Lea, T., 2009. Healthy country, healthy people: Policy implications of links between Indigenous human health and environmental condition in tropical australia. Australian Journal of Public Administration 68, 53–66.

Gooch, M.J., 2004. Voices of the volunteers: an exploration of the influences that volunteer experiences have on the resilience and sustainability of catchment groups in coastal queensland (PhD Thesis).

Griffin NRM, URS Australia Pty Ltd, 2001. An evaluation of investment in Landcare support projects (Final report for Agriculture, Fisheries and Forestry Australia). URS Australia Pty Ltd and Griffin NRM.

Gunningham, N., 2002. Reconfiguring Environmental Regulation: The Future Public Policy Agenda. N. Gunningham & D. Sinclair ds., Leaders and Laggards: Ne t Generation nvironmental Regulation pp.

Gunningham, N., Kagan, R.A., Thornton, D., 2004. Social license and environmental protection: why businesses go beyond compliance. Law & Social Inquiry 29, 307–341.

Gunningham, N., Sinclair, D., 1998. Designing smart regulation, in: Economic Aspects of Environmental Compliance Assurance. OECD Global Forum on Sustainable Development.

Hamilton, C., 1995. The economic language of Landcare (Background Paper No. 3). The Australia Institute, Canberra, ACT.

Hawthorne, M., Alabaster, T., 1999. Citizen 2000: development of a model of environmental citizenship. Global Environmental Change 9, 25–43.

Hegney, D., Ross, H., Baker, P., Buikstra, E., King, C., McLachlan, K., Rogers-Clark, C., Stallard, L., Watson-Luke, A., 2008. Identification of personal and community resilience that enhance psychological wellness: A Stanthorpe study. Australian Research Council, Canberra, ACT.

Hogan, L., Cumming, B., 1997. More than a question of numbers: Working with women and people from a non-English speaking background toward total catchment participation (Report of the Landcare Participation Project). Department of Primary Industries and Energy, and the Department of Natural Resources and Environment, Canberra, ACT.

Horvath, D. (Ed.), 2001. NSW Decade of Landcare Evaluation. NSW Department of Land and Water Conservation, Sydney, NSW.

Landcare Victoria, Department of Sustainability and Environment, 2008a. Shaping our future: Proceedings. Presented at the 2008 Victorian Landcare Forum, Landcare Victoria, Creswick, Victoria, p. 21.

Landcare Victoria, Department of Sustainability and Environment, 2008b. Shaping our future: Proceedings. Presented at the 2008 Victorian Landcare Forum, Landcare Victoria, Creswick, Victoria, p. 28.

Landcare Victoria, Department of Sustainability and Environment, 2008c. Shaping our future: Stories. Presented at the 2008 Victorian Landcare Forum, Landcare Victoria, Creswick, Victoria, p. 42.

Landcare Victoria, Department of Sustainability and Environment, 2008d. Shaping our future: Stories. Presented at the 2008 Victorian Landcare Forum, Landcare Victoria, Creswick, Victoria, p. 34.

Lockie, S., 1998. Landcare in Australia: Cultural transformation in the management of rural environments. Culture & Agriculture 20, 21–29.

Love, C., 2012. Evolution of Landcare in Australia: In the context of Australian Government natural resource management policy and programs (Perspectives from the former National Landcare Facilitator). Australian Landcare Council Secretariat.

MacGregor, S., Pardoe, S., Dobson, A., Bell, D., 2005a. Environmental citizenship: the Goodenough primer (summary report of an interdisciplinary seminar series) (Summary Report). The Open University, Milton Keynes, UK.

MacGregor, S., Pardoe, S., Dobson, A., Bell, D., 2005b. Environmental citizenship: the Goodenough primer (summary report of an interdisciplinary seminar series) (Summary Report). The Open University, Milton Keynes, UK.

McTernan, R., Scully, M.-A., 2010. Finding common ground: A review of Indigenous engagement in Landcare in Victoria. Victorian Government Department of Sustainability and Environment, Melbourne, Victoria.

Mues, C., Chapman, L., Van Hilst, R., 1998. Promoting improved land management practices on Australian farms: A survey of landcare and land management related programs (Research report No. 98.4), Landcare. Australian Bureau of Agricultural and Resource Economics, Canberra, ACT.

Mullen, J.D., 2001. An economic perspective on land degredation issues (Economic Research Report No. 9). NSW Agriculture, Orange, NSW.

Nicholson, C., Knight, A., 2003. Auditing 10 years of Landcare in the Woady Yaloak catchment: How are we progressing along the triple bottom line? Presented at the National Landcare Conference, Darwin, NT, p. 7.

Quealy, J., 1998. Landcare: Rural Landcare. Landcare New South Wales, Sydney, NSW.

Sobels, J., Curtis, A., 2001a. Landcare networks: Establishing viable local organisations in rural Australia. Natural Resource Management 4, 22–30.

Sobels, J., Curtis, A., 2001b. Second generation Landcare support in West Gippsland (report for Landcare Victoria No. 156). Johnstone Centre, Charles Sturt University, Albury, NSW.

Sobels, J., Curtis, A., Lockie, S., 2001. The role of Landcare group networks in rural Australia: Exploring the contribution of social capital. Journal of Rural Studies 17, 265–276.

Stern, P.C., 2000a. Toward a coherent theory of environmentally significant behaviour. Journal of Social Issues 56, 407–424.

Stern, P.C., 2000b. New environmental theories: Toward a coherent theory of environmentally significant behavior. Journal of social issues 56, 407–424.

Toyne, P., Farley, R., 2000. The decade of Landcare: Looking backward - looking forward (Discussion Paper No. 30). The Australia Institute.

Vanclay, F.M., Lockie, S. (Eds.), 2000. Critical Landcare, Key papers series. Centre for Rural Social Research, Charles Sturt University, Wagga Wagga, NSW.

Walker, R.H., 2000. The National Landcare Program: What has it changed? (A compilation of evaluations of the National Landcare Program 1992-1998). Department of Agriculture, Fisheries and Forestry, Canberra, ACT.

Webb, T., Cary, J., 2005. Social capital and natural resource management: An application to Landcare. Rural Society 15, 119–131.

Woodhill, J., 1992. Landcare in NSW, taking the next step! Final report and recommendations for the development of Landcare in NSW from the 1990/91 (Final report for the National Soil Conservation Program). Department of Conservation and Land Management, Chatswood, NSW.

Youl, R. (Ed.), 2006. Landcare in Victoria: How Landcare Helped People, Government and Business Work Together in Victoria - a Collection. R. Youl, South Melbourne, Victoria.

Youl, R., Marriott, S., Nabben, T., 2006. Landcare in Australia: Founded on Local Action, 5th ed. SILC, Melbourne, Victoria.

**7.2 Social capital**

One of the key benefits of Landcare has been the precipitation and enhancement of social capital. Social capital describes the relationships in a community that enables participants to more effectively act together (Sobels et al., 2001). Beilin and Reichelt, 2010 state "Yes, there is a role for government to provide regulation, incentives and some investment; but the majority of the knowledge and effort and success is going to come from local groups…Social capital is critical".

Social capital has been an important element in the success of Landcare. The dynamic nature of Landcare has fostered social cohesion within communities, which has, in turn, further enhanced the benefits of Landcare. This social capital has been generated through the dynamics of Landcare and has enhanced the Landcare Program (Curtis, 2003). Community Landcare groups help build social capital by acting through social networks to establish trust and social bonding, and to generate land management norms and standards as well as reciprocal relationships (Beilin and Reichelt, 2010; Cary and Webb, 2000; Youl et al., 2006).

A defining feature of Landcare is that its members feel part of a community that provides mutual support, encouragement and reinforcement (Toyne and Farley, 2000) in order to work towards a common goal (Catacutan et al., 2009). This fabric has been instrumental in changing norms about good farming practices, sustainability and land conservation in rural areas (Campbell, 2009; Cary and Webb, 2001; Toyne and Farley, 2000).

The social benefits from Landcare are characterised succinctly by Brown, 1997 who states "The results of landcare programs are demonstrable: farmers now walk over each other's farms, once socially unthinkable. City councils team with rural towns, and learn from one another. Economists, bee-keepers and foresters each have a value for ghost gum (Eucalyptus papuana) flowers, a different value that each had not previously appreciated. Further to this, women farmers find that they have a voice in local agricultural meetings for the first time" (Brown, 1997).

Landcare has built or enhanced social capital that is drawn on in many ways in order to continually enhance the social fabric of rural communities. Landcare contributes to a community's social capital through building relationships, providing new and stronger governance, building resilience, enhancing the benefits of localism, increasing the recognition of women in rural communities and empowering individuals by building self identity and self recognition. Webb and Cary, 2005 even suggest that community Landcare members are distinct in terms of some socio-demographic and farm business characteristics when compared to non-members. The authors say that members generally have larger properties, more livestock and crop areas and participate in more training activities.

A recent by Deakin University, commissioned by Parks Victoria looking at the relationship between human wellbeing and contact with natural or green space environments states that while the relationship between social capital and the biophysical environment is still being explored, it appears likely that human contact with nature through natural parks could have significant capacity for building social capital (Maller et al., 2008, 2002). By increasing time spent in natural or green spaces, Landcare therefore builds social capital.

The following passages provide a synthesis of evidence of the social capital benefits resulting from Landcare in some key areas defined by the literature.

**Partnerships and networks**

Landcare has provided the impetus for groups to self organise into higher level structures or networks. These networks became even more effective at achieving outcomes, dealing with bureaucracy, increased ability to adapt to change and to discuss more complex ideas (Sobels et al., 2001; Youl et al., 2006). Typically networks exhibit a more professional management approach, have a stronger power base and are more autonomous than most groups. Networks can enhance outcome achievements including: efficiencies in coordination within and between groups, enhanced communication structures, increased resources for Landcare, increased on-ground work, adoption of a regional perspective, increase ability to tackle problems at a district scale and recruitment of skilled community leaders (Sobels and Curtis, 2001a). Curtis and Sample, 2010 reported that two-thirds of Victorian Landcare members agreed that Landcare networks helped their group in four main ways; access to information (87 per cent agreed); a forum for discussing landcare issues (78 per cent agreed); assistance so that local groups can work together (73 per cent agreed); and help to access funds (66 per cent agreed).

Landcare groups and Indigenous communities have many common interests centred on through their shared goals for conservation. The Landcare program has been able to provide opportunities for Indigenous and non-Indigenous groups to engage with one another, build relationships, and contribute to improved knowledge, understanding and participation in NRM (Landcare Victoria and Department of Sustainability and Environment, 2008; McTernan and Scully, 2010). Through participation in Landcare groups and these partnerships, Indigenous people are also able to learn new skills in environmental management, as well as make contact with decision-making NRM agencies (McTernan and Scully, 2010).

Landcare has developed new partnerships, strengthened existing friendships and partnerships, and has assisted in breaking down barriers within the community (Curtis, 2003; Curtis et al., 1999; Landcare Victoria and Department of Sustainability and Environment, 2008a; Lockie, 1998). A study by Curtis, 2003 and Curtis et al., 2008 of three Landcare groups revealed that building relationships had established:

- Increased levels of trust increasing efficiencies of transaction costs , reducing conflict in resolving difficult issues and facilitating communication and learning leading to adoption of more sustainable farming practices

- Developing new norms of behaviour including trailing new practices, better financial management, better project management including monitoring and reporting and better demonstration of outcomes

- Better reciprocal relationships between landholders, agency staff and leaders expecting support regarding access to money, materials, labour or information

The emergence of Landcare networks, involving the organisation of groups, was a development that was largely unforseen and is seen as one of the most substantial achievements of Landcare. Surveys and industry contacts suggest that up to 75 per cent of all groups in some states were involved in these networks (Sobels and Curtis, 2001a). Landcare networks have facilitated the emergence of more professional, strategic Landcare planning and action (Curtis, 2003). The development of networks has provided new skills in governance, financial management, relationship building and negotiation. These skills have been carried into other areas outside of Landcare.

The linking together of Landcare groups has been a way of increasing their capacity to compete for resources and enhance their impact on agencies and funding bodies (Curtis, 1999; Lockie, 1998). Networks are important to local organisations and have been shown to enhance the impact of groups by improving inter-group communication and attracting resources. Networks have also effectively gathered community views on key issues, provided a forum discussion of important topics, and improved the flow of information groups and the wider community (Curtis, 1999; Curtis et al., 1999; Lockie, 1998).

**A new political voice**

A positive but unexpected outcome from the formation of Landcare groups has been the creation of a new political force in the rural Australia. Many landholders have been able to harness their new Landcare organisation to tackle many issues, such as declining services in regional Australia. Some Landcare groups became powerful voices within a framework that sat quite outside the traditional farm organisations and were capable of acting independently from them (Toyne and Farley, 2000). These groups have the potential to play a critical role in changing the way that services and funding are delivered to the bush. Many Landcare groups are learning the political benefits of effective local and regional organisation, in pursuit of mutually beneficial goals (Toyne and Farley, 2000).

**Governance and self regulation**

One of the recognised strengths of Landcare is its diversity, in its members, its geography, its governance and issues. Diversity arises due to localised community-driven voices and because of the inclusiveness of Landcare, which aims to involve the whole community. Landcare has shown to effectively engage with the young and old, farmers and urban dwellers, brown and green. For these reasons, in addition to the wealth of knowledge and skills held by its volunteers, Landcare is seen as respectable and credible amongst the community and throughout government (Landcare Victoria and Department of Sustainability and Environment, 2008a). Diversity ensures synergy of the collective wisdom, drawing on the expertise and knowledge of each other. This uniting of diverse and complementary interests assists in the long-term success of the programme (Catacutan et al., 2009). Diversity also provides resilience and this is discussed further in the section on resilience in this report.

National community based efforts are effective for Landcare by providing technical support, organizing field work teams and contributing in other broad ways to support locally driven actions. These organised entities at the national level are important because without them the efforts of Landcare participants would not be easily observed by state and federal governments (Catacutan et al., 2009).

Colliver, 2011 puts forward that there is a dynamic, on-going, self-organised process of learning-by-doing at work in community-based governance finds expression in governance practice that cultivate relationships of mutual responsibility" (Colliver, 2011). In a report reviewing Landcare it is concluded that "The outcomes from this large Australian supported experiment in community NRM development has created a much better and more sophisticated community-industry-government dynamic in rural Australia that is maturing to the point where it can address the major challenges in NRM" (Griffin NRM and URS Australia Pty Ltd, 2001).

Given the structures developed and the driving forces such as the motivation of social cohesion and peer support, it is believed that it is likely that a landcare movement of

some type, even without government support is likely to persist (Coree Consulting Pty Ltd, 2003).

**Localism and empowerment**

Many members are attracted to Landcare because it embraces local community-based planning and action, groups are quite independent in determining their priorities and activities and there has been strong support for government through funding of coordinators, cost-sharing for on-ground work and the development of regional catchment management processes (Curtis, 2003). Landcare also creates networks for social support which helps to share the stress of land management issues and rural decline (Campbell, 1995a). Local level discussion and experimentation has been shown to be critical to the development and adoption of sustainable farming practices. Curtis, 2003 reports that research in Victoria confirms the importance of landholder confidence in recommended practices as a critical factor affecting adoption of sustainable farming practices (Curtis, 2003).

Community landcare groups have had the freedom to define their desired outcomes and are actively involved in generating actions for implementation. With this comes responsibility, accountability and the necessary budget that must be held accountable at the community level. It is reported that without ownership, the enthusiasm and commitment of local communities may quickly dissipate. Under a Landcare model, governments provide direct or indirect support without necessarily taking the lead. This trust in community ability at the government level brings out stronger community empowerment (Catacutan et al., 2009).

Local heroes and leaders have been uncovered through Landcare (Edmonson, 2010). This has created a positive legacy from Landcare.

**Increasing the recognition of women in rural communities**

Landcare is far more inclusive of women than any other farm-based organisation (Lockie, 1998). It has assisted in raising the profile of the role of women in agricultural family business (Hogan and Cumming, 1997). Women comprise approximately 30 per cent of all Landcare participants and have taken on roles of leadership that has been a positive experience for most women (Curtis, 2003).

**Personal growth**

Landcare has also had many benefits at the personal level. The contribution of these people to groups and networks has contributed to the success of Landcare. Many people in Landcare have learnt a lot about their own properties, about the land in their district and about issues they may have rarely considered in the past (Campbell, 1992). Campbell also states that group leaders in particular have gained much from seeing other people get involved, from influencing others through their interaction in the group and occasionally from group projects (Campbell, 1992). Similarly Gooch, 2004 states that volunteers develop action confidence over time through the development of self-confidence acquired through learning and networking, and the ability and willingness to remain active within the group (Gooch, 2004).

Another important aspect of personal growth is the concept of identity within volunteer groups. Identify helps to build social communities, ecological identity, and a sense of place, all of which are elements of resilient, sustainable communities (see section on resilience benefits in this report).

Landcare group members have remarked on Landcare's ability to promote cooperative discussions and activities surrounding land management practices and problems within the community, thus restoring the "sense of community" that seems to be getting lost in many rural communities (Lockie, 1998). Gooch found that a sense of place was a strong motivator for many of volunteers, reflecting the need for people to feel connected to their communities. Involvement in Landcare volunteering could provide a counter to contemporary society where many people are increasingly disconnected from places and from nature (Gooch, 2004).

#### Filling the void

Landcare has also built social capital that has filled a void that has been left through the retraction of social networks due to rural decline and government services such as agricultural extension services (Webb and Cary, 2005). The social capital built by Landcare is a resource that has will continue to be drawn on to contribute to achieving natural resource management outcomes but also other social objectives (Curtis, 2003).

#### Increasing awareness, skills and knowledge

Although this area is more fully investigated in the section on Education, Awareness and Practice Change in this report, it is important to recognise that these attributes are also a part of social capital. It is widely recognised by numerous authors that Landcare has contributed to social capital through increasing awareness, developing and extending skills and knowledge and developing networks to promote the acceptance of sustainable farming practices (Campbell, 1995b; Cary and Webb, 2001; Curtis and Cooke, 2006; Curtis and De Lacy, 1996; Curtis, 1995; Curtis et al., 1993; Department of Agriculture, 2003; Edmonson, 2010; Griffin NRM and URS Australia Pty Ltd, 2001; Lockie, 1998; Quealy, 1998; Sobels et al., 2001; Toyne and Farley, 2000). While Landcare helps to build social capital amongst communities, the real value is the long term influence on behaviour that helps to reinforce more environmental behaviour (Cary and Webb, 2001, 2000).

#### 7.2.1 Citations

Altman, J.C., Whitehead, P.J., 2003. Caring for country and sustainable Indigenous development: opportunities, constraints and innovation (CAEPR Working Paper No. 20). Centre for Aboriginal Economic Policy Research.

Beilin, R., Reichelt, N., 2010. Community Landcare: a key player in building social-ecological resilience networks? The State of Victoria Department of Sustainability and Environment, Melbourne.

Brown, V., 1997. Landcare in Australia: talking local sustainability in policy, practice and place (RDFN Paper No. 20e). Rural Development Forestry Network, London, UK.

Buikstra, E., Ross, H., King, C.A., Baker, P.G., Hegney, D., McLachlan, K., Rogers-Clark, C., 2010. The components of resilience - perceptions of an Australian rural community. Journal of Community Psychology 38, 975–991.

Campbell, A., 1992. Taking the long view in tough times: Landcare in Australia (Third Annual Report), National Landcare Facilitator. National Soil Conservation Program.

Campbell, A., 1995a. Land literacy in Australia. Journal of Soil and Water Conservation 126–131.

Campbell, C.A., 1995b. Landcare: Participative Australian approaches to inquiry and learning for sustainability. Journal of Soil and Water Conservation 50, 125–131.

Campbell, C.A., 2009. It's time to renew Landcare. Agricultural Science 30–33.

Cary, J., Webb, T., 2000. Community landcare, the National Landcare Program and the landcare movement: the social dimensions of landcare. Bureau of Rural Science, Canberra, ACT.

Cary, J., Webb, T., 2001. Landcare in Australia: Community participation and land management. Journal of Soil and Water Conservation 56, 274–278.

Catacutan, D., Neely, C., Johnson, M., Youl, R. (Eds.), 2009. Landcare: Local Action - Global Progress. World Agroforestry Centre, Nairobi, Kenya.

Colliver, R., 2011. Community-based governance in social-ecological systems: An inquiry into the marginalisation of Landcare in Victoria, Australia (PhD of Philosophy Thesis).

Compton, E., Hayne, W., Beeton, R.J.S., 2007. Six years in a cement mixer: A landcare journey through changing times. Presented at the International Landcare Conference, Melbourne, Victoria.

Coree Consulting Pty Ltd, 2003. Landcare Literature Review. Coree Consulting Pty Ltd.

Curtis, A., 1995a. Landcare in Australia: A critical review (report No. 33). Charles Sturt University, Albury, NSW.

Curtis, A., 1995b. Landcare in Victoria: The state of play (Report for Landcare Victoria No. 24). Johnstone Centre, Charles Sturt University, Albury, NSW.

Curtis, A., 1999. Landcare in Victoria: Beyond onground work (Report No. 125). Johnstone Centre, Charles Sturt University, Albury, NSW.

Curtis, A., 2003. Reflecting on the Landcare experience: A report based on information held within ABARE and BRS. Bureau of Rural Sciences.

Curtis, A., Britton, A., Sobels, J., 1999. Landcare networks in Australia: State-sponsored participation through local organizations. Journal of Environmental Planning and Management 42, 5–21.

Curtis, A., Cooke, P., 2006. Landcare groups in Victoria: After twenty years (Report to the Australian Government Deparment of Agriculture, Fisheries and Forestry, National Landcare Program, Monitorin and Evaluation Project). Instutute for Land, Water and Society, and the National Landcare Program, Albury, NSW.

Curtis, A., De Lacy, T., 1995. Evaluating landcare groups in Australia: How they facilitate partnerships between agencies, community groups, and researchers. Journal of Soil and Water Conservation January-February, 15–20.

Curtis, A., De Lacy, T., 1996a. Landcare in Australia: Does it make a difference? Journal of Environmental Management 46, 119–137.

Curtis, A., De Lacy, T., 1996b. Landcare in Australia: Beyond the expert farmer. Agriculture and Human Values 13, 20–32.

Curtis, A., De Lacy, T., 1996c. Landcare in Australia: Does it make a difference? Johnstone Centre, Charles Sturt University, Albury, NSW.

Curtis, A., De Lacy, T., 1998. Landcare, stewardship and sustainable agriculture in Australia. Environmental Values 7, 59–78.

Curtis, A., De Lacy, T., Klomp, N., 1993. Asessing the effectiveness of landcare groups: The Victorian experience. Regional Journal of Social Issues 27, 78–91.

Curtis, A., Lockwood, M., 2000. Landcare and catchment management in Australia: Lessons for state-sponsored community participation. Society & Natural Resources 13, 61–73.

Curtis, A., Lucas, D., Nurse, M., Skeen, M., 2008. Achieving NRM outcomes through voluntary action: Lessons from Landcare (Discussion Paper). Charles Sturt University & Victorian Department of Sustainability and Environment, Melbourne, Victoria.

Curtis, A., Sample, R., 2010. CBNRM in Victoria: Contributing to dialogue, learning and action (ILWS Report No. 55). Institute for Land, Water and Society, Charles Sturt University, Albury, NSW.

Curtis, A., Van Nouhuys, M., Robinson, W., MacKay, J., 2000. Exploring Landcare effectiveness using organisational theory. Australian Geographer 31, 349–366.

Department of Agriculture, 2003. Review of the National Landcare Program (Report). Department of Agriculture, Canberra, ACT.

Dietz, T., Stern, P.C. (Eds.), 2002. New Tools for Environmental Protection: Education, Information, and Voluntary Measures. The National Academies Press, Washington, D.C.

Edmonson, R., 2010. Reviewing Landcare.

Garnett, S.T., Sithole, B., Whitehead, P.J., Burgess, C.P., Johnston, F.H., Lea, T., 2009. Healthy country, healthy people: Policy implications of links between Indigenous human health and environmental condition in tropical australia. Australian Journal of Public Administration 68, 53–66.

Gooch, M.J., 2004. Voices of the volunteers: an exploration of the influences that volunteer experiences have on the resilience and sustainability of catchment groups in coastal queensland (PhD Thesis).

Griffin NRM, URS Australia Pty Ltd, 2001. An evaluation of investment in Landcare support projects (Final report for Agriculture, Fisheries and Forestry Australia). URS Australia Pty Ltd and Griffin NRM.

Gunningham, N., 2002. Reconfiguring Environmental Regulation: The Future Public Policy Agenda. N. Gunningham & D. Sinclair ds., Leaders and Laggards: Ne t Generation nvironmental Regulation pp.

Gunningham, N., Kagan, R.A., Thornton, D., 2004. Social license and environmental protection: why businesses go beyond compliance. Law & Social Inquiry 29, 307–341.

Gunningham, N., Sinclair, D., 1998. Designing smart regulation, in: Economic Aspects of Environmental Compliance Assurance. OECD Global Forum on Sustainable Development.

Hamilton, C., 1995. The economic language of Landcare (Background Paper No. 3). The Australia Institute, Canberra, ACT.

Hawthorne, M., Alabaster, T., 1999. Citizen 2000: development of a model of environmental citizenship. Global Environmental Change 9, 25–43.

Hegney, D., Ross, H., Baker, P., Buikstra, E., King, C., McLachlan, K., Rogers-Clark, C., Stallard, L., Watson-Luke, A., 2008. Identification of personal and community resilience that enhance psychological wellness: A Stanthorpe study. Australian Research Council, Canberra, ACT.

Hogan, L., Cumming, B., 1997. More than a question of numbers: Working with women and people from a non-English speaking background toward total catchment participation (Report of the Landcare Participation Project). Department of Primary Industries and Energy, and the Department of Natural Resources and Environment, Canberra, ACT.

Horvath, D. (Ed.), 2001. NSW Decade of Landcare Evaluation. NSW Department of Land and Water Conservation, Sydney, NSW.

Landcare Victoria, Department of Sustainability and Environment, 2008a. Shaping our future: Proceedings. Presented at the 2008 Victorian Landcare Forum, Landcare Victoria, Creswick, Victoria, p. 21.

Landcare Victoria, Department of Sustainability and Environment, 2008b. Shaping our future: Proceedings. Presented at the 2008 Victorian Landcare Forum, Landcare Victoria, Creswick, Victoria, p. 28.

Landcare Victoria, Department of Sustainability and Environment, 2008c. Shaping our future: Stories. Presented at the 2008 Victorian Landcare Forum, Landcare Victoria, Creswick, Victoria, p. 42.

Landcare Victoria, Department of Sustainability and Environment, 2008d. Shaping our future: Stories. Presented at the 2008 Victorian Landcare Forum, Landcare Victoria, Creswick, Victoria, p. 34.

Lockie, S., 1998. Landcare in Australia: Cultural transformation in the management of rural environments. Culture & Agriculture 20, 21–29.

Love, C., 2012. Evolution of Landcare in Australia: In the context of Australian Government natural resource management policy and programs (Perspectives from the former National Landcare Facilitator). Australian Landcare Council Secretariat.

MacGregor, S., Pardoe, S., Dobson, A., Bell, D., 2005a. Environmental citizenship: the Goodenough primer (summary report of an interdisciplinary seminar series) (Summary Report). The Open University, Milton Keynes, UK.

MacGregor, S., Pardoe, S., Dobson, A., Bell, D., 2005b. Environmental citizenship: the Goodenough primer (summary report of an interdisciplinary seminar series) (Summary Report). The Open University, Milton Keynes, UK.

McTernan, R., Scully, M.-A., 2010. Finding common ground: A review of Indigenous engagement in Landcare in Victoria. Victorian Government Department of Sustainability and Environment, Melbourne, Victoria.

Mues, C., Chapman, L., Van Hilst, R., 1998. Promoting improved land management practices on Australian farms: A survey of landcare and land management related programs (Research report No. 98.4), Landcare. Australian Bureau of Agricultural and Resource Economics, Canberra, ACT.

Mullen, J.D., 2001. An economic perspective on land degredation issues (Economic Research Report No. 9). NSW Agriculture, Orange, NSW.

Nicholson, C., Knight, A., 2003. Auditing 10 years of Landcare in the Woady Yaloak catchment: How are we progressing along the triple bottom line? Presented at the National Landcare Conference, Darwin, NT, p. 7.

Quealy, J., 1998. Landcare: Rural Landcare. Landcare New South Wales, Sydney, NSW.

Sobels, J., Curtis, A., 2001a. Landcare networks: Establishing viable local organisations in rural Australia. Natural Resource Management 4, 22–30.

Sobels, J., Curtis, A., 2001b. Second generation Landcare support in West Gippsland (report for Landcare Victoria No. 156). Johnstone Centre, Charles Sturt University, Albury, NSW.

Sobels, J., Curtis, A., Lockie, S., 2001. The role of Landcare group networks in rural Australia: Exploring the contribution of social capital. Journal of Rural Studies 17, 265–276.

Stern, P.C., 2000a. Toward a coherent theory of environmentally significant behaviour. Journal of Social Issues 56, 407–424.

Stern, P.C., 2000b. New environmental theories: Toward a coherent theory of environmentally significant behavior. Journal of social issues 56, 407–424.

Toyne, P., Farley, R., 2000. The decade of Landcare: Looking backward - looking forward (Discussion Paper No. 30). The Australia Institute.

Vanclay, F.M., Lockie, S. (Eds.), 2000. Critical Landcare, Key papers series. Centre for Rural Social Research, Charles Sturt University, Wagga Wagga, NSW.

Walker, R.H., 2000. The National Landcare Program: What has it changed? (A compilation of evaluations of the National Landcare Program 1992-1998). Department of Agriculture, Fisheries and Forestry, Canberra, ACT.

Webb, T., Cary, J., 2005. Social capital and natural resource management: An application to Landcare. Rural Society 15, 119–131.

Woodhill, J., 1992. Landcare in NSW, taking the next step! Final report and recommendations for the development of Landcare in NSW from the 1990/91 (Final report for the National Soil Conservation Program). Department of Conservation and Land Management, Chatswood, NSW.

Youl, R. (Ed.), 2006. Landcare in Victoria: How Landcare Helped People, Government and Business Work Together in Victoria - a Collection. R. Youl, South Melbourne, Victoria.

Youl, R., Marriott, S., Nabben, T., 2006. Landcare in Australia: Founded on Local Action, 5th ed. SILC, Melbourne, Victoria.

### 7.3 Economic

The Landcare program has made a long-term, positive impact on the environmental condition, as well as the economic profitability of farming (Curtis, 2003; Sobels et al., 2001).

Landcare Victoria and Department of Sustainability and Environment, 2008a, 2008b, 2008c, 2008d found that every dollar that is invested in Landcare groups leverages 2-5 times that amount through contributions towards labour, equipment, voluntary expertise, and often additional donations from landholders and businesses.

Landcare has provided economic benefits to communities, as identified in the literature, primarily through increased access to financial resources, and training to improve farming and management techniques.

#### Access to resources

Landcare networks have become important local organisations through their ability to establish partnerships that help to reduce financial risk (Curtis and Lockwood, 2000).

The increased communication and partnerships that emerge from participation in Landcare networks, results in a greater awareness and appreciation for the different values placed on environmental assets by economists, the community and landholders (Brown, 1997). This mutual understanding helps to actively include private businesses in farming, creating more economically sustainable land management systems and improved market share, profits and economic resilience (Catacutan et al., 2009). Investing in Landcare groups is also economically beneficial to corporations through the subsequent association with conservation ethics, which often helps to improve relations with the community (Catacutan et al., 2009).

Additionally, Landcare networks have enhanced the ability of community groups to access funds from Government and other organisations (Curtis et al., 1999; Sobels and Curtis, 2001b; Compton et al., 2007). The Department of Agriculture Review of the National Landcare Program, 2003 discovered that Landcare groups were able to draw down additional funding from non-government parties at a rate of at least $2.60 for every $1.00 spent by the government on Landcare projects.

Landcare groups also help reconnect Indigenous people with country (Love, 2012). Altman and Whitehead (2003) and Garnett et al. (2009) found that when Indigenous people are connected with country, they are better able to participate in the market sector by utilising natural resources, and generate income for themselves. Support generated by the Landcare program is therefore able to facilitate sustainable economic development in Indigenous communities while improving disadvantages. This has inherent socio-cultural benefits for Indigenous people and, in addition, promotes spiritual well-being and physical health (Altman and Whitehead, 2003).

#### Training and management techniques

Landcare groups make it possible for farmer to take greater financial risks with how they manage their farms, but this allows them to receive improved outcomes in the long run, financially and environmentally (Campbell, 1992). Mues et al. (1998) found that greater involvement in training associated with Landcare participation resulted in larger farm debts initially, and greater physical changes in farm characteristics and higher farm cash

incomes over a longer period of time. Without conservation activities and implementation of more up to date sustainable farming techniques, significant costs can be associated with land degradation in future generations through the loss of production, reduced biodiversity and loss of environmental resilience (Figure 1.) (Hamilton, 1995; Mullen, 2001).

**Figure 1. Net income per hectare with and without conservation (Hamilton, 1995)**

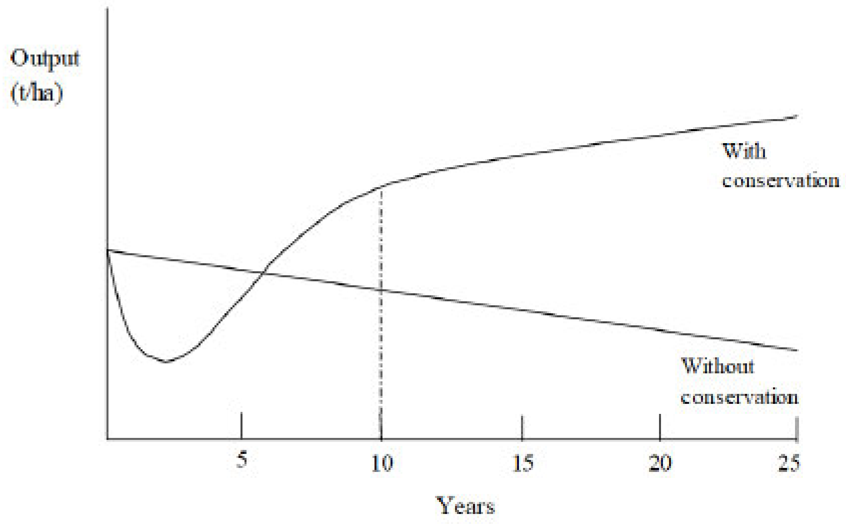


A case study conducted by Nicholson and Knight, 2003 in the Woady Yoloak Catchment found an increase in gross income from $275/ha in 1990 to approximately $335/ha in 2001. The participants attributed this increase in part to an increase in commodity prices and property size, but also to improved productivity of the enterprise (Nicholson and Knight, 2003). The increase in productivity was accredited to an increase in average fertilizer used, increased expenditure on pasture fencing, an increase in the amount of perennial pastures on the average property and the use of better management practices and technologies, which could all be supported through participation with Landcare groups.

#### 7.3.1 Citations

Altman, J.C., Whitehead, P.J., 2003. Caring for country and sustainable Indigenous development: opportunities, constraints and innovation (CAEPR Working Paper No. 20). Centre for Aboriginal Economic Policy Research.

Beilin, R., Reichelt, N., 2010. Community Landcare: a key player in building social-ecological resilience networks? The State of Victoria Department of Sustainability and Environment, Melbourne.

Brown, V., 1997. Landcare in Australia: talking local sustainability in policy, practice and place (RDFN Paper No. 20e). Rural Development Forestry Network, London, UK.

Buikstra, E., Ross, H., King, C.A., Baker, P.G., Hegney, D., McLachlan, K., Rogers-Clark, C., 2010. The components of resilience - perceptions of an Australian rural community. Journal of Community Psychology 38, 975–991.

Campbell, A., 1992. Taking the long view in tough times: Landcare in Australia (Third Annual Report), National Landcare Facilitator. National Soil Conservation Program.

Campbell, A., 1995a. Land literacy in Australia. Journal of Soil and Water Conservation 126–131.

Campbell, C.A., 1995b. Landcare: Participative Australian approaches to inquiry and learning for sustainability. Journal of Soil and Water Conservation 50, 125–131.

Campbell, C.A., 2009. It's time to renew Landcare. Agricultural Science 30–33.

Cary, J., Webb, T., 2000. Community landcare, the National Landcare Program and the landcare movement: the social dimensions of landcare. Bureau of Rural Science, Canberra, ACT.

Cary, J., Webb, T., 2001. Landcare in Australia: Community participation and land management. Journal of Soil and Water Conservation 56, 274–278.

Catacutan, D., Neely, C., Johnson, M., Youl, R. (Eds.), 2009. Landcare: Local Action - Global Progress. World Agroforestry Centre, Nairobi, Kenya.

Colliver, R., 2011. Community-based governance in social-ecological systems: An inquiry into the marginalisation of Landcare in Victoria, Australia (PhD of Philosophy Thesis).

Compton, E., Hayne, W., Beeton, R.J.S., 2007. Six years in a cement mixer: A landcare journey through changing times. Presented at the International Landcare Conference, Melbourne, Victoria.

Coree Consulting Pty Ltd, 2003. Landcare Literature Review. Coree Consulting Pty Ltd.

Curtis, A., 1995a. Landcare in Australia: A critical review (report No. 33). Charles Sturt University, Albury, NSW.

Curtis, A., 1995b. Landcare in Victoria: The state of play (Report for Landcare Victoria No. 24). Johnstone Centre, Charles Sturt University, Albury, NSW.

Curtis, A., 1999. Landcare in Victoria: Beyond onground work (Report No. 125). Johnstone Centre, Charles Sturt University, Albury, NSW.

Curtis, A., 2003. Reflecting on the Landcare experience: A report based on information held within ABARE and BRS. Bureau of Rural Sciences.

Curtis, A., Britton, A., Sobels, J., 1999. Landcare networks in Australia: State-sponsored participation through local organizations. Journal of Environmental Planning and Management 42, 5–21.

Curtis, A., Cooke, P., 2006. Landcare groups in Victoria: After twenty years (Report to the Australian Government Department of Agriculture, National Landcare Program, Monitoring and Evaluation Project). Institute for Land, Water and Society, and the National Landcare Program, Albury, NSW.

Curtis, A., De Lacy, T., 1995. Evaluating landcare groups in Australia: How they facilitate partnerships between agencies, community groups, and researchers. Journal of Soil and Water Conservation January-February, 15–20.

Curtis, A., De Lacy, T., 1996a. Landcare in Australia: Does it make a difference? Journal of Environmental Management 46, 119–137.

Curtis, A., De Lacy, T., 1996b. Landcare in Australia: Beyond the expert farmer. Agriculture and Human Values 13, 20–32.

Curtis, A., De Lacy, T., 1996c. Landcare in Australia: Does it make a difference? Johnstone Centre, Charles Sturt University, Albury, NSW.

Curtis, A., De Lacy, T., 1998. Landcare, stewardship and sustainable agriculture in Australia. Environmental Values 7, 59–78.

Curtis, A., De Lacy, T., Klomp, N., 1993. Asessing the effectiveness of landcare groups: The Victorian experience. Regional Journal of Social Issues 27, 78–91.

Curtis, A., Lockwood, M., 2000. Landcare and catchment management in Australia: Lessons for state-sponsored community participation. Society & Natural Resources 13, 61–73.

Curtis, A., Lucas, D., Nurse, M., Skeen, M., 2008. Achieving NRM outcomes through voluntary action: Lessons from Landcare (Discussion Paper). Charles Sturt University & Victorian Department of Sustainability and Environment, Melbourne, Victoria.

Curtis, A., Sample, R., 2010. CBNRM in Victoria: Contributing to dialogue, learning and action (ILWS Report No. 55). Institute for Land, Water and Society, Charles Sturt University, Albury, NSW.

Curtis, A., Van Nouhuys, M., Robinson, W., MacKay, J., 2000. Exploring Landcare effectiveness using organisational theory. Australian Geographer 31, 349–366.

Department of Agriculture, 2003. Review of the National Landcare Program (Report). Department of Agriculture, Fisheries and Forestry, Canberra, ACT.

Dietz, T., Stern, P.C. (Eds.), 2002. New Tools for Environmental Protection: Education, Information, and Voluntary Measures. The National Academies Press, Washington, D.C.

Edmonson, R., 2010. Reviewing Landcare.

Garnett, S.T., Sithole, B., Whitehead, P.J., Burgess, C.P., Johnston, F.H., Lea, T., 2009. Healthy country, healthy people: Policy implications of links between Indigenous human health and environmental condition in tropical australia. Australian Journal of Public Administration 68, 53–66.

Gooch, M.J., 2004. Voices of the volunteers: an exploration of the influences that volunteer experiences have on the resilience and sustainability of catchment groups in coastal queensland (PhD Thesis).

Griffin NRM, URS Australia Pty Ltd, 2001. An evaluation of investment in Landcare support projects (Final report for Agriculture, Fisheries and Forestry Australia). URS Australia Pty Ltd and Griffin NRM.

Gunningham, N., 2002. Reconfiguring Environmental Regulation: The Future Public Policy Agenda. N. Gunningham & D. Sinclair ds., Leaders and Laggards: Ne t Generation nvironmental Regulation pp.

Gunningham, N., Kagan, R.A., Thornton, D., 2004. Social license and environmental protection: why businesses go beyond compliance. Law & Social Inquiry 29, 307–341.

Gunningham, N., Sinclair, D., 1998. Designing smart regulation, in: Economic Aspects of Environmental Compliance Assurance. OECD Global Forum on Sustainable Development.

Hamilton, C., 1995. The economic language of Landcare (Background Paper No. 3). The Australia Institute, Canberra, ACT.

Hawthorne, M., Alabaster, T., 1999. Citizen 2000: development of a model of environmental citizenship. Global Environmental Change 9, 25–43.

Hegney, D., Ross, H., Baker, P., Buikstra, E., King, C., McLachlan, K., Rogers-Clark, C., Stallard, L., Watson-Luke, A., 2008. Identification of personal and community resilience that enhance psychological wellness: A Stanthorpe study. Australian Research Council, Canberra, ACT.

Hogan, L., Cumming, B., 1997. More than a question of numbers: Working with women and people from a non-English speaking background toward total catchment participation (Report of the Landcare Participation Project). Department of Primary Industries and Energy, and the Department of Natural Resources and Environment, Canberra, ACT.

Horvath, D. (Ed.), 2001. NSW Decade of Landcare Evaluation. NSW Department of Land and Water Conservation, Sydney, NSW.

Landcare Victoria, Department of Sustainability and Environment, 2008a. Shaping our future: Proceedings. Presented at the 2008 Victorian Landcare Forum, Landcare Victoria, Creswick, Victoria, p. 21.

Landcare Victoria, Department of Sustainability and Environment, 2008b. Shaping our future: Proceedings. Presented at the 2008 Victorian Landcare Forum, Landcare Victoria, Creswick, Victoria, p. 28.

Landcare Victoria, Department of Sustainability and Environment, 2008c. Shaping our future: Stories. Presented at the 2008 Victorian Landcare Forum, Landcare Victoria, Creswick, Victoria, p. 42.

Landcare Victoria, Department of Sustainability and Environment, 2008d. Shaping our future: Stories. Presented at the 2008 Victorian Landcare Forum, Landcare Victoria, Creswick, Victoria, p. 34.

Lockie, S., 1998. Landcare in Australia: Cultural transformation in the management of rural environments. Culture & Agriculture 20, 21–29.

Love, C., 2012. Evolution of Landcare in Australia: In the context of Australian Government natural resource management policy and programs (Perspectives from the former National Landcare Facilitator). Australian Landcare Council Secretariat.

MacGregor, S., Pardoe, S., Dobson, A., Bell, D., 2005a. Environmental citizenship: the Goodenough primer (summary report of an interdisciplinary seminar series) (Summary Report). The Open University, Milton Keynes, UK.

MacGregor, S., Pardoe, S., Dobson, A., Bell, D., 2005b. Environmental citizenship: the Goodenough primer (summary report of an interdisciplinary seminar series) (Summary Report). The Open University, Milton Keynes, UK.

McTernan, R., Scully, M.-A., 2010. Finding common ground: A review of Indigenous engagement in Landcare in Victoria. Victorian Government Department of Sustainability and Environment, Melbourne, Victoria.

Mues, C., Chapman, L., Van Hilst, R., 1998. Promoting improved land management practices on Australian farms: A survey of landcare and land management related programs (Research report No. 98.4), Landcare. Australian Bureau of Agricultural and Resource Economics, Canberra, ACT.

Mullen, J.D., 2001. An economic perspective on land degredation issues (Economic Research Report No. 9). NSW Agriculture, Orange, NSW.

Nicholson, C., Knight, A., 2003. Auditing 10 years of Landcare in the Woady Yaloak catchment: How are we progressing along the triple bottom line? Presented at the National Landcare Conference, Darwin, NT, p. 7.

Quealy, J., 1998. Landcare: Rural Landcare. Landcare New South Wales, Sydney, NSW.

Sobels, J., Curtis, A., 2001a. Landcare networks: Establishing viable local organisations in rural Australia. Natural Resource Management 4, 22–30.

Sobels, J., Curtis, A., 2001b. Second generation Landcare support in West Gippsland (report for Landcare Victoria No. 156). Johnstone Centre, Charles Sturt University, Albury, NSW.

Sobels, J., Curtis, A., Lockie, S., 2001. The role of Landcare group networks in rural Australia: Exploring the contribution of social capital. Journal of Rural Studies 17, 265–276.

Stern, P.C., 2000a. Toward a coherent theory of environmentally significant behaviour. Journal of Social Issues 56, 407–424.

Stern, P.C., 2000b. New environmental theories: Toward a coherent theory of environmentally significant behavior. Journal of social issues 56, 407–424.

Toyne, P., Farley, R., 2000. The decade of Landcare: Looking backward - looking forward (Discussion Paper No. 30). The Australia Institute.

Vanclay, F.M., Lockie, S. (Eds.), 2000. Critical Landcare, Key papers series. Centre for Rural Social Research, Charles Sturt University, Wagga Wagga, NSW.

Walker, R.H., 2000. The National Landcare Program: What has it changed? (A compilation of evaluations of the National Landcare Program 1992-1998). Department of Agriculture, Fisheries and Forestry, Canberra, ACT.

Webb, T., Cary, J., 2005. Social capital and natural resource management: An application to Landcare. Rural Society 15, 119–131.

Woodhill, J., 1992. Landcare in NSW, taking the next step! Final report and recommendations for the development of Landcare in NSW from the 1990/91 (Final

report for the National Soil Conservation Program). Department of Conservation and Land Management, Chatswood, NSW.

Youl, R. (Ed.), 2006. Landcare in Victoria: How Landcare Helped People, Government and Business Work Together in Victoria - a Collection. R. Youl, South Melbourne, Victoria.

Youl, R., Marriott, S., Nabben, T., 2006. Landcare in Australia: Founded on Local Action, 5th ed. SILC, Melbourne, Victoria.

**7.4 Health and wellbeing**

There are several pathways in which contact with the natural environment, as one would experience when involved with landcare activities, can improve the health and wellbeing of individuals and communities as shown below.

**Health and wellbeing outcomes from involvement in landcare activities**

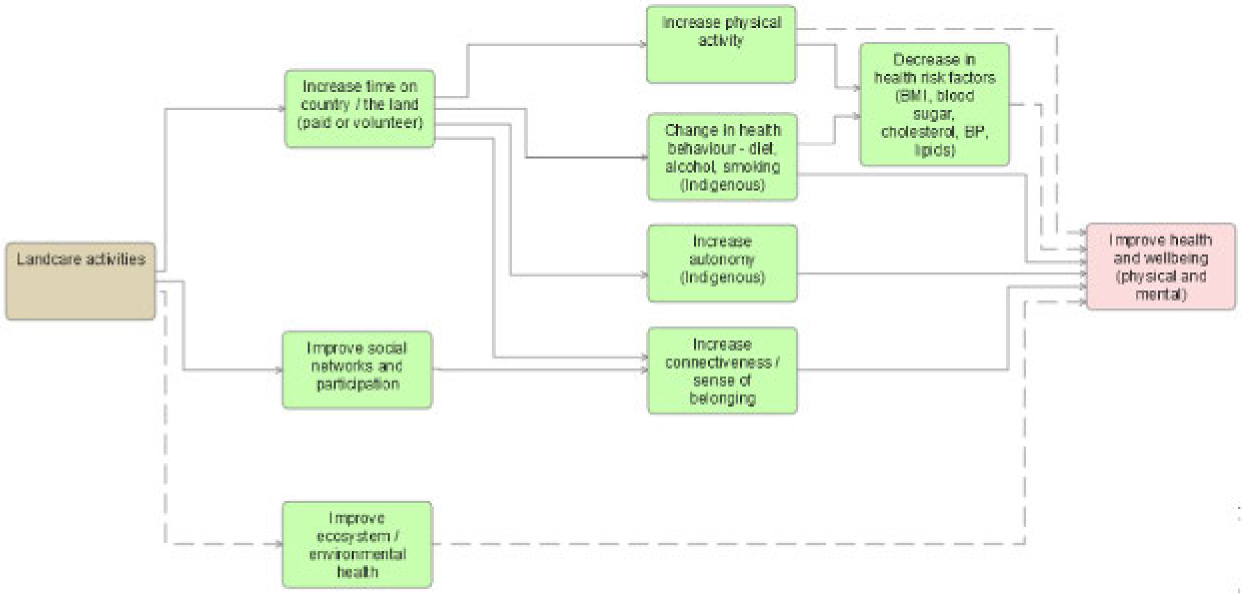


Involvement with natural resource management or landcare activities can have positive human health and wellbeing impacts through the improvement of environmental quality or ecosystem services such as cleaner water, cleaner air quality, improved aesthetics of the environment, better quality food production and enhancement of environmental services such as pollination and nutrient cycling. It could also be argued that landcare activities that reduce carbon outputs or increase carbon capture or sequestration such as vegetation enhancement activities have potential global human health benefit.

Other pathways in which improved human health and wellbeing benefits can be derived from landcare activities include improved social networks and participation leading to increased connectivity and sense of belonging and through increased time on country/land leading to a number of human physiological and mental health benefits. Within this pathway there are a range of specific benefits that have been studied for indigenous peoples. This rest of this section provides a brief overview of some of the evidence for the existence of these cause-effect pathways. Due to the scope of this review, evidence will not be provided for the relationships shown as dashed lines in the above model.

**Health benefits of contact with a natural environment**

Developed in 1980's, the hypothesis of "biophillia" describes the concept of values of nature whose expression is linked to aspects of physical, emotional, and intellectual growth and development. The hypothesis is based on the idea that people possess "an inherent inclination to affiliate with natural process and diversity, and this affinity

continues today to be instrumental in human physical and mental development" (Kellert and Derr, 1998).

There have been several seminal reviews of evidence relating to the human health benefits of contact with natural environments or green spaces undertaken in the last ten years (Maller et al., 2008, 2002; Townsend and Weerasuriya, 2010). A review was undertaken by Deakin University in 2002 (and updated in 2008) for Parks Victoria to underpin a Healthy Parks, Healthy People community message being promoted. The 2002 review synthesized over 200 items of relevant evidence with the 2008 review summarizing some important conclusions:

- Human contact with green nature, such as parks, can reduce crime, foster psychological wellbeing, enhance productivity, reduce stress, boost immunity and promote healing

- There are even health benefits from viewing nature in terms of recovering from stress, improving concentration and productivity, and improving psychological state. Studies suggest that this is particularly relevant to people in confined circumstances such as prisons and hospitals

- Plants and nearby vegetation can have profound effects on individuals, small groups, or even entire neighbourhoods. Some health benefits of interacting with plants include facilitation of healing in the elderly and mentally disadvantaged, improving mental capacity and productivity of office workers, improving job and life satisfaction of residents, attracting consumers and tourists to shopping districts, and aiding community cohesion and identity.

The Deakin University reviews conclude that the initial evidence for the positive effects of nature on blood pressure, cholesterol, outlook on life and stress reduction provides justification for its incorporation into strategies for the Australian National Health Priority Areas of mental health and cardiovascular disease (Maller et al., 2008).

In an effort to maintain global momentum for better understanding the links between nature and human health a website has been recently established. The Healthy Parks Healthy People (HPHP) Central website evolved out of the International Healthy Parks Healthy People Congress 2010 (http://www.hphpcentral.com/about).

Also recently, beyondblue, the national depression initiative, commissioned Deakin University to undertake a review of literature pertaining to the benefits of contact with nature for mental health and well-being. The beyondblue review distinguishes health benefits being derived from three different levels of contact with nature: viewing nature, being in the presence of nearby nature and active participation in nature including farming.

The beyondblue review draws on evidence to confirm that physical activity in natural settings greatly improves self-esteem and positive emotions and behaviour and that natural settings promote social exchanges and interactions resulting in positive emotional states and behaviours (Townsend and Weerasuriya, 2010). The review looks at the evidence relating to several areas including the relationships between parks and green spaces as setting for therapeutic nature contact, gardens as having therapeutic benefits, ecotherapy, adventure and wilderness therapy, the health benefits of green exercise, care farming and school ground greening the health benefits of contact with animals. In one study in Toronto, Canada participants reported that the presence of neighbourhood

green space provided self-reported physical and mental health benefits to those living close to nature or green spaces (De Vries et al., 2003).

**Health benefits of social networks**

Landcare is based on the interaction of the social aspects of a community and the natural resources that are inherent in the local areas with the understanding that community action is required to meet the significant environmental challenges. Through this thinking both the physical environment and the sense of community of participants are improved (Pretty et al., 2007).

Social epidemiologists have demonstrated how community connections, networks, belonging, social cohesion, and social capital play a pivotal role in the health, well-being and mental health outcomes of populations (Pretty et al., 2007). Increased social interaction and participation by an individual within a community enforces a sense of belonging and social connectedness and this has been well linked to positive physical and psychological wellbeing (Cannon, 2008). It is also believed that a sense of community provides a buffer against physical and psychological symptoms of illness, and facilitates adjustment (Pretty et al., 2007).

Cattell (2001) states that individuals with many informal networks are less likely to suffer ill health, as these networks provide support, clarify personal identity, enhance self-esteem and enable citizens to feel in control of their lives.

Social capital is characteristic of "healthy, thriving communities and is strengthened through voluntary activities and organisations" (Gooch, 2003). In a study by Koss and Kingsley (2010) of volunteers participating in marine program Sea Search, volunteers responded that their involvement in voluntary activities made them feel good emotionally and mentally, with active learning, such as remembering names of marine biota, stimulating brain activity and memory and that volunteer monitoring efforts generated personal satisfaction through their contributions, feelings of enjoyment, and socialising with others.

Baum et al (1999) conducted a study in South Australia, that concluded that volunteers were more likely to have more informal social contacts, and to be involved in a range of social activities, than individuals who did not get involved in volunteering. The social fabric of a place Baum concluded, can be reinforced through the development of social ties created through voluntary work. This observation is supported by Koss and Kingsley who state that the notion of volunteer connection to the natural environment and positive mental and emotional health are important for any citizen science monitoring program (Koss and Kingsley, 2010).

It has been suggested that in order to increase senior citizens level of health and wellbeing, and reduce the social isolation often experienced that local health centres and general practitioners should encourage senior citizens to become involved in conservation groups to (Koss and Kingsley, 2010).

The notion of sense of place is also important in creating social cohesion in involvement with landcare activities. Sense of place is not just experienced by people becoming attached to their biophysical surroundings, but can also be seen as extending to emotional attachments to social communities, built through familiarity and spending time in one place. It is believed that spending time in one place and maintaining social contacts can help to build social capital comprising trust, reciprocity, norms, values and networks (Putnam, 1993).

**Indigenous health benefits of connection with country**

There is a strong belief reflected in Indigenous cultures that if an individual does not maintain spiritual, physical, social and mental health they cannot be truly connected with the natural world (Townsend and Weerasuriya, 2010). Further to this is Burgess states that many Aboriginal people derive their self-identity from the land (Burgess et al., 2005) and that traditional lands offer an outlet to reduce stress from daily pressures, being described somewhat like a utopian sanctuary (Kingsley et al., 2009).

In a paper by Burgess et al (2008) it is quoted that "Our identity as human beings remains tied to our land, to our cultural practices, our systems of authority and social control, our intellectual traditions, our concepts of spirituality, and to our systems of resource ownership and exchange. Destroy this relationship and you damage – sometimes irrevocably – individual human beings and their health". A paper by Kingsley et al. (2009) makes reference to a poignant quote from a Yorta Yorta traditional custodian “people who don’t have this strong identity to land are less than what they can be, leading them to drugs, alcohol, or domestic violence because they can’t find it within themselves” (Kingsley et al., 2009).

There is a growing recognition that Indigenous community-based involvement in natural resource management can bring significant economic and socio-cultural benefits (Altman and Whitehead, 2003). The inherent socio-cultural benefits for Aboriginal people of engagement in natural resource management activities on country include positive outcome such as living on country, remote from access to alcohol and other potential negative influences and a more positively living a lifestyle that promotes spiritual and physical well-being (Altman and Whitehead, 2003). Other reviews of evidence have concluded that engagement of Aboriginal people with land management can enable people to feel that their actions are consistent with their own sense of the right and proper way for them to behave towards land, family and community (Davies et al., 2011). Similarly Johnson (2007) remarks on Indigenous people who have described the relief and rejuvenation of returning to country even if for brief visits, burn country, to hunt and gather food, educate young people and maintain cultural and spiritual obligations (Johnston et al., 2007).

For many Aboriginal peoples natural resource management is caring for country as it “embodies deep spiritual obligations and patterns of behaviour proscribed by enduring metaphysical associations with geography”(Burgess et al., 2005). The Indigenous notion of caring for country is defined as Indigenous participation in interrelated activities with the objective of promoting ecological and human health (Burgess et al., 2008). In a later paper Burgess also adds that caring for country is a “community driven movement towards long-term social, cultural, physical, and sustainable economic development in rural and remote locations, simultaneously contributing to the conservation of globally valued environmental and cultural assets” (Burgess et al., 2009).

In a report by Burgess and Johnston of the preliminary findings of a Healthy Country: Healthy People project, the health benefits of participants in Natural and Cultural Resource Management (NCRM) or Caring for Country versus non-participants was examined. The cross sectional survey found that:

- Indigenous NCRM is seen as an important determinant of landscape and human health;
- Higher levels of participation in Indigenous NCRM may be associated with significantly better health outcomes;

Preliminary results from the study indicated that higher levels of NCRM participation were associated with better outcomes across a broad array of risk factors linked to diabetes and cardiovascular risk. The study showed that participants in NCRM self-report a more nutritious diet and greater levels of physical activity (Burgess and Johnston, 2007).

**Physical and mental health benefits**

In a study conducted with a remote Australian Arnhem land community Burgess found a significant and substantial association between greater participation in caring for country activities and lower body mass index was demonstrated (Burgess et al., 2008). The study found with adjustment for socio-demographic factors and health behaviours, an inter-quartile range rise in those individuals involved with caring for country with scores that were associated with 6.1 kg for non-pregnant women and 5.3 kg less body weight for men.

The study found that participation in caring for country activities was significantly associated with less frequent consumption of takeaway and more frequent consumption of bush food and greater physical activity, all of these being health behaviours that contribute to less obesity. Burgess concludes stating that the study provides empirical epidemiological support for long-standing Indigenous demands for institutional investment in managing their country and that such investment may have substantial health and cultural benefits for Australia's most disadvantaged and dispossessed peoples (Burgess et al., 2008).

In a cross-sectional study reported by Garnett and Sithole (2007) increasing self-reported participation in Indigenous Natural and Cultural Resource Management (ICNRM) was found to be associated with a range of health benefits over broad range of risk factors and disease endpoints. It was found that increasing participation in ICNRM was associated with a more nutritious diet and greater levels of physical activity with risk factors for developing diabetes and cardiovascular disease showing significant inverse associations with ICNRM participation, including the body mass index (BMI). The study also revealed that the population prevalence of Non-Insulin Dependent Diabetes Mellitus (NIDDM) which is associated with increased cardiovascular risk, kidney failure, blindness, ulcers and limb amputations, showed a significant inverse association with increasing ICNRM participation. The project findings concluded that the health outcomes associated with ICNRM can in fact help prevent or delay significant causes of premature disease and death, delivering significant economic savings in health care expenditure. In alter paper (Garnett et al., 2009) state that ICNRM "draws on a substantial reservoir of community strengths from which improved primary and secondary prevention outcomes could be obtained."

Similar studies such as those by McDermott et al (2008) have reported Indigenous people living on their traditional land, rather than in urban areas, having lower rates of diabetes, cardiovascular disease, mortality and morbidly rates (McDermott et al., 2008).Ganesharajah (2009) goes on to say that Indigenous people engaged in valued services, such as ICNRM, can use their service delivery as a type of bargaining tool or a form of leverage and that this can at least go some way towards reducing Indigenous peoples' vulnerability to power inequalities and also to increasing autonomy which is an important determinant of health.

A study reported by Weir (2009) in the Murray Region in south-eastern Australia, it was reported that Aboriginal people have attributed aspects of their own poor physical or mental health to the poor health of the Murray River. The study suggests that due to environmental degradation of the river, together with legal restrictions on access,

Aboriginal people were unable to pass on traditional knowledge or undertake traditional activities that were closely connected with the river system and that this change in activity had negative impacts on Indigenous people's self-assessed physical and mental health (Weir et al., 2011).

#### 7.4.1 Citations

Altman, J.C., Whitehead, P.J., 2003. Caring for country and sustainable Indigenous development: opportunities, constraints and innovation (CAEPR Working Paper No. 20). Centre for Aboriginal Economic Policy Research.

Burgess, C., Berry, H., Gunthorpe, W., Bailie, R., 2008. Development and preliminary validation of the "Caring for Country" questionnaire: measurement of an Indigenous Australian health determinant. International Journal for Equity in Health 7, 26.

Burgess, C.., Johnston, F.H., 2007. Healthy Country Healthy People. Indigenous natural and Cultural Resource Management and Health.

Burgess, C.P., Johnston, F.H., Berry, H.L., McDonnell, J., Yibarbuk, D., Gunabarra, C., Mileran, A., Bailie, R.S., 2009. Healthy country, healthy people: the relationship between Indigenous health status and "caring for country". Med. J. Aust. 190.

Burgess, C.P., Johnston, F.H., Bowman, D.M.J.S., Whitehead, P.J., 2005. Healthy country: healthy people? Exploring the health benefits of indigenous natural resource management. Aust N Z J Public Health 29, 117–122.

Cannon, R., 2008. The social determinants of health (SACOSS Information Paper). South Australian Council of Social Service, Unley, SA.

Davies, J., Campbell, D., Campbell, M., Douglas, J., Hueneke, H., LaFlamme, M., Pearson, D., Preuss, K., Walker, J., Walsh, F., 2011. Attention to four key principles can promote health outcomes from desert Aboriginal land management. The Rangeland Journal 33, 417–431.

De Vries, S., Verheij, R.A., Groenewegen, P.P., Spreeuwenberg, P., 2003. Natural environments-healthy environments? An exploratory analysis of the relationship between greenspace and health. Environment and planning A 35, 1717–1732.

Ganesharajah, C., 2009. Indigenous health and wellbeing: The importance of country (Native title Research Report No. 1/2009). Australian Institute of Aboriginal and Torres Strait Islander Studies, Canberra, ACT.

Garnett, S.T., Sithole, B., 2007. Sustainable northern landscapes and the nexus with Indigenous health: Healthy country, healthy people ( No. NTU7). Land & Water Australia, Canberra, ACT.

Garnett, S.T., Sithole, B., Whitehead, P.J., Burgess, C.P., Johnston, F.H., Lea, T., 2009. Healthy country, healthy people: Policy implications of links between Indigenous human health and environmental condition in tropical Australia. Australian Journal of Public Administration 68, 53–66.

Gooch, M., 2003. A sense of place: Ecological identity as a driver for catchment volunteering. Australian Journal on Volunteering 8, 23–32.

Johnston, F., Burgess, P., Bowman, D.M.J.S., 2007. Chapter 15 - A case for Indigenous natural resource management and health, in: Luckert, M.K., Campbell, B.M., Gorman, J.T., Garnett, S.T. (Eds.), Investing in Indigenous Natural Resource Management. Charles Darwin University Press, Darwin, NT.

Kingsley, J., Townsend, M., Phillips, R., Aldous, D., 2009. "If the land is healthy … it makes the people healthy": The relationship between caring for Country and health for the Yorta Yorta Nation, Boonwurrung and Bangerang Tribes. Health & Place 15, 291–299.

Koss, R.S., Kingsley, J.Y., 2010. Volunteer health and emotional wellbeing in marine protected areas. Ocean & Coastal Management 53, 447–453.

Maller, C., Townsend, M., Brown, P., St Leger, L., 2002. Healthy parks healthy people: The health benefits of contact with nature in a park context. Burwood (Australia): Faculty of Health and Behavioural Sciences, Deakin University.

Maller, C., Townsend, M., St Leger, L., Henderson-Wilson, C., Pryor, A., Prosser, L., Moore, M., 2008. Healthy parks, healthy people. The health benefits of contact with nature in a park context. A review of relevent literature., 2nd edition. Melbourne, Victoria.

McDermott, R., O'Dea, K., Rowley, K., Knight, S., Burgess, P., 2008. Beneficial impact of the Homelands Movement on health outcomes in central Australian Aborigines. Australian and New Zealand Journal of Public Health 22, 653–658.

Pretty, G., Bishop, B., Fisher, A., Sonn, C., 2007. Psychological sense of community and its relevance to well-being and everyday life in Australia. The Australian Community Psychologist 19, 6–25.

Putnam, R.D., 1993. The prosperous community. The American prospect 4, 35–42.

Townsend, M., Weerasuriya, R., 2010. Beyond blue to green: The benefits of contact with nature for mental health and well-being. Beyond Blue Limited, Melbourne, Victoria.

Weir, J.K., Stacey, C., Youngetob, K., 2011. The benefits of caring for country (Report for the Department of Sustainability, Environment, Water, Population and Communities). Australian Institute of Aboriginal and Torres Strait Islander Studies, Canberra, ACT.

**7.5 Resilience**

Resilient social-ecological systems are defined as having the ability to create networks that facilitate learning in order to solve problems in times of change without significant losses in crucial functions, such as productivity, environmental, or economic losses (Folke et al. 2002). Although there are many definitions of resilience, what is common to most of them is the notion of overcoming adversity (Buikstra et al., 2010). Through the multiple benefits of Landcare Networks, participating communities have been successful in building the resilience and adaptive capacity of their social-ecological systems.

Landcare promotes the formation of networks that allow communities to support each other, increasing social cohesion through incorporating social, economic, environmental and cultural considerations into Landcare activities (Colliver, 2011; Landcare Victoria and Department of Sustainability and Environment, 2008a; Love, 2012). This has been accomplished through Landcare group's ability to involve a range of landholders and community members in order to deliver market-based instruments, integrate actions with NRM priorities, and engage with corporate companies in environmental project work (Beilin and Reichelt, 2010; Curtis et al., 1999). As a result of this enhanced adaptive capacity, Landcare has evolved to be able to address system-wide issues such as "climate change, sustainable farming, biodiversity loss and urban growth" and ultimately create stronger, healthier communities that are better prepared to cope with change (Landcare Victoria and Department of Sustainability and Environment, 2008).

A participatory study based in Stanthorpe, Queensland, found that environmental factors such as connection to the land and a strong sense of community influences the overall resilience of the individual, which subsequently contributes to the resilience of the community and the entire socio-ecological system (Hegney et al., 2008).

The study identified eleven major concepts that enhance resilience, which were:

- Social networks and support
- Positive outlook
- Learning
- Early experience
- Environment and lifestyle
- Infrastructure and support services
- Sense of purpose
- Diverse and innovative economy
- Embracing differences
- Beliefs and leadership,

In another study of rural community resilience conducted in Stanthorpe in southern Queensland, 72 participants from six different sectors (i.e. service providers, commercial, farming etc) were interviewed to identify and explore what makes up community and

individual resilience found that all study groups identified the presence of social networks and support was seen as a critical resilience factor and a key element of community resilience and an ideal resilient community (Buikstra et al., 2010)

Many participants from the interviews also emphasized the role of the natural environment and climate in shaping Stanthorpe's resilience. The features of the natural environment were associated with feelings of well-being for some participants. It was reported that learning to cope with climatic events, such as drought, frost, and hail had built resilience among Stanthorpe's farmers. Many participants responded that the appeal of the region's natural environment and informal rural lifestyle was critical in Stanthorpe's resilience and would be found in an ideal resilient community. The natural environment was also associated with community pride and a sense of belonging (Buikstra et al., 2010).

Landcare programs currently support these central concepts needed to maintain resilient socio-ecological systems with the help of community-based NRM networks (Hegney et al., 2008).

The 2008 Victorian Landcare Forum reported that participation in and the philosophy of Landcare can lead to innovation and the adoption of new technologies in order to increase production and enhance the sustainability of our actions (Landcare Victoria and Department of Sustainability and Environment, 2008). Increasing the skills base of Landcare farmers can therefore be attributed to adaptability and resilience of the socio-ecological system. This demonstrates that community based Landcare is a successful NRM platform through its ability to help rural communities engage with their socio-ecological system and enhance community-wide learning, and therefore resilience (Beilin and Reichelt, 2010). By enhancing socio-ecological resilience, Landcare has also been able to support adaptive management when stakeholder engagement is not sufficient (Curtis et al., 2000). With the inherent limitations of stakeholders, Landcare is able to support adaptive management and socio-ecological resilience when external support is insufficient (Curtis et al., 2000).

#### 7.5.1 Citations

Altman, J.C., Whitehead, P.J., 2003. Caring for country and sustainable Indigenous development: opportunities, constraints and innovation (CAEPR Working Paper No. 20). Centre for Aboriginal Economic Policy Research.

Beilin, R., Reichelt, N., 2010. Community Landcare: a key player in building social-ecological resilience networks? The State of Victoria Department of Sustainability and Environment, Melbourne.

Brown, V., 1997. Landcare in Australia: talking local sustainability in policy, practice and place (RDFN Paper No. 20e). Rural Development Forestry Network, London, UK.

Buikstra, E., Ross, H., King, C.A., Baker, P.G., Hegney, D., McLachlan, K., Rogers-Clark, C., 2010. The components of resilience - perceptions of an Australian rural community. Journal of Community Psychology 38, 975–991.

Campbell, A., 1992. Taking the long view in tough times: Landcare in Australia (Third Annual Report), National Landcare Facilitator. National Soil Conservation Program.

Campbell, A., 1995a. Land literacy in Australia. Journal of Soil and Water Conservation 126–131.

Campbell, C.A., 1995b. Landcare: Participative Australian approaches to inquiry and learning for sustainability. Journal of Soil and Water Conservation 50, 125–131.

Campbell, C.A., 2009. It's time to renew Landcare. Agricultural Science 30–33.

Cary, J., Webb, T., 2000. Community landcare, the National Landcare Program and the landcare movement: the social dimensions of landcare. Bureau of Rural Science, Canberra, ACT.

Cary, J., Webb, T., 2001. Landcare in Australia: Community participation and land management. Journal of Soil and Water Conservation 56, 274–278.

Catacutan, D., Neely, C., Johnson, M., Youl, R. (Eds.), 2009. Landcare: Local Action - Global Progress. World Agroforestry Centre, Nairobi, Kenya.

Colliver, R., 2011. Community-based governance in social-ecological systems: An inquiry into the marginalisation of Landcare in Victoria, Australia (PhD of Philosophy Thesis).

Compton, E., Hayne, W., Beeton, R.J.S., 2007. Six years in a cement mixer: A landcare journey through changing times. Presented at the International Landcare Conference, Melbourne, Victoria.

Coree Consulting Pty Ltd, 2003. Landcare Literature Review. Coree Consulting Pty Ltd.

Curtis, A., 1995a. Landcare in Australia: A critical review (report No. 33). Charles Sturt University, Albury, NSW.

Curtis, A., 1995b. Landcare in Victoria: The state of play (Report for Landcare Victoria No. 24). Johnstone Centre, Charles Sturt University, Albury, NSW.

Curtis, A., 1999. Landcare in Victoria: Beyond onground work (Report No. 125). Johnstone Centre, Charles Sturt University, Albury, NSW.

Curtis, A., 2003. Reflecting on the Landcare experience: A report based on information held within ABARE and BRS. Bureau of Rural Sciences.

Curtis, A., Britton, A., Sobels, J., 1999. Landcare networks in Australia: State-sponsored participation through local organizations. Journal of Environmental Planning and Management 42, 5–21.

Curtis, A., Cooke, P., 2006. Landcare groups in Victoria: After twenty years (Report to the Australian Government Department of Agriculture, Fisheries and Forestry, National Landcare Program, Monitoring and Evaluation Project). Institute for Land, Water and Society, and the National Landcare Program, Albury, NSW.

Curtis, A., De Lacy, T., 1995. Evaluating landcare groups in Australia: How they facilitate partnerships between agencies, community groups, and researchers. Journal of Soil and Water Conservation January-February, 15–20.

Curtis, A., De Lacy, T., 1996a. Landcare in Australia: Does it make a difference? Journal of Environmental Management 46, 119–137.

Curtis, A., De Lacy, T., 1996b. Landcare in Australia: Beyond the expert farmer. Agriculture and Human Values 13, 20–32.

Curtis, A., De Lacy, T., 1996c. Landcare in Australia: Does it make a difference? Johnstone Centre, Charles Sturt University, Albury, NSW.

Curtis, A., De Lacy, T., 1998. Landcare, stewardship and sustainable agriculture in Australia. Environmental Values 7, 59–78.

Curtis, A., De Lacy, T., Klomp, N., 1993. Asessing the effectiveness of landcare groups: The Victorian experience. Regional Journal of Social Issues 27, 78–91.

Curtis, A., Lockwood, M., 2000. Landcare and catchment management in Australia: Lessons for state-sponsored community participation. Society & Natural Resources 13, 61–73.

Curtis, A., Lucas, D., Nurse, M., Skeen, M., 2008. Achieving NRM outcomes through voluntary action: Lessons from Landcare (Discussion Paper). Charles Sturt University & Victorian Department of Sustainability and Environment, Melbourne, Victoria.

Curtis, A., Sample, R., 2010. CBNRM in Victoria: Contributing to dialogue, learning and action (ILWS Report No. 55). Institute for Land, Water and Society, Charles Sturt University, Albury, NSW.

Curtis, A., Van Nouhuys, M., Robinson, W., MacKay, J., 2000. Exploring Landcare effectiveness using organisational theory. Australian Geographer 31, 349–366.

Department of Agriculture, 2003. Review of the National Landcare Program (Report). Department of Agriculture, Canberra, ACT.

Dietz, T., Stern, P.C. (Eds.), 2002. New Tools for Environmental Protection: Education, Information, and Voluntary Measures. The National Academies Press, Washington, D.C.

Edmonson, R., 2010. Reviewing Landcare.

Garnett, S.T., Sithole, B., Whitehead, P.J., Burgess, C.P., Johnston, F.H., Lea, T., 2009. Healthy country, healthy people: Policy implications of links between Indigenous human health and environmental condition in tropical australia. Australian Journal of Public Administration 68, 53–66.

Gooch, M.J., 2004. Voices of the volunteers: an exploration of the influences that volunteer experiences have on the resilience and sustainability of catchment groups in coastal queensland (PhD Thesis).

Griffin NRM, URS Australia Pty Ltd, 2001. An evaluation of investment in Landcare support projects (Final report for Agriculture, Fisheries and Forestry Australia). URS Australia Pty Ltd and Griffin NRM.

Gunningham, N., 2002. Reconfiguring Environmental Regulation: The Future Public Policy Agenda. N. Gunningham & D. Sinclair ds., Leaders and Laggards: Ne t Generation nvironmental Regulation pp.

Gunningham, N., Kagan, R.A., Thornton, D., 2004. Social license and environmental protection: why businesses go beyond compliance. Law & Social Inquiry 29, 307–341.

Gunningham, N., Sinclair, D., 1998. Designing smart regulation, in: Economic Aspects of Environmental Compliance Assurance. OECD Global Forum on Sustainable Development.

Hamilton, C., 1995. The economic language of Landcare (Background Paper No. 3). The Australia Institute, Canberra, ACT.

Hawthorne, M., Alabaster, T., 1999. Citizen 2000: development of a model of environmental citizenship. Global Environmental Change 9, 25–43.

Hegney, D., Ross, H., Baker, P., Buikstra, E., King, C., McLachlan, K., Rogers-Clark, C., Stallard, L., Watson-Luke, A., 2008. Identification of personal and community resilience that enhance psychological wellness: A Stanthorpe study. Australian Research Council, Canberra, ACT.

Hogan, L., Cumming, B., 1997. More than a question of numbers: Working with women and people from a non-English speaking background toward total catchment participation (Report of the Landcare Participation Project). Department of Primary Industries and Energy, and the Department of Natural Resources and Environment, Canberra, ACT.

Horvath, D. (Ed.), 2001. NSW Decade of Landcare Evaluation. NSW Department of Land and Water Conservation, Sydney, NSW.

Landcare Victoria, Department of Sustainability and Environment, 2008a. Shaping our future: Proceedings. Presented at the 2008 Victorian Landcare Forum, Landcare Victoria, Creswick, Victoria, p. 21.

Landcare Victoria, Department of Sustainability and Environment, 2008b. Shaping our future: Proceedings. Presented at the 2008 Victorian Landcare Forum, Landcare Victoria, Creswick, Victoria, p. 28.

Landcare Victoria, Department of Sustainability and Environment, 2008c. Shaping our future: Stories. Presented at the 2008 Victorian Landcare Forum, Landcare Victoria, Creswick, Victoria, p. 42.

Landcare Victoria, Department of Sustainability and Environment, 2008d. Shaping our future: Stories. Presented at the 2008 Victorian Landcare Forum, Landcare Victoria, Creswick, Victoria, p. 34.

Lockie, S., 1998. Landcare in Australia: Cultural transformation in the management of rural environments. Culture & Agriculture 20, 21–29.

Love, C., 2012. Evolution of Landcare in Australia: In the context of Australian Government natural resource management policy and programs (Perspectives from the former National Landcare Facilitator). Australian Landcare Council Secretariat.

MacGregor, S., Pardoe, S., Dobson, A., Bell, D., 2005a. Environmental citizenship: the Goodenough primer (summary report of an interdisciplinary seminar series) (Summary Report). The Open University, Milton Keynes, UK.

MacGregor, S., Pardoe, S., Dobson, A., Bell, D., 2005b. Environmental citizenship: the Goodenough primer (summary report of an interdisciplinary seminar series) (Summary Report). The Open University, Milton Keynes, UK.

McTernan, R., Scully, M.-A., 2010. Finding common ground: A review of Indigenous engagement in Landcare in Victoria. Victorian Government Department of Sustainability and Environment, Melbourne, Victoria.

Mues, C., Chapman, L., Van Hilst, R., 1998. Promoting improved land management practices on Australian farms: A survey of landcare and land management related programs (Research report No. 98.4), Landcare. Australian Bureau of Agricultural and Resource Economics, Canberra, ACT.

Mullen, J.D., 2001. An economic perspective on land degredation issues (Economic Research Report No. 9). NSW Agriculture, Orange, NSW.

Nicholson, C., Knight, A., 2003. Auditing 10 years of Landcare in the Woady Yaloak catchment: How are we progressing along the triple bottom line? Presented at the National Landcare Conference, Darwin, NT, p. 7.

Quealy, J., 1998. Landcare: Rural Landcare. Landcare New South Wales, Sydney, NSW.

Sobels, J., Curtis, A., 2001a. Landcare networks: Establishing viable local organisations in rural Australia. Natural Resource Management 4, 22–30.

Sobels, J., Curtis, A., 2001b. Second generation Landcare support in West Gippsland (report for Landcare Victoria No. 156). Johnstone Centre, Charles Sturt University, Albury, NSW.

Sobels, J., Curtis, A., Lockie, S., 2001. The role of Landcare group networks in rural Australia: Exploring the contribution of social capital. Journal of Rural Studies 17, 265–276.

Stern, P.C., 2000a. Toward a coherent theory of environmentally significant behaviour. Journal of Social Issues 56, 407–424.

Stern, P.C., 2000b. New environmental theories: Toward a coherent theory of environmentally significant behavior. Journal of social issues 56, 407–424.

Toyne, P., Farley, R., 2000. The decade of Landcare: Looking backward - looking forward (Discussion Paper No. 30). The Australia Institute.

Vanclay, F.M., Lockie, S. (Eds.), 2000. Critical Landcare, Key papers series. Centre for Rural Social Research, Charles Sturt University, Wagga Wagga, NSW.

Walker, R.H., 2000. The National Landcare Program: What has it changed? (A compilation of evaluations of the National Landcare Program 1992-1998). Department of Agriculture, Fisheries and Forestry, Canberra, ACT.

Webb, T., Cary, J., 2005. Social capital and natural resource management: An application to Landcare. Rural Society 15, 119–131.

Woodhill, J., 1992. Landcare in NSW, taking the next step! Final report and recommendations for the development of Landcare in NSW from the 1990/91 (Final report for the National Soil Conservation Program). Department of Conservation and Land Management, Chatswood, NSW.

Youl, R. (Ed.), 2006. Landcare in Victoria: How Landcare Helped People, Government and Business Work Together in Victoria - a Collection. R. Youl, South Melbourne, Victoria.

Youl, R., Marriott, S., Nabben, T., 2006. Landcare in Australia: Founded on Local Action, 5th ed. SILC, Melbourne, Victoria.

# Appendices

## 8. Appendix 1: Literature Cited or References

Altman, J.C., Whitehead, P.J., 2003. Caring for country and sustainable Indigenous development: opportunities, constraints and innovation (CAEPR Working Paper No. 20). Centre for Aboriginal Economic Policy Research.

Ashby, J., 2003. Introduction: Uniting Science and Participation in the Process of Innovation–Research for Development.

Baker, R., 1997. Landcare: policy, practice and partnerships. Australian Geographical Studies 35, 61–73.

Bammer, G., O'Connell, D., Roughley, A., Syme, G., 2005. Integration research for natural resource management in Australia: An introduction to new challenges for research practice. Journal of Research Practice 1, Article–E1.

Barr, N., Cary, J., 2000. Influencing improved natural resource management on farms. Bureau of Rural Sciences, Canberra.

Beeton, S., 2002. Entrepreneurship in rural tourism? Australian Landcare programs as a destination marketing tool. Journal of Travel Research 41, 206–209.

Beilin, R., 1995. The construction of woman in Landcare: Does it make a difference? Rural Society 5, 20–29.

Beilin, R., Reichelt, N., 2010. Community Landcare: a key player in building social-ecological resilience networks? The State of Victoria Department of Sustainability and Environment, Melbourne.

Bradshaw, B., 2003. Questioning the credibility and capacity of community-based resource management. The Canadian Geographer/Le Géographe canadien 47, 137–150.

Broderick, K., 2005. Communities in catchments: implications for natural resource management. Geographical Research 43, 286–296.

Brown, V., 1997. Landcare in Australia: talking local sustainability in policy, practice and place (RDFN Paper No. 20e). Rural Development Forestry Network, London, UK.

Brown, V.A. (Ed.), 1996. Landcare Languages: Talking to each other about living with the land. Department of Primary Industries and Energy, Canberra, ACT.

Brumby, S., Willder, S., Martin, J., 2010. Milking their health for all its worth? Improving the health of farming families through facilitated learning. Extension Farming Systems Journal 6, 1–11.

Buchy, M., Race, D., 2001. The twists and turns of community participation in natural resource management in Australia: what is missing? Journal of Environmental Planning and Management 44, 293–308.

Buikstra, E., Ross, H., King, C.A., Baker, P.G., Hegney, D., McLachlan, K., Rogers-Clark, C., 2010. The components of resilience - perceptions of an Australian rural community. Journal of Community Psychology 38, 975–991.

Bunch, M.J., Morrison, K.E., Parkes, M.W., Venema, H.D., 2011. Promoting health and well-being by managing for social-ecological resilience: The potential of integrating ecohealth and water resources. Ecology and Society 16, 6.

Burgess, C., Berry, H., Gunthorpe, W., Bailie, R., 2008. Development and preliminary validation of the "Caring for Country" questionnaire: measurement of an Indigenous Australian health determinant. International Journal for Equity in Health 7, 26.

Burgess, C.., Johnston, F.H., 2007. Healthy Country Healthy People. Indigenous natural and Cultural Resource Management and Health.

Burgess, C.P., Johnston, F.H., Berry, H.L., McDonnell, J., Yibarbuk, D., Gunabarra, C., Mileran, A., Bailie, R.S., 2009. Healthy country, healthy people: the relationship between Indigenous health status and "caring for country". Med. J. Aust. 190.

Burgess, C.P., Johnston, F.H., Bowman, D.M.J.S., Whitehead, P.J., 2005. Healthy country: healthy people? Exploring the health benefits of indigenous natural resource management. Aust N Z J Public Health 29, 117–122.

Campbell, A., 1990. Landcare: Progress across the nation (First Annual Report). National Soil Conservation Program.

Campbell, A., 1992. Taking the long view in tough times: Landcare in Australia (Third Annual Report), National Landcare Facilitator. National Soil Conservation Program.

Campbell, A., 1994. Community First: Landcare in Australia, Gatekeeper Series. Sustainable Agriculture Programme of the International Institute for Environmental Development, London, UK.

Campbell, A., 1995a. Land literacy in Australia. Journal of Soil and Water Conservation 126–131.

Campbell, A., 1995b. Facilitating Landcare: Conceptual and practical dilemmas. Rural Society 5, 13–19.

Campbell, A., 1998. Issues in Landcare, in: Vanclay, F., Lockie, S. (Eds.), Critical Landcare. University of Central Queensland Press, p. 8.

Campbell, A., Siepen, G., 1994. Landcare: Communities Shaping the Land and the Future. Allen & Unwin.

Campbell, B., Sayer, J.A., Frost, P., Vermeulen, S., Pérez, M.R., Cunningham, A., Prabhu, R., 2001. Assessing the performance of natural resource systems. Conservation Ecology 5, 22.

Campbell, C.A., 1995c. Landcare: Participative Australian approaches to inquiry and learning for sustainability. Journal of Soil and Water Conservation 50, 125–131.

Campbell, C.A., 2009. It's time to renew Landcare. Agricultural Science 30–33.

Cannon, R., 2008. The social determinants of health (SACOSS Information Paper). South Australian Council of Social Service, Unley, SA.

Carr, A., 1995. Innovation of diffusion: Landcare and information exchange. Rural Society 5, 56–66.

Cary, J., Webb, T., 2000. Community landcare, the National Landcare Program and the landcare movement: the social dimensions of landcare. Bureau of Rural Science, Canberra, ACT.

Cary, J., Webb, T., 2001. Landcare in Australia: Community participation and land management. Journal of Soil and Water Conservation 56, 274–278.

Catacutan, D., Neely, C., Johnson, M., Youl, R. (Eds.), 2009. Landcare: Local Action - Global Progress. World Agroforestry Centre, Nairobi, Kenya.

Cheng, A.S., Kruger, L.E., Daniels, S.E., 2003. "Place" as an integrating concept in natural resource politics: Propositions for a social science research agenda. Society & Natural Resources 16, 87–104.

Colliver, R., 2011. Community-based governance in social-ecological systems: An inquiry into the marginalisation of Landcare in Victoria, Australia (PhD of Philosophy Thesis).

Colliver, R., Lucas, D., Moore, S., 2008. Agent, partner or activist: the role of community Landcare in creating sustainable rural landscapes, in: Community Development and Ecology: Engaging Ecological Sustainability Through Community Development. Presented at the International Eco Community Conference, Murdoch University, Melbourne, Victoria, p. 12.

Comerford, E., 2004. Choosing between incentive mechanisms for natural resource management. A practical guide for regional NRM bodies in Queensland. Queensland Department of Natural Resources and Mines, Brisbane, Queensland.

Compton, E., Beeton, R.J.S., 2012. An accidental outcome: Social capital and its implications for Landcare and the "status quo". Journal of Rural Studies 28, 149–160.

Compton, E., Hayne, W., Beeton, R.J.S., 2007. Six years in a cement mixer: A landcare journey through changing times. Presented at the International Landcare Conference, Melbourne, Victoria.

Compton, E., Prager, K., Beeton, B., 2009. Landcare bowling alone: Finding a future in the "fourth" phase, in: Lane, M., Robinson, C., Taylor, B. (Eds.), Contested Country: Local and Regional Natural Resource Management in Australia. CSIRO, Collingwood, Victoria, pp. 147–160.

Cooke, P.R., 2010. Education in Landcare groups: Social learning aspects of adaptive management. Extension Farming Systems Journal 6, 139–144.

Coree Consulting Pty Ltd, 2003. Landcare Literature Review. Coree Consulting Pty Ltd.

Curtis, A., 1995a. Landcare in Victoria: The state of play (Report for Landcare Victoria No. 24). Johnstone Centre, Charles Sturt University, Albury, NSW.

Curtis, A., 1995b. Landcare in Australia: A critical review (report No. 33). Charles Sturt University, Albury, NSW.

Curtis, A., 1998. Agency-community partnership in Landcare: Lesons for state-sponsored citizen resource management. Environmental Management 22, 563–574.

Curtis, A., 1999. Landcare in Victoria: Beyond onground work (Report No. 125). Johnstone Centre, Charles Sturt University, Albury, NSW.

Curtis, A., 2003. Reflecting on the Landcare experience: A report based on information held within ABARE and BRS. Bureau of Rural Sciences.

Curtis, A., Birckhead, J., De Lacy, T., 1995. Community participation in Landcare policy in Australia: The Victorian experience with regional Landcare plans. Society and Natural Resources 8, 415–430.

Curtis, A., Britton, A., Sobels, J., 1999. Landcare networks in Australia: State-sponsored participation through local organizations. Journal of Environmental Planning and Management 42, 5–21.

Curtis, A., Cooke, P., 2006. Landcare groups in Victoria: After twenty years (Report to the Australian Government Deparment of Agriculture, Fisheries and Forestry, National Landcare Program, Monitorin and Evaluation Project). Instutute for Land, Water and Society, and the National Landcare Program, Albury, NSW.

Curtis, A., Davidson, P., De Lacy, T., 1997. Women's participation and experience of Landcare in Australia. Journal of Sustainable Agriculture 10, 37–56.

Curtis, A., De Lacy, T., 1995a. Evaluating landcare groups in Australia: How they facilitate partnerships between agencies, community groups, and researchers. Journal of Soil and Water Conservation January-February, 15–20.

Curtis, A., De Lacy, T., 1995b. Examining the assumptions underlying Landcare. Rural Society 44–55.

Curtis, A., De Lacy, T., 1996a. Landcare in Australia: Beyond the expert farmer. Agriculture and Human Values 13, 20–32.

Curtis, A., De Lacy, T., 1996b. Landcare in Australia: Does it make a difference? Journal of Environmental Management 46, 119–137.

Curtis, A., De Lacy, T., 1996c. Landcare in Australia: Does it make a difference? Johnstone Centre, Charles Sturt University, Albury, NSW.

Curtis, A., De Lacy, T., 1998. Landcare, stewardship and sustainable agriculture in Australia. Environmental Values 7, 59–78.

Curtis, A., Lockwood, M., 2000. Landcare and catchment management in Australia: Lessons for state-sponsored community participation. Society & Natural Resources 13, 61–73.

Curtis, A., Lucas, D., Nurse, M., Skeen, M., 2008. Achieving NRM outcomes through voluntary action: Lessons from Landcare (Discussion Paper). Charles Sturt University & Victorian Department of Sustainability and Environment, Melbourne, Victoria.

Curtis, A., Sample, R., 2010. CBNRM in Victoria: Contributing to dialogue, learning and action (ILWS Report No. 55). Institute for Land, Water and Society, Charles Sturt University, Albury, NSW.

Curtis, A., Van Nouhuys, M., 1999. Landcare participation in Australia: The volunteer perspective. Sustainable Development 7, 98–111.

Curtis, A., Van Nouhuys, M., Robinson, W., MacKay, J., 2000. Exploring Landcare effectiveness using organisational theory. Australian Geographer 31, 349–366.

Department of Agriculture, 2003. Review of the National Landcare Program (Report). Department of Agriculture, Fisheries and Forestry, Canberra, ACT.

Davies, J., Campbell, D., Campbell, M., Douglas, J., Hueneke, H., LaFlamme, M., Pearson, D., Preuss, K., Walker, J., Walsh, F., 2011. Attention to four key principles can promote health outcomes from desert Aboriginal land management. The Rangeland Journal 33, 417–431.

Davies, J., Campbell, D., Campbell, M., Douglas, J., Hueneke, H., LaFlamme, M., Preuss, K., Walker, J., Walsh, F., 2010. Livelihoods inLand: Promoting health and wellbeing outcomes from desert Aboriginal land management (DKCRC Report No. 78). Ninti One Limited, Alice Springs.

De Vries, S., Verheij, R.A., Groenewegen, P.P., Spreeuwenberg, P., 2003. Natural environments-healthy environments? An exploratory analysis of the relationship between greenspace and health. Environment and planning A 35, 1717–1732.

Department of Sustainability and Environment, 2008. Landcare in Victoria: Community participation and perceptions research (Final Report). Department of Sustainability and Environment, East Melbourne, Victoria.

Dietz, T., Stern, P.C. (Eds.), n.d. New Tools for Environmental Protection: Education, Information, and Voluntary Measures. National Academy Press, Washington, DC.

Dyack, B., Greiner, R., 2006. Natural resource management and Indigenous well being, in: 50th Annual Conference of the Australian Agricultural and Resource Economics Society. Sydney, NSW, p. 31.

Edmonson, R., 2010. Reviewing Landcare.

Emtage, N., Herbohn, J., Harrison, S., 2006. Landholder typologies used in the development of Natural Resource Management programs in Australia–a review. Australasian Journal of Environmental Management 13, 79–94.

Everingham, J., Cheshire, L., Lawrence, G., 2006. Regional renaissance? New forms of governance in nonmetropolitan Australia. Environment and Planning C 24, 139.

Falk, I., Harrison, L., 1998. Community learning and social capital: "just having a little chat". Journal of Vocational Education and Training 50, 609–627.

Ganesharajah, C., 2009. Indigenous health and wellbeing: The importance of country (Native title Research Report No. 1/2009). Australian Institute of Aboriginal and Torres Strait Islander Studies, Canberra, ACT.

Garnett, S.T., Sithole, B., 2007. Sustainable northern landscapes and the nexus with Indigenous health: Healthy country, healthy people ( No. NTU7). Land & Water Australia, Canberra, ACT.

Garnett, S.T., Sithole, B., Whitehead, P.J., Burgess, C.P., Johnston, F.H., Lea, T., 2009. Healthy country, healthy people: Policy implications of links between Indigenous human health and environmental condition in tropical australia. Australian Journal of Public Administration 68, 53–66.

Gill, N., 2004. Politics within and without — the origins and development of a rangelands Landcare group. Australian Geographical Studies 42, 135–151.

Gleeson, T., Heilbron, S., Hudson, B., Douglas, J., 2006. Capturing market and other benefits from improved land management (Report for National Heritage Trust). Australian Landcare Management System, South Brisbane, Queensland.

Gooch, M., 2003. A sense of place: Ecological identity as a driver for catchment volunteering. Australian Journal on Volunteering 8, 23–32.

Gooch, M.J., 2004. Voices of the volunteers: an exploration of the influences that volunteer experiences have on the resilience and sustainability of catchment groups in coastal queensland (PhD Thesis).

Gordon, L., Folke, C., 2000. Ecohydrological landscape management for human well-being. Water international 25, 178–184.

Gray, I., Lawrence, G., 2001. Neoliberalism, individualism and prospects for regional renewal. Rural Society 11, 283–298.

Griffin NRM, URS Australia Pty Ltd, 2001. An evaluation of investment in Landcare support projects (Final report for Agriculture, Fisheries and Forestry Australia). URS Australia Pty Ltd and Griffin NRM.

Gunningham, N., 2002. Reconfiguring environmental regulation: the future public policy agenda.

Gunningham, N., Kagan, R.A., Thornton, D., 2004. Social license and environmental protection: why businesses go beyond compliance. Law & Social Inquiry 29, 307–341.

Hamilton, C., 1995. The economic language of Landcare (Background Paper No. 3). The Australia Institute, Canberra, ACT.

Hawthorne, M., Alabaster, T., 1999. Citizen 2000: development of a model of environmental citizenship. Global Environmental Change 9, 25–43.

Healthy Parks Healthy People. The health benefits of contact with nature in a park context. A review of current literature. (Occassional Paper series No. Volume 1), 2002. . Faculty of Health and Behavioural Sciences, Melbourne.

Hegney, D., Ross, H., Baker, P., Buikstra, E., King, C., McLachlan, K., Rogers-Clark, C., Stallard, L., Watson-Luke, A., 2008a. Identification of personal and community resilience that enhance psychological wellness: A Stanthorpe study. Australian Research Council, Canberra, ACT.

Hegney, D., Ross, H., Baker, P., Rogers-Clark, C., King, C., Buikstra, E., Watson-Luke, A., McLachlan, K., Stallard, L., 2008b. Building resilience in rural communities (Toolkit). The University of Queensland and University of Southern Queensland, Toowoomba, Queensland.

Hill, R., Williams, L., Lane, M., Robinson, C., Taylor, B., 2009. Indigenous natural resource management: overcoming marginalisation produced in Australia's current NRM model. Contested country: local and regional environmental management in Australia 161–178.

Hogan, L., Cumming, B., 1997. More than a question of numbers: Working with women and people from a non-English speaking background toward total catchment participation (Report of the Landcare Participation Project). Department of Primary Industries and Energy, and the Department of Natural Resources and Environment, Canberra, ACT.

Horvath, D. (Ed.), 2001. NSW Decade of Landcare Evaluation. NSW Department of Land and Water Conservation, Sydney, NSW.

Horwitz, P., Lindsay, M., O'Connor, M., 2001. Biodiversity, endemism, sense of place, and public health: Inter-relationships for Australian inland aquatic systems. Ecosystem Health 7, 253–265.

Johnston, F., Burgess, P., Bowman, D.M.J.S., 2007. Chapter 15 - A case for Indigenous natural resource management and health, in: Luckert, M.K., Campbell, B.M., Gorman, J.T., Garnett, S.T. (Eds.), Investing in Indigenous Natural Resource Management. Charles Darwin University Press, Darwin, NT.

Kellert, S.R., Derr, V., 1998. A national study of outdoor wilderness experience. Unpublished report. New Haven, CT: Yale University.

Kingsley, J., Townsend, M., Phillips, R., Aldous, D., 2009. "If the land is healthy … it makes the people healthy": The relationship between caring for Country and health for the Yorta Yorta Nation, Boonwurrung and Bangerang Tribes. Health & Place 15, 291–299.

Koss, R.S., Kingsley, J.Y., 2010. Volunteer health and emotional wellbeing in marine protected areas. Ocean & Coastal Management 53, 447–453.

Land & Water Australia, 2003. National Landcare Program Review. Land & Water Australia.

Landcare Network Victoria, Landcare Victoria, 2004. Making community Landcare even stronger: Victorian Landcare Forum report. Presented at the 2004 Victorian Landcare Forum, Victorian Government Department of Sustainability and Environment, Bendigo, Victoria, p. 68.

Landcare Network Victoria, Landcare Victoria, 2005. Making community Landcare even stronger: Stories of success. Presented at the 2004 Victorian Landcare Forum, Victorian Government Department of Sustainability and Environment, Bendigo, Victoria, p. 68.

Landcare Victoria, Department of Sustainability and Environment, 2008a. Shaping our future: Proceedings. Presented at the 2008 Victorian Landcare Forum, Landcare Victoria, Creswick, Victoria, p. 21.

Landcare Victoria, Department of Sustainability and Environment, 2008b. Shaping our future: Proceedings. Presented at the 2008 Victorian Landcare Forum, Landcare Victoria, Creswick, Victoria, p. 28.

Landcare Victoria, Department of Sustainability and Environment, 2008c. Shaping our future: Stories. Presented at the 2008 Victorian Landcare Forum, Landcare Victoria, Creswick, Victoria, p. 42.

Landcare Victoria, Department of Sustainability and Environment, 2008d. Shaping our future: Stories. Presented at the 2008 Victorian Landcare Forum, Landcare Victoria, Creswick, Victoria, p. 34.

Lane, M.B., 2002. Buying back and caring for country: institutional arrangements and possibilities for indigenous lands management in Australia. Society &Natural Resources 15, 827–846.

Lauber, T.B., Decker, D.J., Knuth, B.A., 2008. Social networks and community-based natural resource management. Environmental management 42, 677–687.

Lloyd, D.L., Johnson, B., O'Brien, S.M., Lawrence, D.N., 2009. Action learning in partnership with Landcare and catchment management groups to support increased pasture sowings in southern inland Queensland. Animal Production Science 49, 907.

Lockie, S., 1998. Landcare in Australia: Cultural transformation in the management of rural environments. Culture & Agriculture 20, 21–29.

Lockie, S., 2001. Chapter 20 - Community development management? Landcare in Australia, in: Lockie, S., Bourke, L. (Eds.), Rurality Bites. Pluto Press, Annandale, NSW.

Lockie, S., 2003. Conditions for building social capital and community well-being through plantation forestry. Australian Forestry 66, 24–29.

Lockie, S.D., 1996. Sociocultural dynamics and the development of the landcare movement in Australia (PhD Thesis).

Love, C., 2012. Evolution of Landcare in Australia: In the context of Australian Government natural resource management policy and programs (Perspectives from the former National Landcare Facilitator). Australian Landcare Council Secretariat.

Lovell, C., Mandondo, A., Moriarty, P., 2003. The question of scale in integrated natural resource management. Integrated natural resource management. Linking productivity, the environment and development. CABI, Wallingford 109–136.

MacGregor, S., Pardoe, S., Dobson, A., Bell, D., 2005. Environmental Citizenship: the Goodenough primer (Final Seminar). Goodenough College, London, UK.

Maller, C., Townsend, M., Brown, P., St Leger, L., 2002. Healthy parks healthy people: The health benefits of contact with nature in a park context. Burwood (Australia): Faculty of Health and Behavioural Sciences, Deakin University.

Maller, C., Townsend, M., St Leger, L., Henderson-Wilson, C., Pryor, A., Prosser, L., Moore, M., 2008. Healthy parks, healthy people. The health benefits of contact with nature in a park context. A review of relevent literature., 2nd edition. Melbourne, Victoria.

McDermott, R., O'Dea, K., Rowley, K., Knight, S., Burgess, P., 2008. Beneficial impact of the Homelands Movement on health outcomes in central Australian Aborigines. Australian and New Zealand Journal of Public Health 22, 653–658.

McTernan, R., Scully, M.-A., 2010. Finding common ground: A review of Indigenous engagement in Landcare in Victoria. Victorian Government Department of Sustainability and Environment, Melbourne, Victoria.

Mobbs, C., Dovers, S., 1999. Social, economic, legal, policy and institutional R&D for natural resource management: Issues and directions for LWRRDC. Occasional paper 1, 99.

Moore, S.A., 2005. Regional delivery of natural resource management in Australia: Is it democratic and does it matter. Participation and governance in regional development: global trends in an Australian context 121–136.

Moore, S.A., Jennings, S.F., Tacey, W.H., 2001. Achieving sustainable natural resource management outcomes on the ground: the key elements of stakeholder involvement. Australasian Journal Of Environmental Management 8, 91–98.

Moore, S.A., Rockloff, S.F., 2006. Organizing regionally for natural resource management in Australia: reflections on agency and government. Journal of Environmental Policy & Planning 8, 259–277.

Mues, C., Chapman, L., Van Hilst, R., 1998. Promoting improved land management practices on Australian farms: A survey of landcare and land management related programs (Research report No. 98.4), Landcare. Australian Bureau of Agricultural and Resource Economics, Canberra, ACT.

Mullen, J.D., 2001. An economic perspective on land degredation issues (Economic Research Report No. 9). NSW Agriculture, Orange, NSW.

Newby, J.C., Cramb, R.A., 2009. Living on the margin: Assessing the economic impacts of Landcare in the Philippine uplands, in: Proceedings of the 53rd Annual Conference. Presented at the Australian Agricultural and Resource Economics Society, Australian Agricultural and Resource Economics Society, Cairns, Queensland, p. 29.

Newby, J.C., Cramb, R.A., 2011. Economic impacts of conservation farming in a marginal environment: the case of "Landcare" in the Philippines. International Journal of Agricultural Sustainability 9, 456–470.

Nicholson, C., Knight, A., 2003. Auditing 10 years of Landcare in the Woady Yaloak catchment: How are we progressing along the triple bottom line? Presented at the National Landcare Conference, Darwin, NT, p. 7.

Pannell, D.J., 2001. Dryland salinity: economic, scientific, social and policy dimensions. Australian Journal of Agricultural and Resource Economics 45, 517–546.

Parkes, M., Panelli, R., 2001. Integrating catchment ecosystems and community health: the value of participatory action research. Ecosystem Health 7, 85–106.

Paton, S., Curtis, A., McDonald, G., Woods, M., 2004. Regional natural resource management: is it sustainable. Australasian Journal of Environmental Management 11, 259–267.

Pepperdine, S., Ewing, S., 2001. Integrating social sustainability considerations into natural resource management. Lockie, S. Higgins, V. and Lawrence, G.(eds). Environment, Society and Natural Resource Management: Theoretical Perspectives from Australasia and the Americas 70–83.

Petheram, R.J., Clark, R.A., 1998. Farming systems research: relevance to Australia. Animal Production Science 38, 101–115.

PIRSA Sustainable Resources Group, 2000. School and community cooperative landcare projects: Best practice manual. Primary Industries and Resources South Australia, Adelaide, SA.

Pretty, G., Bishop, B., Fisher, A., Sonn, C., 2007. Psychological sense of community and its relevance to well-being and everyday life in Australia. The Australian Community Psychologist 19, 6–25.

Pretty, J., 2003. Social capital and the collective management of resources. Science 302, 1912–1914.

Pretty, J., Smith, D., 2004. Social capital in biodiversity conservation and management. Conservation Biology 18, 631–638.

Pretty, J., Ward, H., 2001. Social capital and the environment. World development 29, 209–227.

Putnam, R.D., 1993. The prosperous community. The american prospect 4, 35–42.

Raymond, C.M., Bryan, B.A., MacDonald, D.H., Cast, A., Strathearn, S., Grandgirard, A., Kalivas, T., 2009. Mapping community values for natural capital and ecosystem services. Ecological Economics 68, 1301–1315.

Ritchie, H., 1995. Landcare in the deregulated rural economy of New Zealand. Rural Society 5, 67–75.

Roughley, R., Williams, S., 2009. The engagement of Indigenous Australians in natural resource management: Key findings and outcomes from Land & Water Australia and the broader literature (Final report to Land & Water Australia). Land & Water Australia, Canberra, ACT.

Sobels, J., Curtis, A., 2001a. Second generation Landcare support in West Gippsland (report for Landcare Victoria No. 156). Johnstone Centre, Charles Sturt University, Albury, NSW.

Sobels, J., Curtis, A., 2001b. Landcare networks: Establishing viable local organisations in rural Australia. Natural Resource Management 4, 22–30.

Sobels, J., Curtis, A., Lockie, S., 2001. The role of Landcare group networks in rural Australia: Exploring the contribution of social capital. Journal of Rural Studies 17, 265–276.

Speldewinde, P.C., Cook, A., Davies, P., Weinstein, P., 2009. A relationship between environmental degradation and mental health in rural Western Australia. Health & Place 15, 880–887.

Speldewinde, P.C., Cook, A., Davies, P., Weinstein, P., 2011. The hidden health burden of environmental degradation: disease comorbidities and dryland salinity. Ecohealth 8, 82–92.

Stern, P.C., 2000. New environmental theories: toward a coherent theory of environmentally significant behavior. Journal of Social Issues 56, 407–424.

Townsend, M., Weerasuriya, R., 2010. Beyond blue to green: The benefits of contact with nature for mental health and well-being. Beyond Blue Limited, Melbourne, Victoria.

Toyne, P., Farley, R., 2000. The decade of Landcare: Looking backward - looking forward (Discussion Paper No. 30). The Australia Institute.

Vanclay, F., 2004. Social principles for agricultural extension to assist in the promotion of natural resource management. Animal Production Science 44, 213–222.

Vanclay, F.M., Lockie, S. (Eds.), 2000. Critical Landcare, Key papers series. Centre for Rural Social Research, Charles Sturt University, Wagga Wagga, NSW.

Walker, R.H., 2000. The National Landcare Program: What has it changed? (A compilation of evaluations of the National Landcare Program 1992-1998). Department of Agriculture, Fisheries and Forestry, Canberra, ACT.

Webb, T., Cary, J., 2005. Social capital and natural resource management: An application to Landcare. Rural Society 15, 119–131.

Webb, T.J., Cary, J., 2009. Australia: Social capital and natural resource management - the Australian Landcare movement, in: Rivera, W., Alex, G. (Eds.), Revitalization Within Public Sector Services: Case Studies of International Initiatives, Agriculture and Rural Development Discussion Paper 11: Extension Reform for Rural Development. The World Bank, Washington, D.C., pp. 5–11.

Weir, J.K., Stacey, C., Youngetob, K., 2011. The benefits of caring for country (Report for the Department of Sustainability, Environment, Water, Population and Communities). Australian Institute of Aboriginal and Torres Strait Islander Studies, Canberra, ACT.

Westermann, O., Ashby, J., Pretty, J., 2005. Gender and social capital: The importance of gender differences for the maturity and effectiveness of natural resource management groups. World Development 33, 1783–1799.

Wilson, G.A., 2004. The Australian Landcare movement: Towards "post-productivist" rural governance? Journal of Rural Studies 20, 461–484.

Woodhill, J., 2010. Sustainability, Social Learning and the Democratic Imperative: Lessons from the Australian Landcare Movement, in: Blackmore, C. (Ed.), Social Learning Systems and Communities of Practice. Springer London, pp. 57–72.

Youl, R. (Ed.), 2006. Landcare in Victoria: How Landcare Helped People, Government and Business Work Together in Victoria - a Collection. R. Youl, South Melbourne, Victoria.

Youl, R., Marriott, S., Nabben, T., 2006. Landcare in Australia: Founded on Local Action, 5th ed. SILC, Melbourne, Victoria.

# Appendix C Interview questionnaire

## Interview template – Multiple Benefits of Landcare

**Date:**

**Participant name:**

**Participant organisation:**

**Participant role:**

**Introduction**

**About the project:**

GHD has been commissioned by the Australian Landcare Council (ALC) and the Department of Agriculture to undertake a study on the multiple benefits from natural resource management (NRM) and Landcare projects and activities.

In addition to their documented production and environmental benefits, these projects and activities contribute to the health and resilience of our communities in ways that are currently not recognised, that is they deliver multiple benefits. The multiple benefits can include:

- Economic
- Education
- Health
- Resilience
- Social Capital

**Project requirements:**

Specific questions that the study should answer are:

What are the multiple outcomes and benefits (social, economic, cultural, health, education and community) that result from NRM and Landcare activities?

How do these outcomes and benefits contribute to building community resilience and capacity to handle major challenges such as natural disasters (e.g. fire/flood/drought/cyclone/storm surge), food security, climate change, water management, declining regional populations?

How can multiple benefits and outcomes be monitored in the future to demonstrate returns on NRM/Landcare investment?

How should the multiple benefits and outcomes be communicated to agencies and organisations outside the NRM sector?

**About the interview:**

The semi-structured interviews will seek to gain a detailed understanding of the social, economic, cultural, health, education and community outcomes and benefits and how these contribute to building community resilience and capacity to handle major challenges as identified in the literature review.

Interview participants (identified with the assistance of the Council) will cover the following sectors:

- Agencies
- Regional NRM groups
- Landcare networks and associations
- Educational institutions
- Non-government organisations
- Regional environmental and farming groups
- Local government and community groups
- Commercial organisations
- International Landcare projects
- Prominent individuals with long term knowledge of NRM and Landcare program development

The phone interview questions will be segmented into the various categories of multiple benefits.

**Purpose of interview:**

- Verification of the literature review findings – categories of multiple benefits
- Establishment of classification (criteria), indicators and verifiers of multiple benefits

**Hierarchy Structure of Multiple Benefits:**

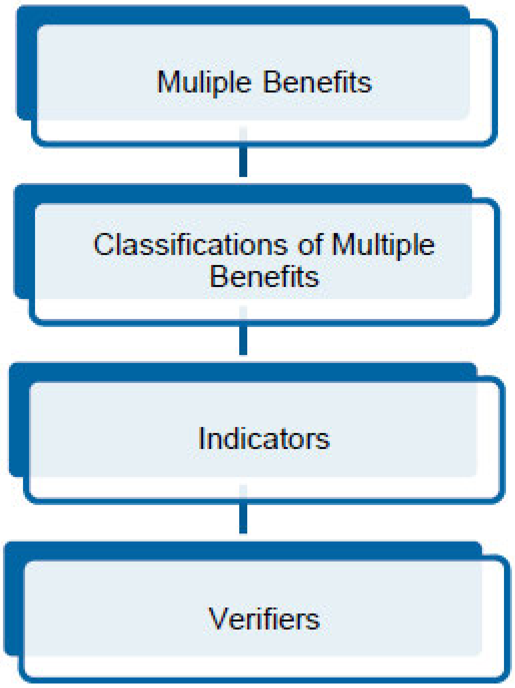


**Questions:**

Requirements:

- Detail the relationship between Landcare programs/activities and various sectors
- Determine multiple benefits of Landcare programs/activities
- Detail how each of the multiple benefits contributes to building community resilience and capacity to handle major challenges
- Uncover measurements of each classification of multiple benefits. These must be measurable 'by numbers', easily replicated, and complement existing NRM performance indices

**Section 1: Background**

Determine:

- Involvement in/with Landcare
- Alignment with program objectives
- Strength of relationship with Landcare groups

| Section | Question | Response |
|---|---|---|
| **1.1** | What is your involvement with Landcare[7]? (Participant, co-coordinator, research, partner, or sponsor)?<br>If 'yes' to partner, how?<br>Any comments? | ☐Participant ☐Co-ordinator<br>☐Researcher ☐Partner<br>☐Sponsor ☐Other |
| **1.2** | What Landcare groups, projects or activities are you involved in? | — |
| **1.3** | How do Landcare groups, projects or activities align with your organisations objectives? | — |
| **1.4** | How would you define the strength of your relationship with Landcare groups?<br>Any comments? | ☐Strong ☐Good ☐Poor<br>☐Non-existent |

**Section 2: Multiple Benefits**

Determine:

- Examples of multiple benefits
- How each of the multiple benefits contributes to building community resilience and capacity to handle major challenges?
- How multiple benefits can be measured?

Note to interviewer: Explain and define multiple benefits (excluding environmental)

| Section | Question | Response |
|---|---|---|
| **2.1** | Please provide examples of multiple benefits. (*categorise where possible*)<br>• *Economic*<br>• *Education*<br>• *Health*<br>• *Resilience*<br>• *Social Capital* | — |
| **2.2** | How do these build capacity to handle major challenges (such as natural disasters)? | — |

[7] Landcare refers to all _care' organisations involved in natural resource management

| Section | Question | Response |
|---|---|---|
| **2.3** | How can these be measured?<br>(This question seeks to identify indicators – Refer to examples of indicators) | — |

*Refer to definitions of multiple benefits

**Section 3: Monitoring and Verifying Multiple Benefits and Outcomes**

Determine:

- Methods of monitoring and evaluation which demonstrate ROI in NRM – can these be applied to Landcare?
- Adapt / assess existing NRM monitoring which could be used for Landcare/NRM Multiple Benefits

| Section | Question | Response |
|---|---|---|
| **3.1** | Can you provide examples of methods used to monitor the indicators provided in section 2.3? | — |
| **3.2** | Are any of the following methods used by your organisation to monitor activities associated with Landcare?<br>• Economic indicators<br>• Education indicators<br>• Health indicators<br>• Resilience indicators<br>• Social Capital indicators<br>Any comments? | — |
| **3.3** | Can any of the following methods of monitoring and evaluation be applied to Landcare activities?<br>• Economic indicators<br>• Education indicators<br>• Health indicators<br>• Resilience indicators<br>• Social Capital indicators<br>Any comments? | — |

*Refer to examples of indicators

Examples of indicators (e.g. NRM performance indices)

**Section 4: Communicating Multiple Benefits and Outcomes to agencies and organisations outside the NRM sector**

Determine:

- Best methods of communication to agencies and organisations?
- What communication mechanisms have worked in the past to secure funding?

| Section | Question | Response |
|---|---|---|
| **4.1** | What communication methods best demonstrate NRM program/activity benefits to/from outside agencies and organisations? | — |
| **4.2** | What mechanisms of communication have you used/been presented in the past which have been successful in demonstrating program/activity benefits to secure funding? | — |

Examples of communication methods include:

- guidelines, protocols and policies
- research papers
- collaborative approaches
- articles for magazines or scientific and professional journals
- newsletters
- websites
- fact sheets, flyers, brochures, posters
- photographic material
- multimedia productions

# Appendix D Consultation list

| Sector | Organisation | Contact Name |
|---|---|---|
| Agency | Cradle Coast NRM, Tasmania | Spencer Gibbs |
| Agency | DPI NSW | William Hawkins |
| Agency | DAF WA | Natalie More |
| Agency | Victorian Catchment Management Council | Tracey Koper |
| Educational Institution | Johnstone Centre, CSU Albury | Prof. Allan Curtis |
| Educational Institution | SARDI and Eyre Peninsula Landcare | Linden Masters |
| Educational Institution | UNE | Dr Julian Prior |
| Educational Institution | UQ Landcare Community Resilience | Helen Ross |
| Farming Groups | National Farmers Federation | Debb Kerr |
| Local government or community group | Mitchell and District Landcare | Jenny Hockey |
| Local government or community group | Southern New England Landcare | Sonia Williams |
| Local government or community group | Tasmanian Landcare | Margie Jenkin |
| Non-government organisation | Australian Conservation Foundation | Confidential |
| Non-government organisation | Greening Australia | Chris Andrew |
| Non-government organisation | National Landcare Facilitator | Brett De Hayr |
| Regional environmental group | Coastcare Victoria / DSE Victoria | Amanda Cornish |
| Prominent individual | Charles Darwin University | Prof. Andrew Campbell |
| Prominent individual | 2010 RIRDC Australian Rural Woman of the Year | Sue Middleton |
| Prominent individual | Remarkable NRM Consulting | John Galvin |
| Prominent individual | Holbrook Landcare Network | Chris Cumming |

# Appendix E Case study framework

## Case Study template – Multiple Benefits of Landcare

**Date:**

**Participant name(s):**

**Participant organisation(s):**

**Participant role:**

**Introduction**

**About the project:**

GHD has been commissioned by the Australian Landcare Council (ALC) and the Department of Agriculture to undertake a study on the multiple benefits from natural resource management (NRM) and Landcare projects and activities.

In addition to their documented production and environmental benefits, these projects and activities contribute to the health and resilience of our communities in ways that are currently not recognised, that is they deliver multiple benefits. The multiple benefits can include:

- Social – attitudes, wellbeing, sense of purpose, flow-on events and maintenance of values
- Economic – flow-on effects
- Cultural – maintenance of cultural values or assets, ongoing cultural activities
- Health – mental and physical health benefits
- Education – sustainability and environmental values, engagement of children
- Community – recreation, safety

**Project requirements:**

Specific questions that the study should answer are:

What are the multiple outcomes and benefits (social, economic, cultural, health, education and community) that result from NRM and Landcare activities?

How do these outcomes and benefits contribute to building community resilience and capacity to handle major challenges such as natural disasters (e.g. fire/flood/drought/cyclone/storm surge), food security, climate change, water management, declining regional populations?

How can multiple benefits and outcomes be monitored in the future to demonstrate returns on NRM/Landcare investment?

How should the multiple benefits and outcomes be communicated to agencies and organisations outside the NRM sector?

**Case study methodology:**

Case studies will be undertaken using a consistent format, listing the procedures for how data is collected at the desktop level and how data is verified at site visits.

The initial step in undertaking the case studies will be the compilation of a desktop review of each activity/project/program. This will involve the review of activity outcomes in response to the aims and objectives identified. The desktop review will also require the examination of any relevant data and literature which will assist in developing an initial case study summary.

Case study site visits will then be undertaken to verify the interview and desktop review findings. GHD will complete site verification through field work for five case studies. An exception is the instance where the site is remote and Department of Agriculture/ACL agrees the site is of high importance which can be detailed without a site visit (e.g. an International Landcare or remote Northern Australia project).

**Data collection and site visit:**

A data collection sheet will be developed prior to any site visits. The information that will be collected on site will include:

- Detailed verification of interview findings
- Verification of the completion of program activities
- Site inspection to verify validity of multiple benefits and outcomes
- Links to other contacts who may be able to comment on the multiple benefits
- Relevant photographs for use in publications

**Purpose of case study:**

A series of measureable indicators have been developed and will be tested through case studies, which will form the basis of communication material which can be tailored to relevant agencies and other key audiences.

- Verification of the literature review findings – categories of multiple benefits
- Demonstration of classification (criteria), indicators and verifiers of multiple benefits

**Hierarchy Structure of Multiple Benefits:**

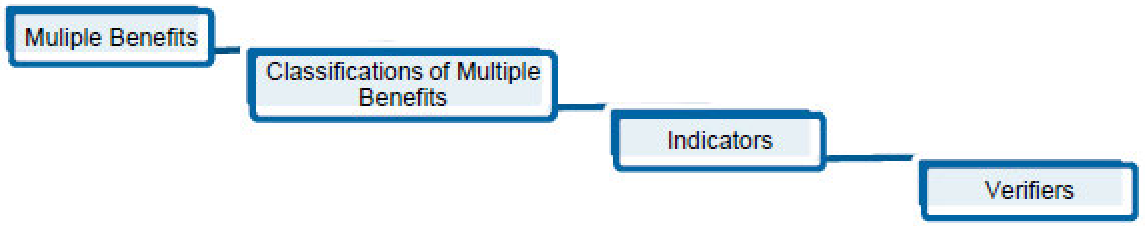

**Desktop review:**

Overview of project/activity, partners and general information (review available information)

Outline of the multiple outcomes and benefits (social, economic, cultural, health, education and community) that result from NRM and Landcare activities

Outline how outcomes and benefits contribute to building community resilience and capacity to handle major challenges such as natural disasters (e.g. fire/flood/drought/cyclone/storm surge), food security, climate change, water management, declining regional populations etc.

Outline how multiple benefits and outcomes can be monitored in the future to demonstrate returns on NRM/Landcare investment (e.g. Social Return on Investment)

Outline how multiple benefits and outcomes should be communicated to agencies and organisations outside the NRM sector (methods of communication and presentation – e.g. newsletters, research papers, reports, journal articles, photographic material, multimedia, and websites)

**Site visit:**

**Detailed verification of interview findings:**

- Verify the multiple benefits and outcomes
- Verify how benefits and outcomes contribute to building community resilience and capacity
- Verify how benefits and outcomes can be monitored in the future to demonstrate returns on investment

- Verify how multiple benefits and outcomes can be communicated to agencies and organisations outside the NRM sector

**Verification of the completion of program activities:**

- View documentation and/or physical evidence of the program/activity
- Verify activities through discussions with appropriate individuals from organisations involved in delivery (Program coordinator, partners)

**Verification of the validity of multiple benefits and outcomes:**

- Physically sight project outcomes or documentation (where possible)
- Verify activities through discussions with appropriate individuals from organisations who can verify the multiple benefits (i.e. local council/community organisations)

**Discussion with other relevant individuals/organisations that may be able to comment on the multiple benefits of projects/activities:**

- Discussions with appropriate individuals from organisations who participated or benefited from the program/activity

**Relevant photographs and materials for use in publications:**

- Where possible sight and photograph physical evidence of program/activity
- Where necessary, gain permission to use photographs of any cultural/indigenous sites.
- Obtain materials and seek permission where necessary to publish materials.

# Appendix F Role and membership of the ALC

The Australian Landcare Council (the council) is the Australian Government's key advisory body on Landcare. The council is established under the Commonwealth *Natural Resources Management (Financial Assistance) Act 1992* that identifies the function of the council (Part 13.(2) a) b)) as:

1. to make recommendations to the Minister for Agriculture and the Minister for the Environment on:

    a. matters concerning natural resources management; and

    b. priorities and strategies for natural resources management; and

2. to investigate and report to the Australian Government on matters concerning natural resources management referred to it by either of the Ministers.

In addition to providing advice to the Australian Government to meet the challenges of food security, climate variability, and environmental degradation, the council also supports communication between the Landcare community (which includes farmers, volunteer groups and Indigenous and other land managers) and the government.

The council also supports the implementation and promotion of the principles in the *Australian Framework for Landcare* and the *Community Call for Action* and is responsible for overseeing the five-year (mid-term) review of these two documents on behalf of the Landcare community.

## Membership of the Australian Landcare Council (as at 1 January 2013)

**The Hon. Kim Chance**, from Western Australia, is the chair of the council. Mr Chance is the Proprietor of Gulf Australia Trading and a past member of the Western Australian Legislative Council and former state minister in several portfolios including Agriculture and Food; Fisheries; Mid–West, Wheatbelt, and the Great Southern Development Commissions. Mr Chance brings a wealth of agricultural experience and has a passion for Landcare.

**Ms Kate Andrews**, from the Northern Territory, is the Chair of the Northern Territory Natural Resource Management Board. Ms Andrews has worked in a wide variety of capacities in natural resource management for nearly two decades, was previously Knowledge and Adoption Manager for Land & Water Australia and has assisted communities within the Lake Eyre Basin to design a multi–state natural resource management organisation.

**Mr Ron Archer**, from Dimbulah in Queensland, is a Djungan Elder whose traditional name is Jun-ju-lud (small bird). Mr Archer is the coordinator of the Northern Gulf Indigenous Savannah Group, the Chairman of Ngudda-bul-gan Tribal Aboriginal Corporation, the Indigenous representative on the Northern Australia Beef Industry Working Group, a member of the North Australia Indigenous Land and Sea Management Alliance and a Senior Lecturer in Cultural Awareness Relationship and Partnership Development. Mr Archer believes 'connection to country' is everyone's business and 'caring for country' is the duty of the chosen.

**Professor Snow Barlow**, from Victoria, is Convener of the Primary Industries Adaptation Research Network at Melbourne University where his research focuses on the impacts of climate change and adaptation of agricultural industries. Professor Barlow represents the council on the Non-Government Organisation Roundtable on Climate Change and is a member of the Minister for Agriculture, Fisheries and Forestry, Joe Ludwig's Land Sector Working Group. Professor Barlow is also Chair of the Victorian Endowment for Science Knowledge and Innovation and a Director of the Australian Rural Leadership Foundation.

**Dr Pamela Brook**, from Bangalow, runs a macadamia farm at St Helena, near Byron Bay, New South Wales, and is co–founder of an award–winning, value–adding business based on the farm's produce. Dr Brook is also a former director of the Northern Rivers Regional Development Board and is the current Chair of Northern Rivers Food.

**Ms Alexandra Gartmann**, from Bendigo in Victoria, is Chief Executive Officer of the Foundation for Rural and Regional Renewal. She is Chair of the CSIRO Sustainable Agriculture Flagships Advisory Council and former Chief Executive Officer of the Birchip Cropping Group (2001–2011). Ms Gartmann was awarded the 2005 Equity Trustees Not for Profit CEO of the Year for Significant Innovation award. She is a member of the Victorian Women in Primary Industries Advisory Panel, Victorian Flood Disaster Appeal Panel, Crawford Fund for international agricultural research and Board Member of Rural Finance Corporation, as well as sitting on a number of state and regional committees.

**Dr Judy Henderson AO**, from Repton, is a member of the National Wildlife Corridors Plan Advisory Group. Dr Henderson is also a former board member of the NSW Environment Protection Authority and former chair of the Amsterdam–based Global Reporting Initiative, which is setting global standards for sustainability reporting. In 1998 Dr Henderson was appointed an Officer of General Division of the Order of Australia.

**Dr Rosemary Hill**, from Cairns in Queensland, is a Senior Research Scientist for CSIRO Ecosystem Sciences and currently leads several research projects for CSIRO. She has extensive experience in the theory and practice of natural resource management, with a focus on biodiversity and Indigenous country–based planning systems. Dr Hill is Vice-President of the Australian Conservation Foundation, a Board Member of Ecotrust Australia and a Member of the World Commission on Protected Areas and the IUCN Commission on Environment, Economic and Social Policy.

**Mrs Jackie Jarvis**, of Western Australia, is the State Manager of MADEC Harvest Labour Services, a not-for-profit business that sources harvest labour for the agricultural industry. She is also a director of vineyard and wine production business Jarvis Estate, a former director of the Foundation for Australian Agricultural Woman and former board member of the National Rural Woman's Coalition. An active member of her local community, Mrs Jarvis was a WA finalist in the 2010 RIRDC Rural Women's Award.

**Ms Kate Jones**, from Brisbane, is a former Queensland Government Minister for Climate Change, Sustainability, Environment and Natural Resources. Ms Jones is currently completing a Master of Environmental Law and is a member of a number of community groups, including the Ashgrove Climate Change Action Group.

**Mr Jock Laurie**, from Walcha, is a fourth–generation grazier, President of the National Farmers' Federation and a former president of the NSW Farmers Federation.

**Ms Banduk Marika**, from Nhulunbuy, Northern Territory is a renowned artist and has held positions on the boards of the National Gallery of Australia and the Museums and Art

Galleries of the Northern Territory. Ms Marika's work with community groups in Yirrkala, Arnhem Land has resulted in a strong environmental ethic in her community and the development of a form of Landcare which maintains traditional land management whilst embracing contemporary environmental techniques.

**Mr John McQuilten**, from Betley, served as the Member for Ballarat on the Victorian Legislative Council from 1999 until 2006. During his term, Mr McQuilten also served on the Rural and Regional Development Committee, the Premier's Bill Committee and as Chair of the Arts Committee. Mr McQuilten is a member of the University of Ballarat Council and a former member of the Victorian Regional Channel Authority, and of the Murray River National Parks Review Committee.

**Mr Dennis Mutton**, from South Australia, is the former Chair of the South Australian Natural Resource Management Council and former Chief Executive Officer for the South Australian Departments of Primary Industries & Resources, Environment and Natural Resources and Woods and Forests. Mr Mutton also held positions as Deputy President and Commissioner of the Murray Darling Basin Commission and Director of the Australian Rural Leadership Foundation. Mr Mutton is currently an independent consultant in natural resource management, leadership development and strategic management of research and development and Chair of South Australia's Native Vegetation Council.

**Ms Vicki–Jo Russell AM**, from Adelaide, is a member of several regional, state and national Natural Resource Management boards and Ministerial advisory committees. Ms Russell has a strong history in community engagement and extensive experience in conservation and biodiversity management. Ms Russell is involved in national and state biodiversity planning and program development and received a Member of the Order of Australia in 2003 for her contribution to community–based conservation in South Australia.

**Ms Sharon Starick**, from Cambrai, South Australia, operates a commercial farm business, and is the former Chair of the South Australia Natural Resources Committee for the South Australia Farmers Federation and the Natural Resources Management Council. She is the presiding member of the South Australian Murray Darling Basin Natural Resources Management Board, the convenor for the National NRM Regions Working Group and was recently appointed as a Director for the Grains Research and Development Corporation. Ms Starick has a strong background in sustainable agricultural production and previously was a director of Land and Water Australia.

**Mr Andrew Stewart**, from Deans Marsh, Victoria is a coordinator of the Otway Agroforestry Network, an organisation he co–founded in 1993. Mr Stewart also sits on the Australian Master TreeGrower Program's steering committee and manages the grazing property of Yan Yan Gurt West in southern Victoria.

**Ms Lynne Strong**, from Jamberoo on the New South Wales South Coast, became an honorary council member after winning the 2012 Bob Hawke Landcare Award. Ms Strong and her family milk over 500 cows at Clover Hill Dairies, which won the 2010 National Landcare Woolworths Primary Producer Award. Lynne's focus beyond the farm gate is reconnecting consumers with farming communities and communicating that Australian farmers are committed to working towards sustainable farming practices. Lynne is also the founder of and national program director for Art4Agriculture, a network of young people who engage with and empower young people to tell agriculture's story.

**Mr James Walch** farms at Stewarton, Epping Forest situated in the Northern Midlands of Tasmania. He runs a diverse farming operation which includes wool, potatoes, poppies

and irrigated lucerne. Mr Walch has been involved in Landcare activities for the last two decades including riparian restoration, shelter belt and corridor plantings. He has been actively involved in whole farm planning and the development of property management plans. He is an advocate of sustainable and profitable farming systems. Mr Walch is a past president of the Tasmanian Farmers and Graziers Association (TFGA) and currently sits on their environment policy council and is chair of the climate change standing committee.

**Ms Keelen Mailman** is the manager of Mount Tabor Station, near Augathella in Queensland, is a member of South West Natural Resource Management organisation. She is a Native Title applicant, a member of the Bidjara Traditional Owners Group and Director, South West Projects for training for Bidjara Traditional Owners. Ms Mailman was a finalist in the 2007 Queensland Australian of the Year and was recognised in 2005 by the Queensland Museum South Bank as the first Aboriginal women to manage a cattle property in Australia. She participated in the Australian Rural Leadership Program in 2009 and is involved in local community organisations such as Aboriginal health, schools, bush heritage agencies and volunteer work teaching and mentoring children.

**Ms Ella Maesepp**, from Katanning in Western Australia, is the District Landcare Officer with the Katanning Land Conservation District Committee, having previously worked in Landcare roles in the Upper Blackwood, Dinniup, Western Australia. She was the winner of the Environment Category of the 2004 Western Australia Youth Awards and a founding member of the Western Australia Scout Environment Awareness Program. Ms Maesepp lives on her family's broadacre farming property.

GHD

133 Castlereagh St Sydney NSW 2000

© GHD 2013

This document is and shall remain the property of GHD. The document may only be used for the purpose for which it was commissioned and in accordance with the Terms of Engagement for the commission. Unauthorised use of this document in any form whatsoever is prohibited.

N:\AU\Sydney\Projects\21\21673\WP\Report\183081-Final Aug 2013 (Revised Ex Summary).docx

Document Status

| Rev No. | Author | Reviewer - Name | Reviewer - Signature | Approved for Issue - Name | Approved for Issue - Signature | Approved for Issue - Date |
|---|---|---|---|---|---|---|
| 1 | Tim Ferraro | Sue Salvin | | Sue Salvin | | 11/01/2013 |
| 2 | Tim Ferraro | Sue Salvin | | Sue Salvin | | 11/02/2013 |
| 3 | Tim Ferraro | Tim Ferraro | | Tim Ferraro | | 07/07/2013 |

www.ghd.com
