# Supplementary material for: Healthy Patients, Workforce and Environment: Coupling Climate Adaptation and Mitigation to Wellbeing in Healthcare
Source: Int J Environ Res Public Health. 2023 Nov 13;20(22):7059. doi: 10.3390/ijerph20227059 (PMC10671525; doi:10.3390/ijerph20227059)
Supplement: Supplementary file 1 [file ijerph-20-07059-s001.zip › Permission GHD Multiple Benefits of Landcare.pdf]

Emily Ray <Emily.Ray@ghd.com>

Reply all

Wed 26/07, 7:45 PM

Mark De Souza;

Nicola Bailey <Nicola.Bailey@ghd.com>

multiple-benefits-of-landcare-and-natural-resource-management-report.pdf1 MB

GHD Response to RFO - Multiple Benefits.pdf2 MB

Show all 2 attachments (3 MB) Download all

Action Items

CAUTION: This email originated from outside of the organisation. Do not click links or open attachments unless you recognise the sender and know the content is safe.

Hi Mark

Hope you are well.

Our Adelaide office has passed on your query below. Confirming that GHD is happy for you to reproduce and acknowledge the below table, noting the report IP actually rests with DAFF and they have confirmed they are happy to proceed as per below.

Many thanks and best of luck with your article
Emily

Emily Ray | A GHD Principal
B. Ag Ec (Hons)
Sydney Market Leader – Environment
Technical Director – Natural Resources & Agriculture

GHD
Proudly employee-owned | ghd.com
Level 15, 133 Castlereagh Street Sydney NSW 2000 Australia
D +61 2 9239 7018 **M** +61 459 823 502 **E** emily.ray@ghd.com

The Power of Commitment

Connect

Please consider the environment before printing this email

From: Mark De Souza <Mark.DeSouza@nt.gov.au>
Sent: Tuesday, July 25, 2023 10:56 AM
To: Adlmail <adlmail@ghd.com>
Subject: Attention Jeziel permission to use published table from a GHD document on the benefits of Landcare

You don't often get email from mark.desouza@nt.gov.au. Learn why this is important

Dear Jeziel

As discussed, I am urgently seeking permission from GHD to reproduce and acknowledge a table from a report on the benefits of Landcare.

agriculture.gov.au

This is for a journal article promoting the benefits of engaging in landcare on Australian healthcare campuses (see web request below) .

Best regards

Mark

———————————————————-

Dr Mark de Souza

Chair, Sustainable Healthcare Committee (Top End Region NT Health)

Senior Staff Specialist in Emergency Medicine

Royal Darwin and Palmerston Regional Hospitals

0428851373

Begin forwarded message:

From: Mark De Souza <mark.desouza@nt.gov.au>
Date: 21 July 2023 at 4:46:45 pm ACST
To: desouza.mt@gmail.com
Subject: Landare benefits permission FW: Webform submission from: Online enquiries [Incident: 230523-000532]

From: CCG <ccg@mailsd.custhelp.com>
Sent: Wednesday, 24 May 2023 5:08 PM
To: Mark De Souza <Mark.DeSouza@nt.gov.au>
Subject: Webform submission from: Online enquiries [Incident: 230523-000532]

CAUTION: This email originated from outside of the organisation. Do not click links or open attachments unless you recognise the sender and know the content is safe.

You recently requested personal assistance from our Support Centre. Below is a summary of your request and our response. Thank you for allowing us to be of service to you.

Subject

Webform submission from: Online enquiries

Response By E-mail (Maz) (24/05/2023 05.37 PM)

Hello Mark

Thank you for contacting the Department of Agriculture, Fisheries and Forestry. The report remains the property of GHD.

Please contact GHD regarding the use of Table 2 on page 21: Contact - GHD

N:\AU\Sydney\Projects\21\21673\WP\Report\183081-Final Aug 2013 (Revised Ex Summary).doc

For further assistance please refer to our website, reply directly to this email, or telephone the department on 1800 900 090 or +61 3 8318 6700 (from outside Australia).

Maz
Biosecurity officer | Client Contact Group | Biosecurity Operations Division

Department of Agriculture, Fisheries and Forestry
18 Marcus Clarke Street, Canberra ACT 2601 Australia
GPO Box 858 Canberra ACT 2601 Australia

agriculture.gov.au

Customer By Service Email (DAFF) (23/05/2023 04.05 PM)

My enquiry is about:
ABARES (scientific and economic advice)

Comments
I am seeking copyright permission from the NLAC to reproduce a table in a journal article that I am writing concerning the wellbeing benefits of engaging in landcare practices in health care precincts.. The article is to be published in the International Journal of

Environmental Research and Public Health.

The table concerned is Page 21, Table 2 page "categories of multiple benefits" from the 2013 Final Report for the Australian Landcare Council (Multiple Benefits of Landcare and Natural Resource Management), produced by the GHD Group.

I would appreciate an urgent response to meet publication deadlines.

Web page
https://www.agriculture.gov.au/sites/default/files/style%20library/images/daff/__data/assets/pdffile/0009/2386926/multiple-benefits-of-landcare-and-natural-resource-management-report..pdf
Would you like a reply?
Yes

Your name
Dr Mark de Souza

Email
mark.desouza@nt.gov.au

Phone
0428851373

Incident Reference # 230523-000532

Date Created: 23/05/2023 04.05 PM
Status: Resolved

------ IMPORTANT - This email and any attachments have been issued by the Australian Government Department of Agriculture, Fisheries and Forestry. The material transmitted is for the use of the intended recipient only and may contain confidential, legally privileged, copyright or personal information. You should not copy, use or disclose it without authorisation from the Department. It is your responsibility to check any attachments for viruses and defects before opening or forwarding them. If you are not an intended recipient, please contact the sender of this email at once by return email and then delete both messages. Unintended recipients must not copy, use, disclose, rely on or publish this email or attachments. The Department is not liable for any loss or damage resulting from unauthorised use or dissemination of, or any reliance on, this email or attachments. If you have received this e-mail as part of a valid mailing list and no longer want to receive a message such as this one, advise the sender by return e-mail accordingly. This notice should not be deleted or altered ------

[-- 002:002729 :10569--]

CONFIDENTIALITY NOTICE: This email, including any attachments, is confidential and may be privileged. If you are not the intended recipient please notify the sender immediately, and please delete it; you should not copy it or use it for any purpose or disclose its contents to any other person. GHD and its affiliates reserve the right to monitor and modify all email communications through their networks.
